# Design Approaches That Utilize Ionic Interactions to Control Selectivity in Transition Metal Catalysis

**DOI:** 10.1021/acs.chemrev.4c00849

**Published:** 2025-02-28

**Authors:** Hannah K. Adams, Max Kadarauch, Nicholas J. Hodson, Arthur R. Lit, Robert J. Phipps

**Affiliations:** Yusuf Hamied Department of Chemistry, University of Cambridge, Lensfield Road, Cambridge CB2 1EW, United Kingdom

## Abstract

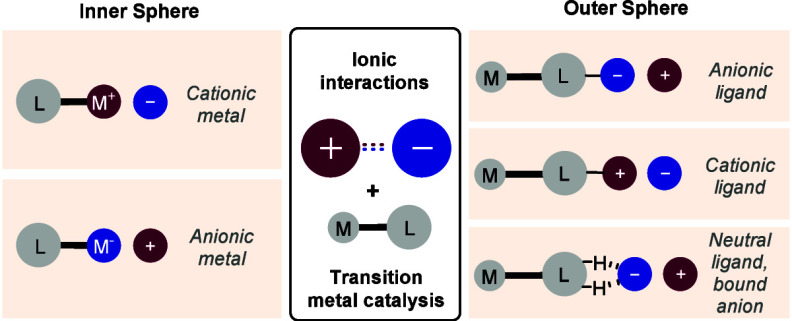

The attractive force between two oppositely charged ions can constitute a powerful design tool in selective catalysis. Enzymes make extensive use of ionic interactions alongside a variety of other noncovalent interactions; recent years have seen synthetic chemists begin to seriously explore these interactions in catalyst designs that also incorporate a reactive transition metal. In isolation, a single ionic interaction exhibits low directionality, but in many successful systems they exist alongside additional interactions which can provide a high degree of organization at the selectivity-determining transition state. Even in situations with a single key interaction, low directionality is not always detrimental, and can even be advantageous, conferring generality to a single catalyst. This Review explores design approaches that utilize ionic interactions to control selectivity in transition metal catalysis. It is divided into two halves: in the first, the ionic interaction occurs in the outer sphere of the metal complex, using a ligand which is charged or bound to an anion; in the second, the metal bears a formal charge, and the ionic interaction is with an associated counterion.

## 1 Introduction

Transition metal complexes can catalyze an immense variety of chemical reactions. The ligand sphere around a metal provides ample opportunity to tune reactivity and selectivity through steric and electronic influence on the metal center and this design principle has been responsible for a plethora of developments that sit at the heart of chemistry’s societal impact. Enzymes use metals in tandem with attractive noncovalent interactions at the active site to precisely position the substrate with respect to the catalytically active metal center.^1^ This positioning can be crucial for control of chemoselectivity, site-selectivity and enantioselectivity. Inspired to some degree by this mode of operation, synthetic chemists have incorporated attractive noncovalent interactions into bifunctional ligands for transition metals where the substrate and metal may be engaged concurrently, sometimes by design and other times through serendipity.^2^ Such ligands, if well matched in structure with the substrate, can provide a high degree of organization during the crucial selectivity-determining steps of the mechanism. Much progress has been made in using hydrogen bonding interactions to make the temporary, noncovalent link between ligand and substrate and advances in this area have been recently covered in a 2022 Chemical Reviews article.^3^ Hydrogen bonding interactions are directional, can be strong with a well-matched donor–acceptor combination, and can be used in pairs to enforce rigidity, factors which have contributed to this being the type of noncovalent interaction that has been most explored for directing catalysis, both with and without transition metals.^4^ In contrast, ionic interactions have been rather less explored, perhaps due to concerns relating to directionality and the associated uncertainty of whether precise outcomes can be achieved with these less well-visualized interactions.^5^ However, in suitably nonpolar solvents, the magnitude of the attractive forces between two opposite charges can be considerable. In solvents of low-to-moderate polarity such as dioxane, toluene, THF or chloroform the achievable interaction energies can be substantially greater than those achieved by many hydrogen bonds, as long as solubility is not an issue. In addition, it is common for other weaker noncovalent interactions to act alongside the ionic interaction which can impart greater directionality. Breakthroughs in the 1980s in which chiral cations were used in asymmetric phase transfer catalysis demonstrated what was possible.^6^ Over the past decades researchers have become more confident in exploring design strategies which utilize ionic interactions and out of this relatively uncharted territory, as is often the case in chemical research, interesting and surprising advances of genuine synthetic utility have arisen.^7^ The focus of this Review will be how this understanding has been combined with the remarkable reactivity of transition metal complexes in the design of new catalytic systems.

One design approach exploits the relatively frequent occurrence of a formal charge on the metal center, which can be either positive or, less commonly, negative. Although the importance of such charged complexes has long been appreciated in catalysis, it is rare for the associated counterion to be considered as an element of the catalyst design, for example to control enantioselectivity. In many cases the counterion may not be depicted in a catalytic cycle as it is considered irrelevant, or its precise identity may not be clear if there are various possible anions present. In the past decades, important advances have been made in moving the counterion (anion or cation) “out of the shadows”, demonstrating the impact the counterion can have at chemistry occurring at the metal center, under the right conditions. Moving away from the established approach of a chiral L-type ligand which remains complexed to the metal through the catalytic cycle was initially a bold move, but the rapid adaptation of this approach has shown that it is, in fact, broadly applicable and capable of solving problems that the conventional approach could not. In the situation that a metal complex itself does not bear a formal charge, ionic interactions can still be utilized by incorporating ionic functionality into the outer ligand sphere through careful design. This can allow an interaction with a substrate to direct subsequent chemistry at the metal or allow association of a chiral counterion. Incorporating chiral information into a ligand by noncovalent association is distinct from the mainstream approach in which this information is covalently incorporated into the ligand scaffold. But emerging strategies demonstrate that this can be effective in situations where conventional ligands have struggled.

**Figure 1:**
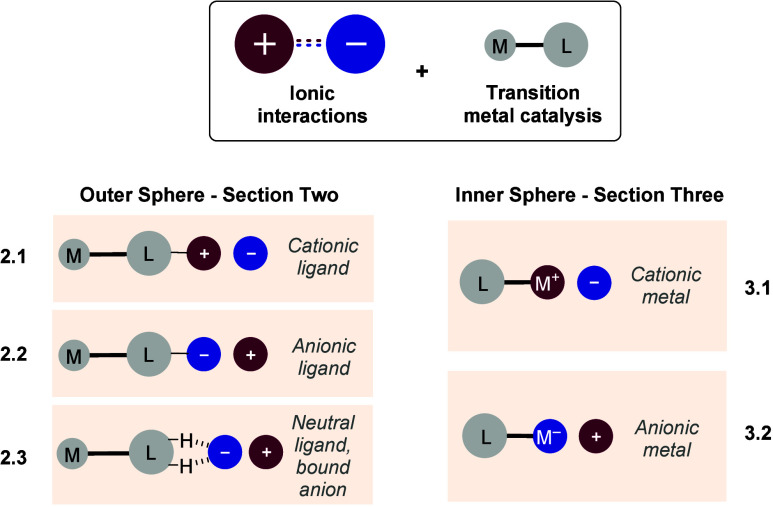
Summary of Review structure.

This Review will examine transition metal catalyzed reactions in which ionic interactions have been proposed to play an important role in the selectivity outcome. The survey will only include examples where two (or more) whole charges are thought to be involved, so will not include ion-dipole or dipole–dipole interactions. It will only cover examples in which an ionic interaction is proposed to significantly impact the selectivity outcome of the reaction, so will not cover those where ionic interactions have been proposed to primarily provide increased rates. To retain focus, the ionic interaction should involve the transition metal complex itself in some manner. Therefore, it will not cover examples in which an uncharged metal complex is involved in a reaction where an ionic interaction is invoked in a different component of the system. The Review will be divided into two main sections. Section Two will cover reactions which invoke an ionic interaction in the outer coordination sphere of the metal complex (Figure 1, left). Section Three will cover reactions which invoke an ionic interaction between a charged transition metal center and an associated counterion (Figure 1, right). Within each subsection the examples will be grouped according to the transition metal used. The primary aim is to survey design approaches to catalytic systems that have utilized ionic interactions as part of that design. Seminal examples will be discussed alongside further applications that demonstrate the evolution of each strategy. We will not necessarily cover all examples in which an ionic interaction has been proposed, after-the-fact, to be involved (as deduced by DFT calculations, for example). There are ambiguities which can make it difficult to precisely define some systems and so absolute certainty of ionic interactions in some cases may not be possible, even if predicted by the guiding hypothesis. For example, when dealing with cationic metal complexes that are associated with chiral anions, there is a widely acknowledged ambiguity relating to whether the counteranion is acting as a “genuine” dissociated counterion or whether it is coordinated to the metal as an anionic ligand. In many reports it is impossible to give a definitive answer, since detailed analysis relating to intermediates may not be available. This aspect will be discussed in more detail at the beginning of Section Three.

There are a number of previous relevant reviews which cover material that will be included here, most prominently three from Toste, Jacobsen and List and their associated co-workers, all of which were first published in 2012.^7^ These reviews covered progress in the use of ion-pairing interactions applied generally, in any type of catalysis (ie. not limited to transition metal catalysis). The application of ion-pairing strategies has expanded considerably in the past decade, hence the narrowing of this review’s focus to transition metal catalysis. There are a number of other related reviews or perspectives which may also be of interest to readers, due to overlap with other catalytic strategies.^2c−2f,5c,8^

## 2 Ionic Interaction in Outer Coordination Sphere

As outlined above, this section will cover the design of reaction systems in which an ionic interaction occurs in the outer coordination sphere of the metal complex. Typically, this occurs through use of a ligand that is functionalized with a charged group, or a group that becomes charged under the reaction conditions, enabling interaction with the substrate or with a chiral counterion. In the former case one can imagine that direct interaction with the substrate could impact selectivity in the ensuing reaction. In the latter case, this offers a different approach to thinking about chiral ligand design, compared with the conventional strategy of covalent incorporation of chiral information into the ligand scaffold. Another situation that will be discussed is when the ligand possesses a functional group that can associate with the counteranion of a substrate through anion binding. Although concerns relating to flexibility in such systems are certainly justified, there are exciting possibilities if sufficient attractive interactions can be harnessed between ligand, counterion and substrate. The section will be split into three sections: the first (2.1) will cover ligands that bear a cationic group, the second (2.2) will cover ligands which bear an anionic group and the third (2.3) neutral ligands that are able to bind to an anion. Each of these subsections will be divided according to the transition metal used in the reaction.

### 2.1 Ligand Bears Cationic Group

#### 2.1.1 Gold

**Figure 2:**
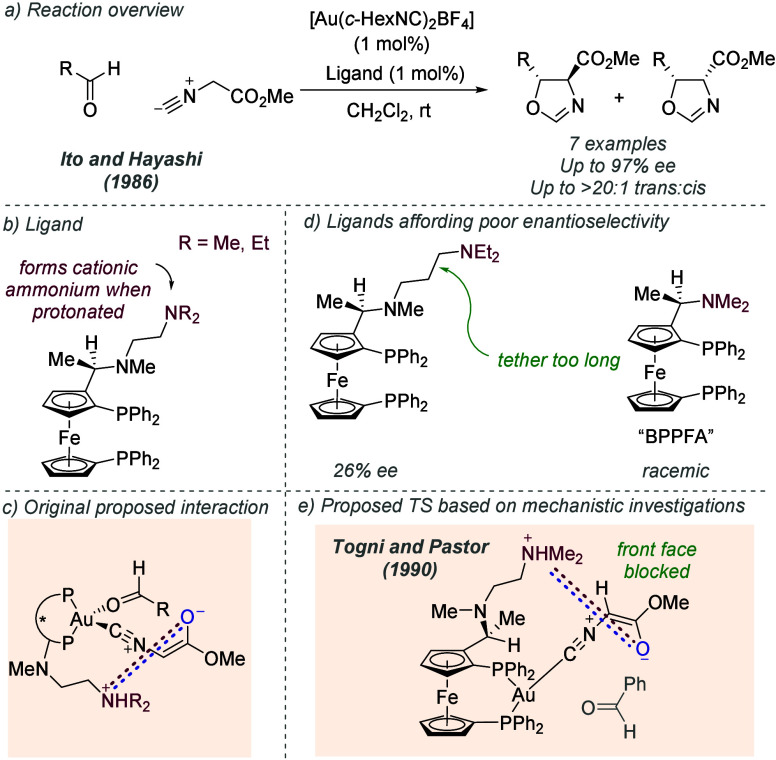
Enantioselective gold-catalyzed aldol reaction of aldehydes and isocyanoacetate deploying an ion pairing interaction between chiral ligand and deprotonated substrate.

In a seminal example of combining ionic interactions with transition metal catalysis in an outer sphere design, Ito, Sawamura and Hayashi in 1986 reported the gold-catalyzed aldol reaction between aldehydes and methyl isocyanoacetate (Figure 2a).^9^ In this reaction, a ferrocene-derived bisphosphine ligand was used which possessed a side chain bearing a tertiary amine, separated by a linker of defined length (Figure 2b). The original work proposed that the tertiary amine would deprotonate the isocyanoacetate reactant, forming an ion-paired enolate, which would associate with the ammonium moiety through ionic interactions and hydrogen bonding (Figure 2c). Complexation of the aldehyde to the gold metal center was envisaged to provide, in conjunction with the ion-paired nucleophile, a high level of organization in which the addition could occur, leading to high enantioselectivity. Control experiments showed that the basic amine was required to obtain high *ee*, as well as having the correct chain length, all suggesting a high degree of organization is occurring with the successful catalyst (Figure 2d). The same authors demonstrated that this ligand and related variants could be effective in a range of reactions in which the substrates were systematically varied; silver could also be used as the metal in place of gold.^2a,10^ A subsequent investigation from Togni and Pastor delved deeply into the mechanism (Figure 2e). This study suggested that the aldehyde does not complex with the gold, but that both the binding of the isocyano substituent of the enolate and the ionic interaction with the ammonium side chain effectively block one face of the enolate, leaving the other face open for attack (Figure 2e).^11^

#### 2.1.2 Palladium

**Figure 3:**
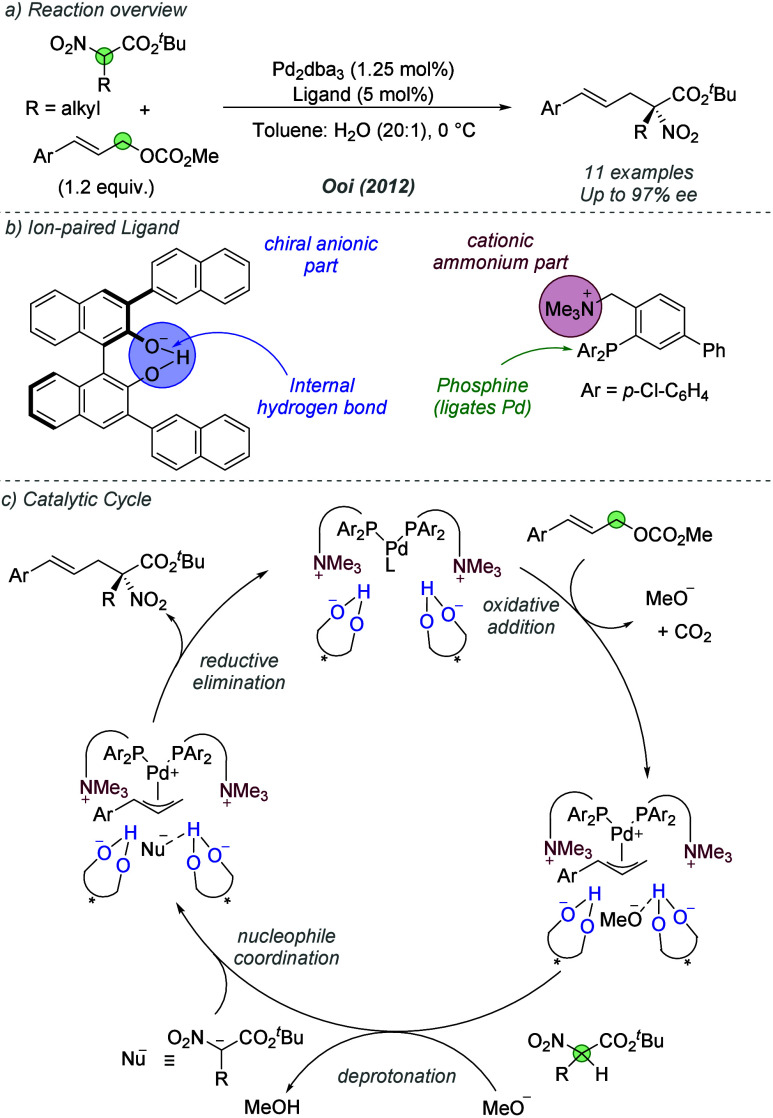
Enantioselective allylic alkylation of α-nitrocarboxylates using a cationic phosphine ligand paired with a chiral anion.

Enantioselective transition metal catalysis typically relies on chiral information incorporated directly into the ligand scaffold. Ooi and co-workers have carried out pioneering studies into an alternative approach, whereby the chirality is located on an associated chiral anion that is ion-paired to an achiral cationic ligand.^8c^ In 2012, they reported an enantioselective Pd-catalyzed allylic alkylation of α-nitrocarboxylate nucleophiles (Figure 3a).^12^ The ligand consisted of an achiral phosphine bearing a cationic quaternary ammonium group, which was paired with a chiral binaphtholate anion featuring an internal hydrogen bond (Figure 3b). A catalytic cycle was proposed based on kinetic data and an observed nonlinear relationship between the enantioenrichment of the ligand and the *ee* of the product (Figure 3c). The active Pd(0) catalyst, bearing two ion-paired phosphines, undergoes oxidative addition into the allylic C–O bond to form an allylpalladium intermediate and a methyl carbonate anion, which can decarboxylate to eliminate CO_2_ and generate a methoxy anion. The latter deprotonates the α-nitrocarboxylate to generate a nucleophilic, prochiral nitronate. Enantioinduction is thought to be achieved in the final reductive elimination, where a hydrogen bonding interaction with one of the chiral anions is proposed to enable discrimination between enantiotopic faces of the nitronate nucleophile. Various ligands were evaluated in which the ammonium group was located at different positions of the phosphine and with different linkers. However, deviation from the optimal ligand design (shown in Figure 3b) was found to be highly detrimental to enantioselectivity, highlighting the importance of the correct placement of the associated anions with respect to the palladium complex. This was a landmark study, as it demonstrated the potential of an outer sphere chiral counterion strategy for asymmetric induction.

**Figure 4:**
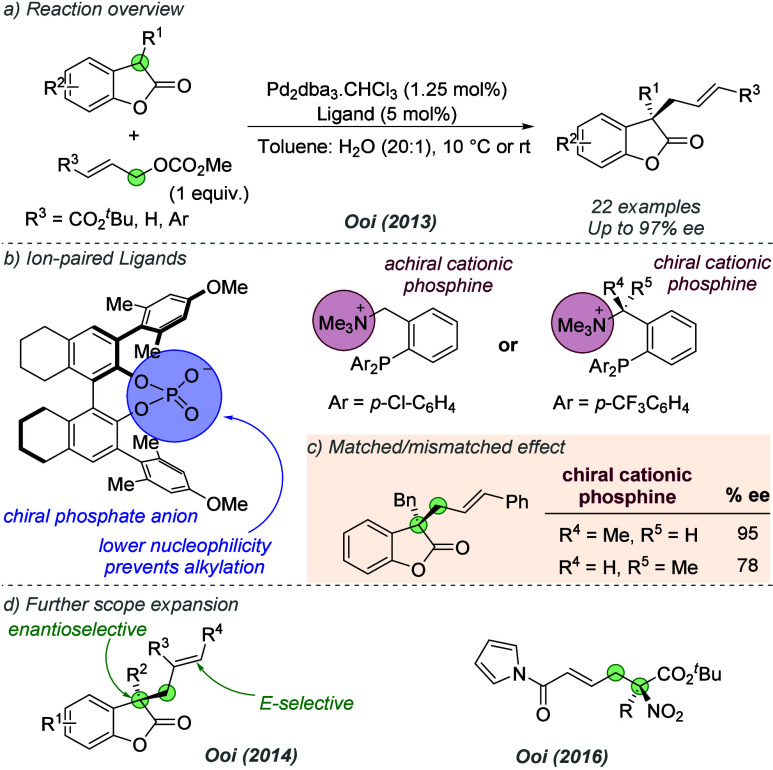
Enantioselective allylic alkylation of benzofuran-2(3*H*)-ones using phosphate-paired cationic phosphines.

The following year, Ooi and co-workers further extended this work to the enantioselective allylic alkylation of benzofuran-2(3*H*)-ones (Figure 4a).^13^ Their initial experiments used a reactive allylic methyl carbonate bearing an ester on the alkene (R^3^=CO_2_*t*Bu), but the authors found that the first generation binaphtholate anion used in the previous report underwent *O*-alkylation under the reaction conditions, impeding its stereocontrolling ability. Alternative chiral anions of lower nucleophilicity were therefore investigated, with optimal results being achieved using a chiral phosphate (Figure 4b). Pairing this species with the same achiral cation used previously gave excellent results for several substrate combinations (specifically, those using an allylic carbonate with R^3^ = CO_2_*t*Bu). For less reactive electrophiles such as cinnamyl carbonates, the enantioselectivity was improved by using the chiral anion in conjunction with a cationic phosphine which featured a defined stereocenter on the linker between the ammonium and the aromatic ring of the phosphine (Figure 4c). A matched/mismatched effect was observed depending on which enantiomer of the phosphine was used, demonstrating the contribution of both components. On the optimization substrate, the matched combination afforded the product in 95% *ee* compared to 78% *ee* for the mismatched and the scope of substrates was explored. Subsequent reports from the same group have focused on expansion of the ligand system. In 2014, Ooi and co-workers identified a related ion-paired catalyst for the control of both enantioselectivity and *E*/*Z*-selectivity in the product resulting from the allylic alkylation of benzofuran-2(3*H*)-ones with 1,2-disubstituted allylic carbonates, demonstrating that selectivity control is not limited to enantiocontrol in these systems (Figure 4d).^14^ An allylic alkylation of α-nitrocarboxylates with amide-containing electrophiles was reported in 2016, building on the results of the 2012 report, but this used a chiral phosphate instead of a binaphtholate ion.^15^

**Figure 5:**
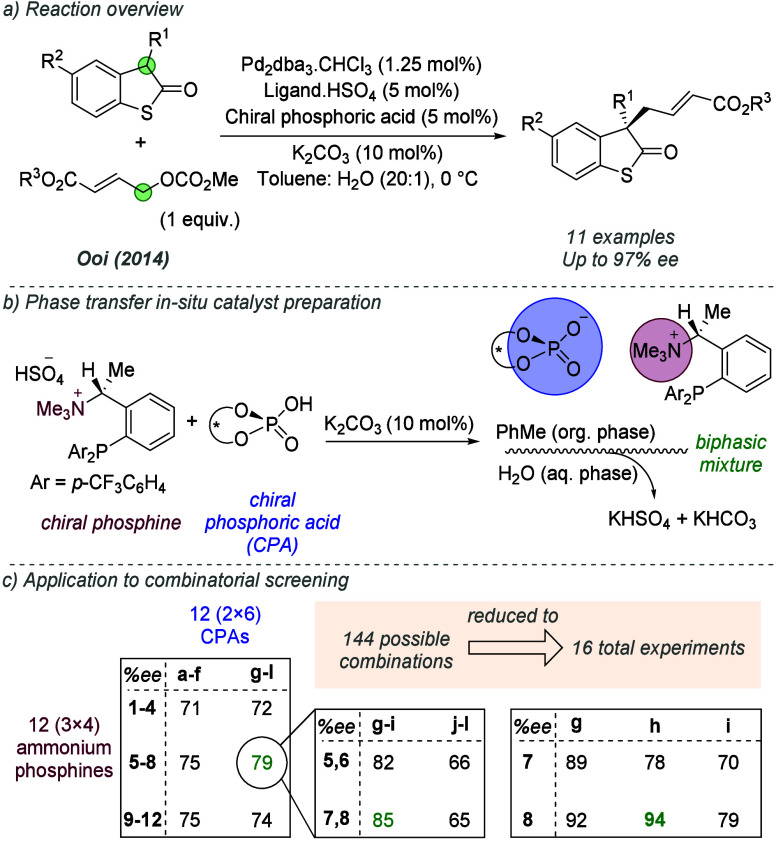
Enantioselective allylic alkylation of benzo[b]thiophen-2(3*H*)-ones and combinatorial reaction optimization.

In 2014, Ooi and co-workers reported an enantioselective allylic alkylation of benzo[*b*]thiophen-2(3*H*)-ones (Figure 5a).^16^ In this work, the authors described a method to speed up the evaluation of ion-paired ligands; departing from the preformation of the discrete ion pair, as in the earlier reports, the relevant cation and anion were introduced separately and the ion-paired catalyst formed *in situ* (Figure 5b). The cationic phosphine was introduced with a hydrophilic bisulfate anion and the chiral anion was introduced as a chiral phosphoric acid (CPA), with the charged species formed following deprotonation by catalytic K_2_CO_3_. The liquid–liquid biphasic reaction conditions enabled the resulting hydrophilic components to occupy the aqueous phase, leaving the desired ion-paired catalyst in the organic phase. This approach enabled an innovative combinatorial strategy for ligand optimization (Figure 5c). The authors sought the most effective ion-paired catalyst from 12 ammonium phosphines and 12 CPAs (144 possible combinations). Rather than performing 144 separate experiments, the ammonium phosphines were divided into three bands (3 × 4), and the CPAs into two bands (2 × 6). The components in each band were combined, and the bands screened together to form a 3 × 2 matrix. The best result (79% *ee*) was carried forward, split into further bands, and the process repeated, until the most effective combination (8h) was identified in just 16 total experiments. The approach was validated by the individual testing of all 144 combinations, which confirmed 8h as optimal. Following this, a scope of various benzo[*b*]thiophen-2(3*H*)-ones was demonstrated with the reactions proceeding in excellent yield and enantioselectivity.

**Figure 6:**
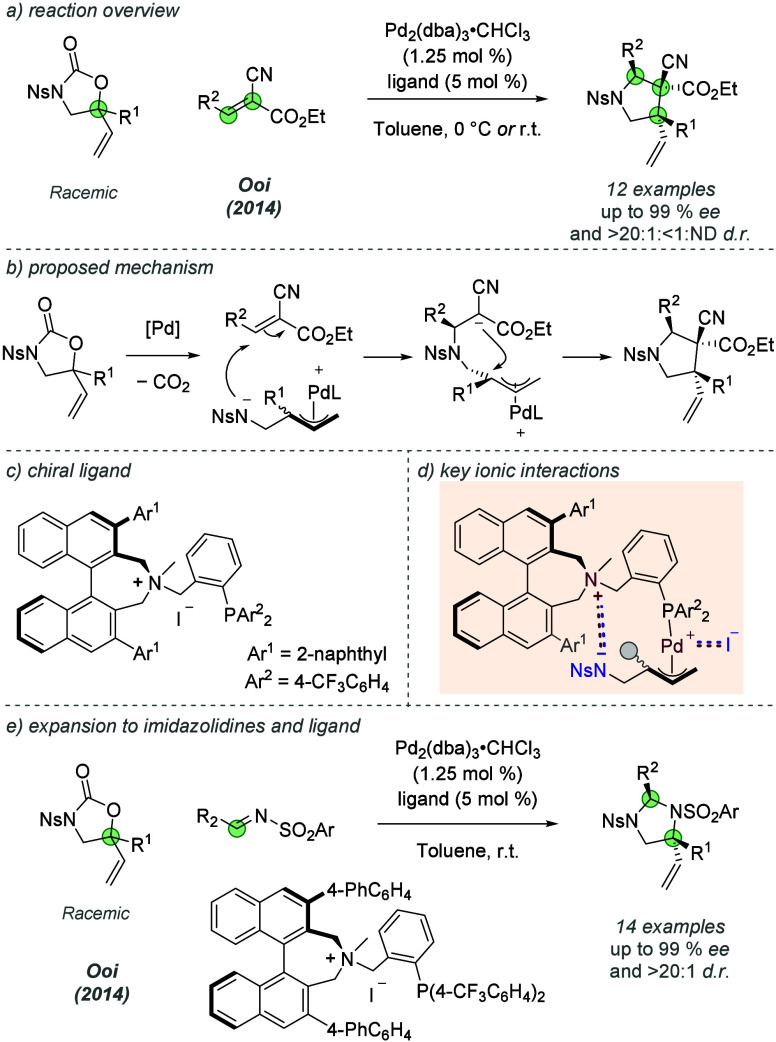
Enantioselective [3 + 2] annulation reactions to access enantioenriched five-membered heterocycles using a phosphine ligand incorporating a chiral ammonium group.

In 2014, Ooi and co-workers built on these earlier studies to incorporate a chiral ammonium group covalently into the phosphine ligand scaffold. They utilized this chiral ammonium phosphine to enable a highly enantio- and diastereoselective [3 + 2] annulation of 5-vinyloxazolidinones and activated trisubstituted alkenes to access densely substituted pyrrolidines in excellent yields (Figure 6a).^17^ After palladium coordination to the 5-vinyloxazolidinone and extrusion of CO_2_, the reaction is thought to proceed through amide attack on the electron-deficient alkene followed by ring closure at the electrophilic allylpalladium species (Figure 6b). The authors proposed that the chiral ammonium group on the ligand engages in a crucial ionic interaction with the remote amide anion while still bound to the allylpalladium complex through the aromatic phosphine (Figure 6d). Use of iodide as the ligand counteranion proved crucial to obtaining high enantioselectivity: the iodide anion is thought to ion-pair with the positively charged palladium, thus releasing the amide anion to engage in ion-pairing with the chiral ammonium cation, allowing formation of a highly organized system. Remarkable stereocontrol is achieved through discrimination of prochiral faces of the trisubstituted alkene (and resulting carbanion) and control of planar chirality of the π-allylpalladium electrophile. The authors established that the ligand can control planar chirality of the intermediate through isomerization and the use of trisubstituted alkenes which had three different substituents allowed formation of contiguous all-carbon stereocenters with excellent control, further highlighting the power of their ion-paired strategy. Ooi and co-workers subsequently expanded this reaction to enable the synthesis of enantioenriched imidazolidines, by exchanging the trisubstituted alkene for an *N*-sulfonyl imine coupling partner (Figure 6e).^18^ A closely related ligand was capable of recognizing the imine and resulting anion, and the imidazolidine products were afforded in high yields as well as high enantio- and diastereoselectivities with a range of substitution on both partners.

#### 2.1.3 Nickel

**Figure 7:**
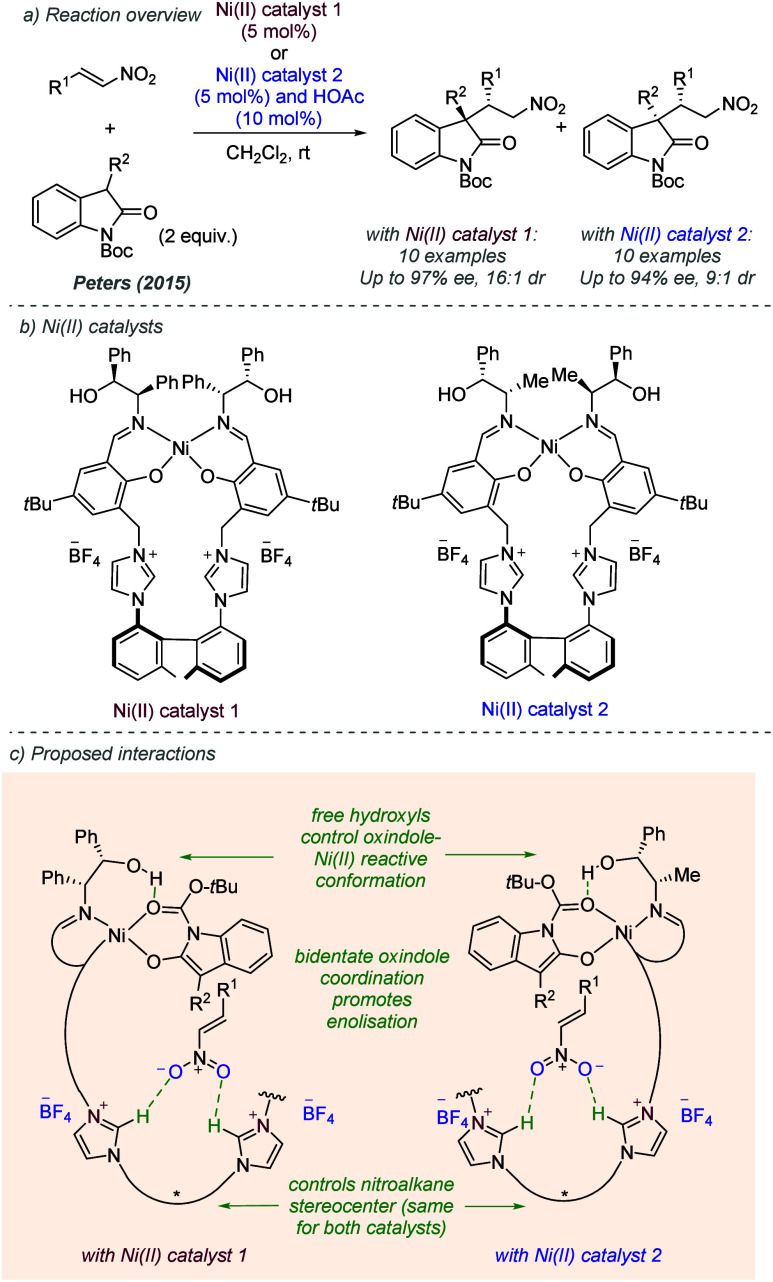
Diastereodivergent and enantioselective conjugate addition of oxindoles to nitroolefins with polyfunctional Ni(II) catalysts.

Peters and co-workers have made substantial contributions to the area of enantioselective Lewis acid-catalyzed reactions by utilizing carefully designed ligands that contain cationic groups at remote positions. Their earliest work was using Al complexes, which are outside the scope of this review, but more recently have used transition metals to great effect.^19^ In 2015, Mechler and Peters reported a polyfunctional Nickel bis(phenoxyimine) complex possessing alcohol hydrogen bond donors as well as cationic imidazolium units, which was able to catalyze the 1,4-addition of oxindoles to nitroolefins with high enantioselectivity (Figure 7a).^20^ Both diastereomers of the 1,4-addition products were accessible, depending on the choice of catalyst. The catalysts contained a Ni(II) metal center bound to bis(phenoxyimine) units, cationic imidazoliums bound to an axially chiral biaryl axis and alcohol side chains with two contiguous stereocenters (Figure 7b). During the reaction, it was proposed that the oxindole would undergo bidentate coordination to the nickel(II) center, promoting enolization to form the active nucleophile, while hydrogen bonding interactions between the catalyst and carbonyl of the Boc group of the oxindole would precisely orientate the latter (Figure 7c, only one iminoalcohol shown in each complex for clarity). It was proposed that the axial chirality of the biaryl moiety would exert enantiocontrol over the stereocenter originating from the nitroolefin electrophile through a combination of hydrogen bonding and electrostatic interactions involving the cationic units. Consistent with this hypothesis, the absolute configuration of the chiral axis was the same for both catalysts, and so was the configuration of the stereocenter originating from the nitroalkene electrophile in both series of diastereomeric products.

A structurally related but dinuclear cobalt complex was later reported for a 1,4-addition reaction using maleimides as electrophiles.^21^

#### 2.1.4 Copper

**Figure 8:**
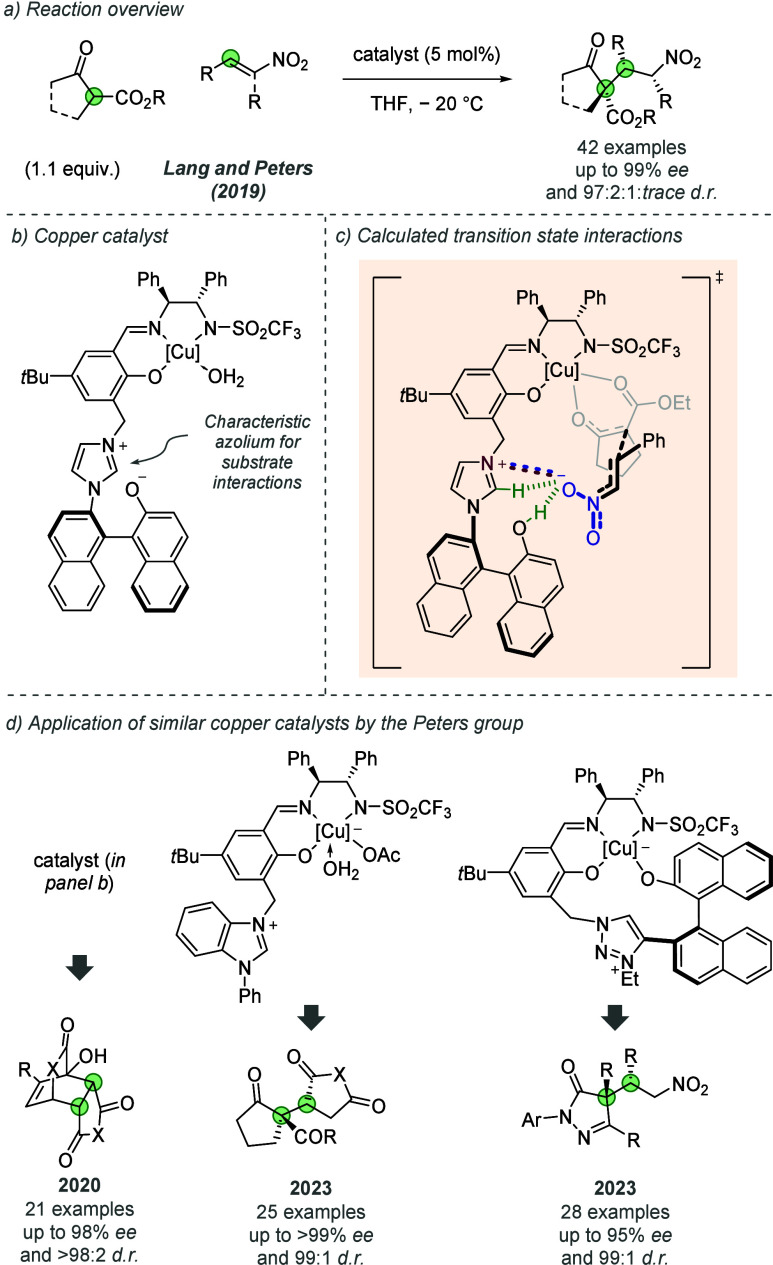
Polyfunctional ligand scaffolds used in a variety of copper-catalyzed reactions.

Continuing their Lewis acid catalysis development using charged polyfunctional ligands for metals, in 2019 Peters, Lang and co-workers reported a chiral copper complex which enabled exceptional control in the 1,4-addition of various 1,3-dicarbonyls to β-substituted nitroolefins (Figure 8a).^22^ Essential to the activity of the catalyst were the chiral salen-type backbone, cationic imidazolium heterocycle and axially chiral aryloxide unit (Figure 8b). The authors demonstrated a broad scope of pronucleophiles and acceptors, which also included excellent diastereocontrol with α,β-disubstituted nitroolefins. This allowed control over three contiguous stereocenters in asymmetric 1,4-additions of this type, a challenging feat to achieve. Furthermore, in all cases the powerful stereocontrol provided by the catalyst enabled the formation of otherwise disfavored diastereomers, which would be difficult to access by other means. Detailed mechanistic investigations, through spectroscopic and DFT studies, revealed a host of important noncovalent interactions during the C–C bond-forming transition state (Figure 8c). With the deprotonated dicarbonyl bound to the copper center, it was shown that the cationic imidazolium and phenol groups activate the nitroolefin and effectively position it to access the observed configuration in high selectivity. Although hydrogen-bonds (green dashed lines) between the nitroolefin and the imidazolium C(2)-H bond are vital for stereocontrol, the authors carried out a number of catalyst modification experiments that suggest that an ionic interaction between the cationic imidazolium and substrate is also important to controlling selectivity.

Since this report, Peters and co-workers have demonstrated several other examples showing the broad capabilities of related copper complexes (Figure 8d). The precise cluster of noncovalent interactions (calculated or proposed) varies for each transformation. However, the cationic azolium motif on the ligand is central in all cases to enabling high selectivity, emphasizing the importance of ionic interactions in these processes. In 2020, the original catalyst, depicted in Figure 8b, was applied to [4 + 2] cycloadditions (Figure 8d, left). Once again, the catalyst was proposed to engage with the substrates in several ways, including Lewis acid activation of the prodiene, followed by deprotonation by the imidazolium aryloxide unit of the catalyst, as well as activation of the dienophile through hydrogen bond donation.^23^ In 2023, the authors reported the application of a related catalyst without the axially chiral aryloxide in the 1,4-addition of 1,3-dicarbonyl nucleophiles to Michael acceptors (Figure 8d, middle).^24^ Several cyclic electrophiles were tolerated, including maleimides and malic anhydride, as well as acyclic examples, such as the nitroolefin electrophile class of the earlier report. The same year, the authors reported a new Cu(II)-1,2,3-triazolium-aryloxide catalyst and its application to the addition of pyrazolones to nitroolefins (Figure 8d, right).^25^

### 2.2 Ligand Bears an Anionic Group

#### 2.2.1 Palladium

**Figure 9:**
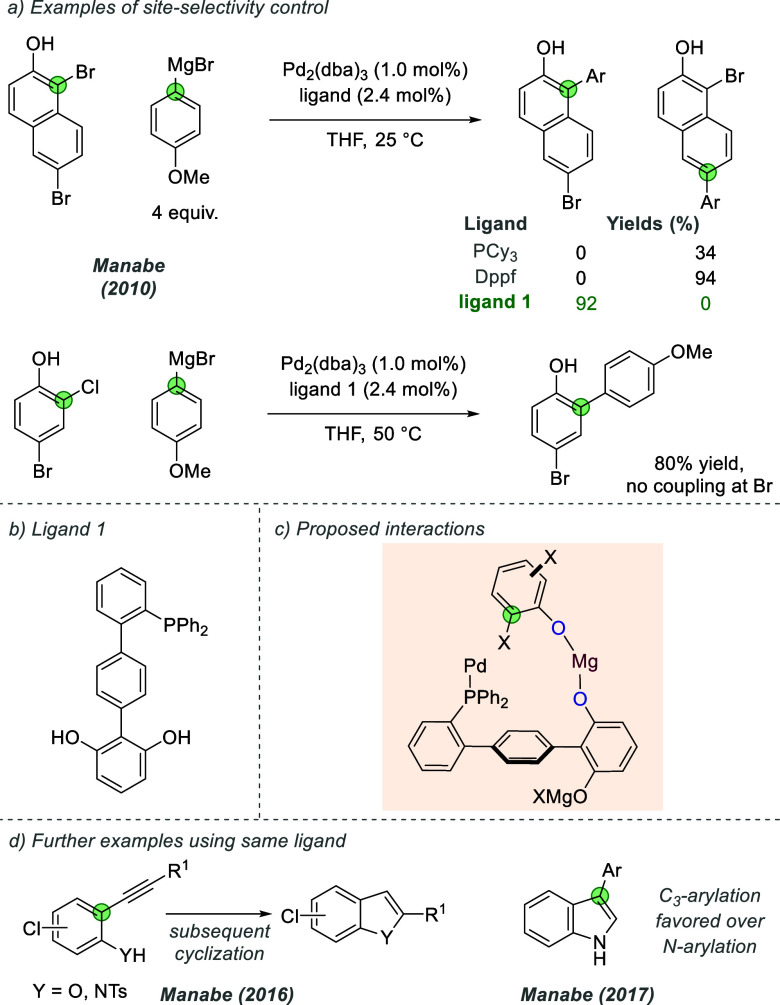
Site-selective cross-coupling of dihaloarenes with arylmagnesium bromides.

In the case of ligand scaffolds for palladium that have been modified to bear an anionic group, those belonging to the phosphine family possessing a 2-biaryl substituent have been investigated to tackle both site- and enantioselectivity challenges. The Manabe group made important early advances in this area with several extended bifunctional ligand scaffolds reported since 2007.^26^ In these reports, aryloxide-metal bonds are depicted, involving coordination rather than ionic interactions, as presumably the bonds involved will have significant covalent character. Although these examples are not proposed to be ion-pairing, it is interesting to survey this work as an example of an elegant design principle for selectivity control which could be extended to ion-pairing with modification of the partners involved (see below). In a representative example, in 2010 Manabe and co-workers reported the development of an extended dihydroxyphosphine scaffold which imparted site-selectivity control in the palladium-catalyzed cross-coupling of dihalophenols with Grignard reagents.^27^ Cross-coupling examples with 1,6-dibromonaphthalen-2-ol or 4-bromo-2-chlorophenol demonstrated the switch in selectivity toward the *ortho* halide when using the functionalized ligand compared to standard phosphine ligands (Figure 9a and b). The authors proposed that a bridging magnesium metal complexed by the ligand and substrate-based phenolates appropriately positioned the *ortho* halide for oxidative addition (Figure 9c). Since that report, Manabe and co-workers have continued to expand their family of hydroxyphosphine ligands and have applied these to numerous palladium-catalyzed reactions, including *ortho*-selective Sonogashira cross-couplings and subsequent cyclization to form the corresponding indole or benzofuran (Figure 9d),^28^ and the C3-selective arylation of indoles.^29^ In these cases, lithium salts of phenols and indoles are formed, which are proposed to aggregate together to direct the oxidative addition process.

**Figure 10:**
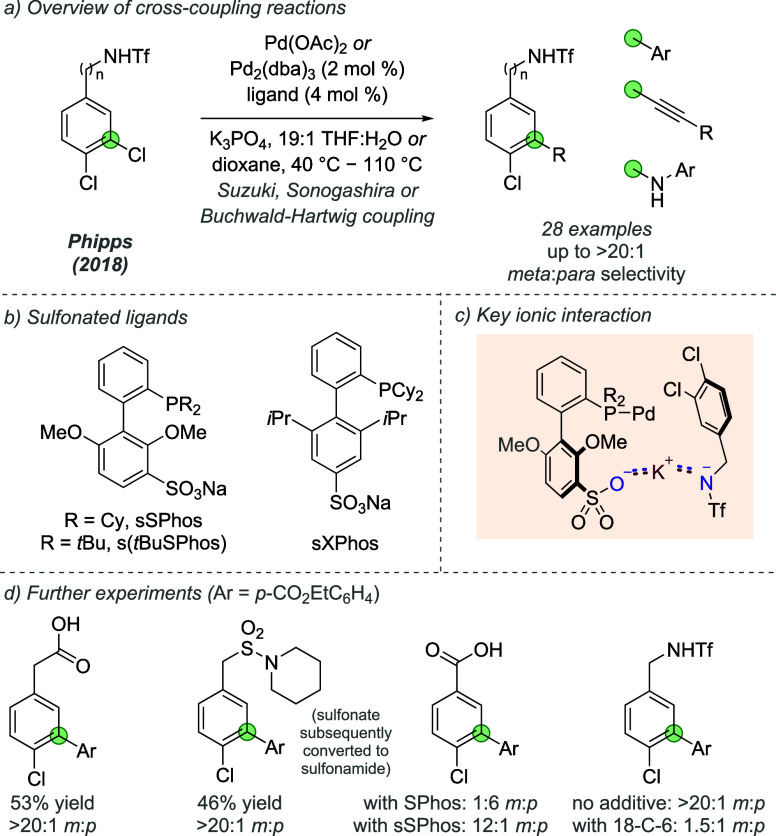
Site-selective cross-coupling of dichloroarenes utilizing sulfonated phosphine ligands, directed by electrostatic interactions.

In 2018, Phipps and co-workers demonstrated the site-selective cross-coupling of dichloroarenes guided by ionic interactions between the substrate and an anionic phosphine ligand for palladium (Figure 10a).^30^ sSPhos and s(*t*BuSPhos), sulfonated versions of SPhos and *t*BuSPhos respectively (Figure 10b), were found to direct several palladium-catalyzed cross-coupling reactions toward the chloride at the *meta* position in the presence of a sterically and electronically similar chloride at the *para* position. The authors suggested that, under basic conditions, the potassium cation associated with the deprotonated triflamide substrate engages in an electrostatic interaction with the anionic sulfonate group of the ligand (Figure 10c). Use of other Bro̷nsted acidic substrates that produce anionic carboxylate or sulfonate groups on deprotonation led to similarly high *meta*-selective Suzuki cross-couplings, providing further support for this hypothesis (Figure 10d, left). Additionally, sSPhos could override the innate *para*-selectivity seen with standard SPhos in the Suzuki coupling of 3,4-dichlorobenzoic acid (Figure 10d, center right). Experiments involving the addition of crown ethers of a suitable size to sequester the alkali metal cation reduced site selectivity, providing further support for the importance of ionic interactions for the observed selectivity (for example, see Figure 10d, right).

**Figure 11:**
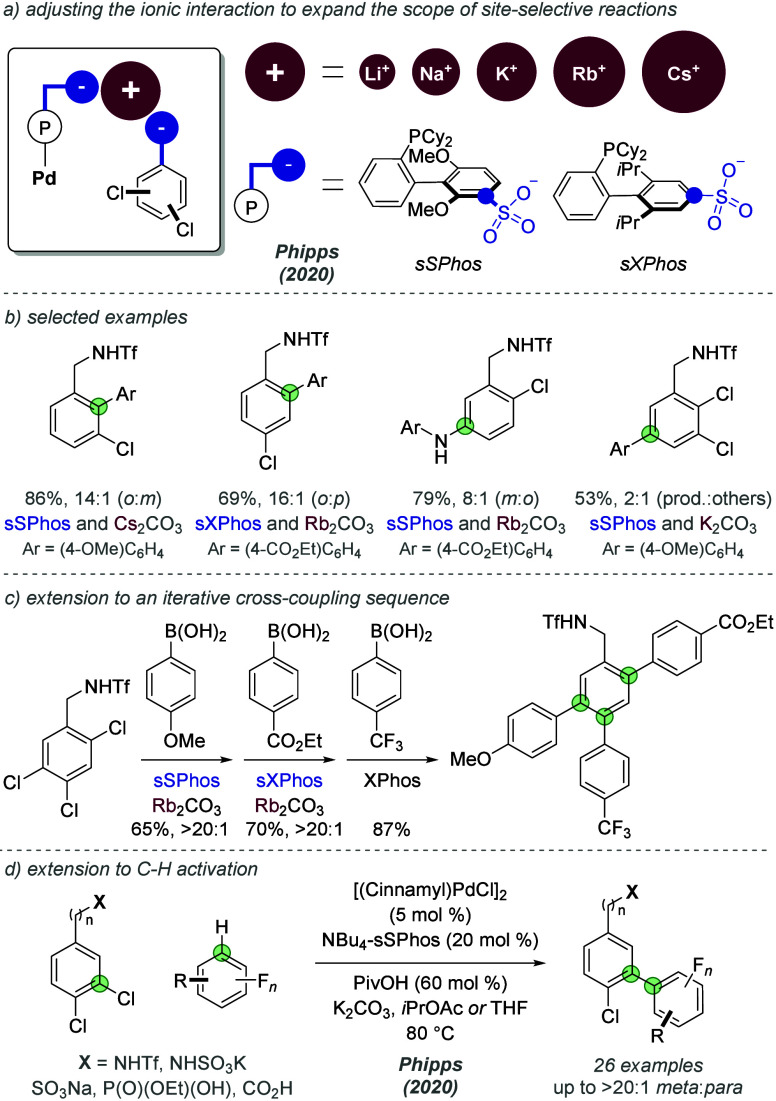
Extension of site-selective cross coupling to various dichlorinated and trichlorinated substitution patterns using electrostatically directed palladium catalysis.

In 2020, the same authors used this strategy to tackle the challenge of selective cross-coupling reactions of more diverse dichloroarene substitution patterns as well trichloroarenes, which would otherwise give intractable mixtures of products with standard ligands.^31^ They hypothesized that if the alkali metal cation was playing a key role in the selectivity-determining transition state, then varying its size may lead to tuning of site-selectivity during the oxidative addition step (Figure 11a). A “toolkit” was used which comprised of two ligands whereby the sulfonate group occupies different positions on the lower ring of the ligand (sSPhos and sXPhos) as well as five stoichiometric bases with various cation sizes. Testing of the toolkit on a given substrate allowed the optimal of the ten possible combinations (5 × 2) to be identified, and this was applied to various substitution patterns in both Suzuki-Miyaura and Buchwald-Hartwig coupling (Figure 11b). Additionally, the authors demonstrated an iterative Suzuki coupling sequence on a trichloroarene to afford a heavily substituted benzene core in high selectivity (Figure 11c). Also in 2020, Phipps and co-workers paired a similar *meta*-selective oxidative addition of dichloroarenes with the C–H activation of (hetero)fluoroarenes (Figure 11d).^32^ This demonstrated that an electrostatically directed selectivity-controlling step was compatible with subsequent CMD-type C–H activation with palladium. The reaction was compatible with several different anionic groups on the dichloroarene substrate, including triflamide, phosphonate and sulfamate, and was proposed to proceed through a similar interaction to previous site-selective cross-couplings. More recently, palladacycles bearing sulfonated phosphine ligands were used to tackle the *meta*-selective cross-coupling of 3,4-dichlorophenol and 3,4-dichlorobenzyl alcohol by Xu, Song, Zhang and co-workers in an analogous strategy.^33^ Ionic interactions between ligand and substrate were suggested to be key factors in determining site-selectivity for phenolic substrates, which were found to undergo deprotonation under the basic reaction conditions.

**Figure 12:**
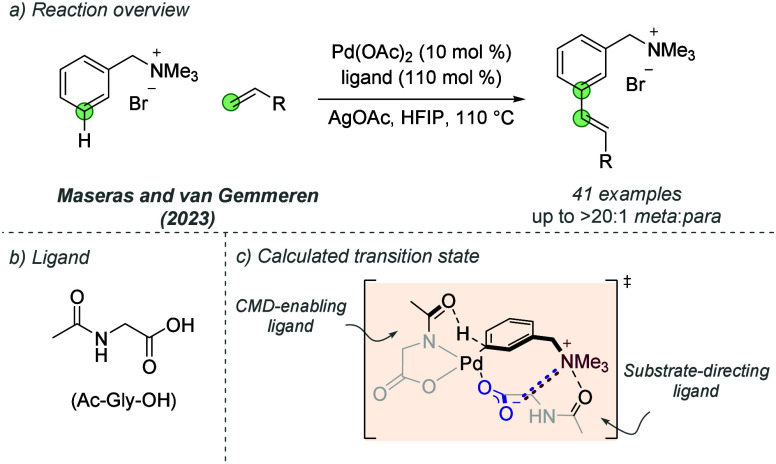
*meta*-Selective C–H olefination of arenes bearing cationic ammonium groups.

In the above work, ionic interactions could be used to control site-selectivity in a C-X oxidative addition process, but it is also intriguing to consider whether they can be used to control the site-selectivity of a C–H activation step. Indeed, there has been considerable interest in controlling the regioselectivity of distal C–H activation of arenes with various strategies.^34^ In the context of palladium-catalysis, an approach that successfully utilized ionic interactions in this way was demonstrated in 2023 by van Gemmeren, Maseras and co-workers in a *meta-*selective C–H olefination of arenes (Figure 12a).^35^ The simple amino acid-derived ligand Ac-Gly-OH employed in this transformation was proposed to have a key bifunctional nature, according to DFT calculations (Figure 12b). One ligand on Pd, bound in a bidentate manner, enables catalytic activity in a CMD-type C–H activation, while the second ligand, bound in a monodentate mode, participates in a combination of attractive noncovalent interactions with the quaternary ammonium directing group on the substrates. This includes an ionic interaction between the carboxylate and ammonium and a cation-dipole interaction between ligand amide and ammonium. This combination of interactions was proposed to effectively position the arene for functionalization at the *meta* position, with a variety of control experiments providing support for this hypothesis (Figure 12c). Although the ammonium moiety was essential for selectivity, the authors showed derivatization and removal of this group to provide multifunctionalized arenes. The authors demonstrated an extensive scope of arenes and electron-poor olefins which afforded functionalized products with excellent *meta*/*para* selectivities.

In addition to applications in which ionic interactions have been used to control site-selectivity in Pd-catalysis, there have been several examples of enantiocontrol in a similar manner. On the typical dialkylbiarylphosphine ligand scaffold,^36^ incorporation of a lower ring substituent *meta* to the biaryl bond (for example, the sulfonate group in sSPhos) breaks the symmetry of the biaryl giving rise to axially chiral ligands. The following examples highlight the recent progress made with anionic phosphine ligands that exhibit chirality in this manner, as applied to asymmetric palladium-catalyzed reactions.

**Figure 13:**
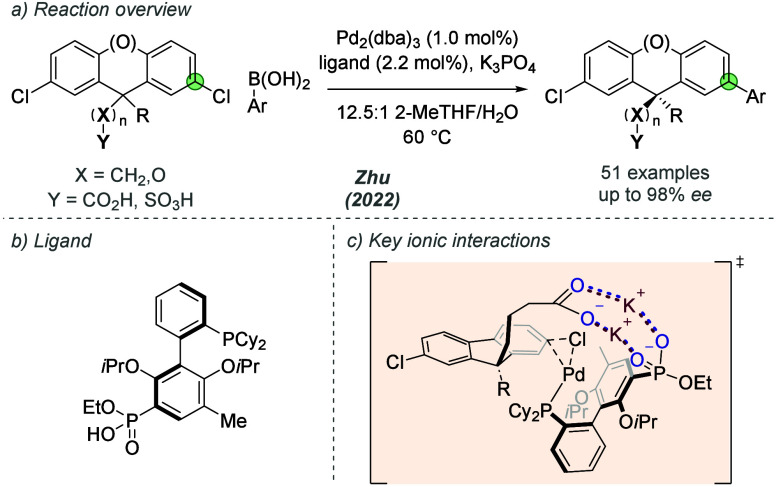
Enantioselective desymmetrizing Suzuki-Miyaura cross-coupling of cyclic bis(chloroaryl)methane derivatives enabled by a phosphonate-functionalized ligand.

In 2022, Zhu and co-workers developed a chiral phosphonate-functionalized ligand for the desymmetrizing Suzuki-Miyaura cross-coupling of cyclic bis(chloroaryl)methane derivatives (Figure 13a,b).^37^ The authors showed that substrates bearing a pendant Bro̷nsted acid group such as a carboxylic acid or sulfonic acid at the central position underwent enantioselective cross-coupling at enantiotopic C–Cl bonds. The authors proposed that key ionic interactions were occurring between the deprotonated substrate groups, the associated potassium cation and the anionic phosphonate on the ligand–a similar interaction to that previously proposed by Phipps and co-workers in related site-selective processes using sSPhos (Figure 13c). This allowed a high degree of organization during the enantiodetermining transition state for oxidative addition which was determined through DFT analysis in their next report (*vide infra*). Experimentally, Zhu and co-workers showed that through either the addition of 18-crown-6 to sequester potassium cations, or with an ester in place of an acid on the substrate, enantioselectivity fell to <20% *ee*. This provided support for the importance of the bridging ionic interaction between substrate and ligand. A thorough evaluation of linker length (labeled (**X**)_n_, Figure 13a) for carboxylic and sulfonic acids also demonstrated the importance of the spatial relationship between the substrate directing-group and reactive C–Cl bond. In many cases the new quaternary stereocenter being formed is a significant distance from the directing functional groups. Excellent levels of enantioselectivity were found for various other aliphatic and aromatic groups on the remote quaternary carbon of the substrate, as well as xanthene-type substrate scaffolds and different boronic acid coupling partners. The enantiopure ligand could be concisely synthesized from RuPhos using a resolution step involving attachment of a chiral auxiliary and chromatographic separation.

**Figure 14:**
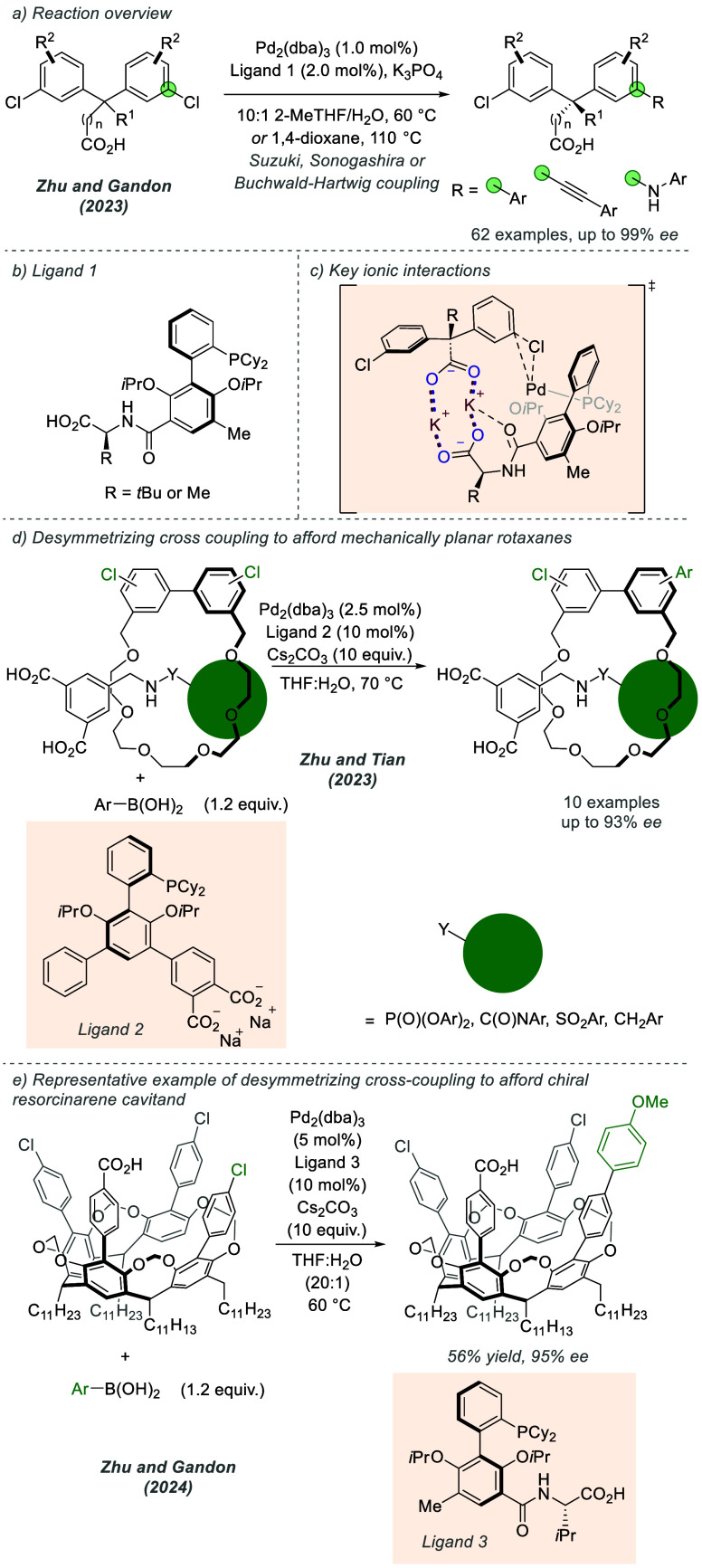
Desymmetrizing cross-coupling to afford acyclic tertiary or quaternary stereocenters using a modified ligand that possesses both axial and point chiral elements, the synthesis of mechanically planar rotaxanes, and chiral resorcinarene cavitands.

Further development of this methodology relating to desymmetrizing cross-coupling reactions was reported by Wei, Gandon and Zhu in 2023 (Figure 14a).^38^ In this instance, desymmetrizing Suzuki-Miyaura, Sonogashira and Buchwald-Hartwig amination reactions were performed on bis(chloroaryl)methanes to afford tertiary or quaternary acyclic stereocenters. The increased flexibility of acyclic substrates (compared to cyclic versions used in the earlier work) necessitated the development of a family of second-generation ligands to address the more challenging stereocontrol. The authors found that swapping the phosphonate group for amino acid-derived carboxylic acids enabled excellent enantioselectivities across the range of cross-coupling reactions investigated (Figure 14b). The effect of the amino acid substituent and stereochemistry was probed, and detailed computational investigations into the nature of the long-range stereocontrol provided insight into the key substrate–ligand interactions (Figure 14c). Analogous to the interactions shown in Figure 13c, a bridging ionic interaction between the substrate carboxylate and ligand carboxylate was proposed. Importantly, an additional interaction between the amide carbonyl and a bridging potassium cation, not possible with the first-generation ligand, was proposed to contribute to the effective stereocontrol achieved with the second-generation ligand family. Zhu, Tian, and co-workers also employed distal ionic interactions in desymmetrizing Suzuki-Miyaura cross-couplings to access mechanically planar chiral rotaxanes (Figure 14d).^39^ The ligand structure was once again optimized to obtain high enantioselectivity, this time bearing a pair of carboxylates separated from the main ligand scaffold by an aryl linker. Most recently, Zhu, Gandon and co-workers applied their approach to establishing chirality in resorcinarene cavitands, using a closely related ligand to that reported in their earlier work (Figure 14e).^40^

**Figure 15:**
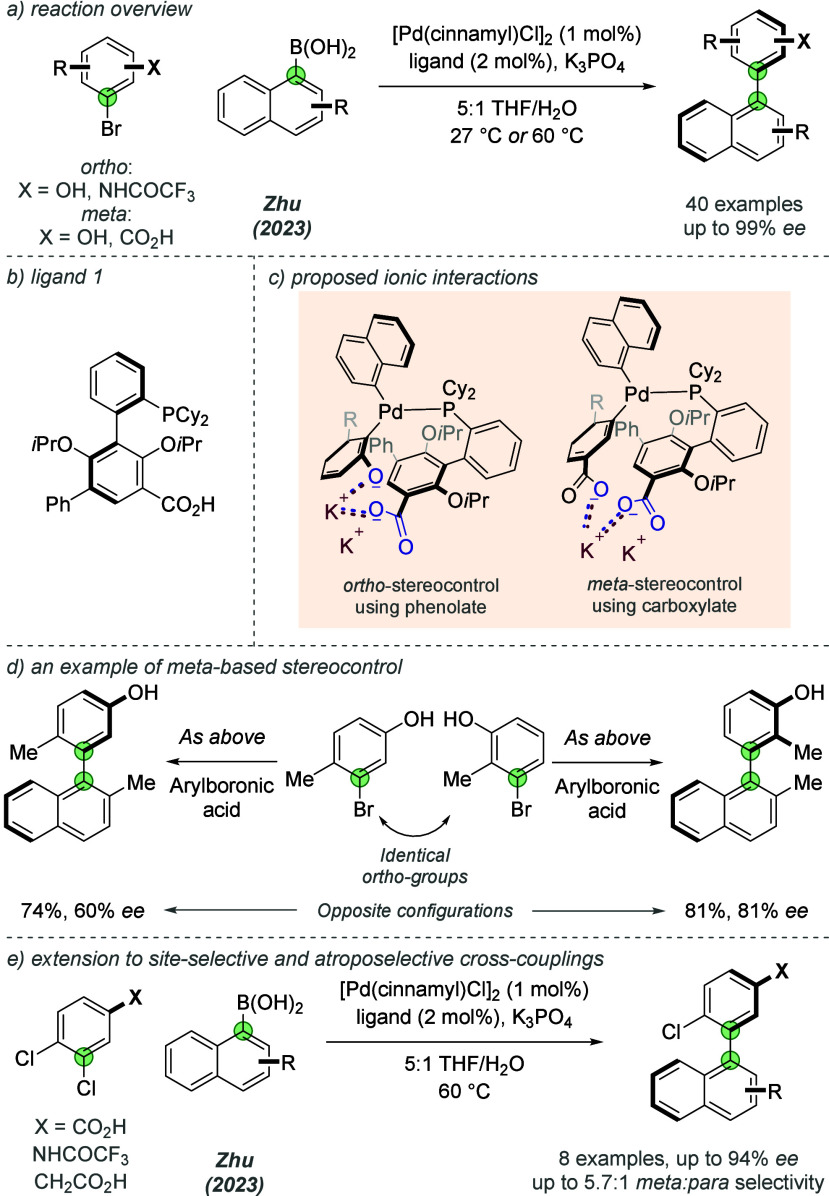
Atroposelective Suzuki-Miyaura cross-couplings able to use a *meta*-located directing group.

In addition to their work on enantioselective desymmetrizing reactions, Zhu and co-workers have applied their ligand family to highly atroposelective Suzuki-Miyaura cross-couplings (Figure 15a).^41^ In this study, the authors proposed ionic substrate–ligand interactions utilizing directing groups (labeled **X**) located at either the *ortho*- or the *meta*-positions of the aryl halide. In the latter case especially, this is unusual, since long-range catalyst direction is required. The ligand (Figure 15b), containing a substrate-binding carboxylic acid group, was found to effectively direct the atroposelective cross-coupling of aryl halides and naphthyl boronic acids to give a diverse collection of substituted biaryl products. In all cases an arene substituent, which could plausibly be deprotonated under the reaction conditions at either the *ortho* or *meta* position, was required to obtained high *ee*, and the authors provided evidence which supported deprotonation. The authors demonstrated successful reactions with *ortho*-based hydroxyl or (trifluoroacetyl)amino directing groups and with *meta*-based hydroxyl or carboxylic acid groups. Several examples highlighted how *meta* directing groups can act as the dominant stereocontrolling element and afford similarly configured enantioenriched products by overriding *ortho*-substitution effects (Figure 15d). The authors proposed ionic interactions between the potassium cation of the deprotonated substrate and anionic ligand, analogous to the authors’ previous studies and the prior site-selective work of Phipps and co-workers. The tolerance of the directing group to either *ortho*- or *meta*-substitution can be attributed to the low directionality and long-range characteristics of ionic interactions–a reminder that low directionality is not necessarily a detrimental feature. In a later report, Zhu and co-workers demonstrated how the ligand is able to control site-selectivity as well as atroposelectivity in the *meta*-selective Suzuki-Miyaura cross-couplings between dichloroarenes and naphthyl boronic acids (Figure 15e).^42^

**Figure 16:**
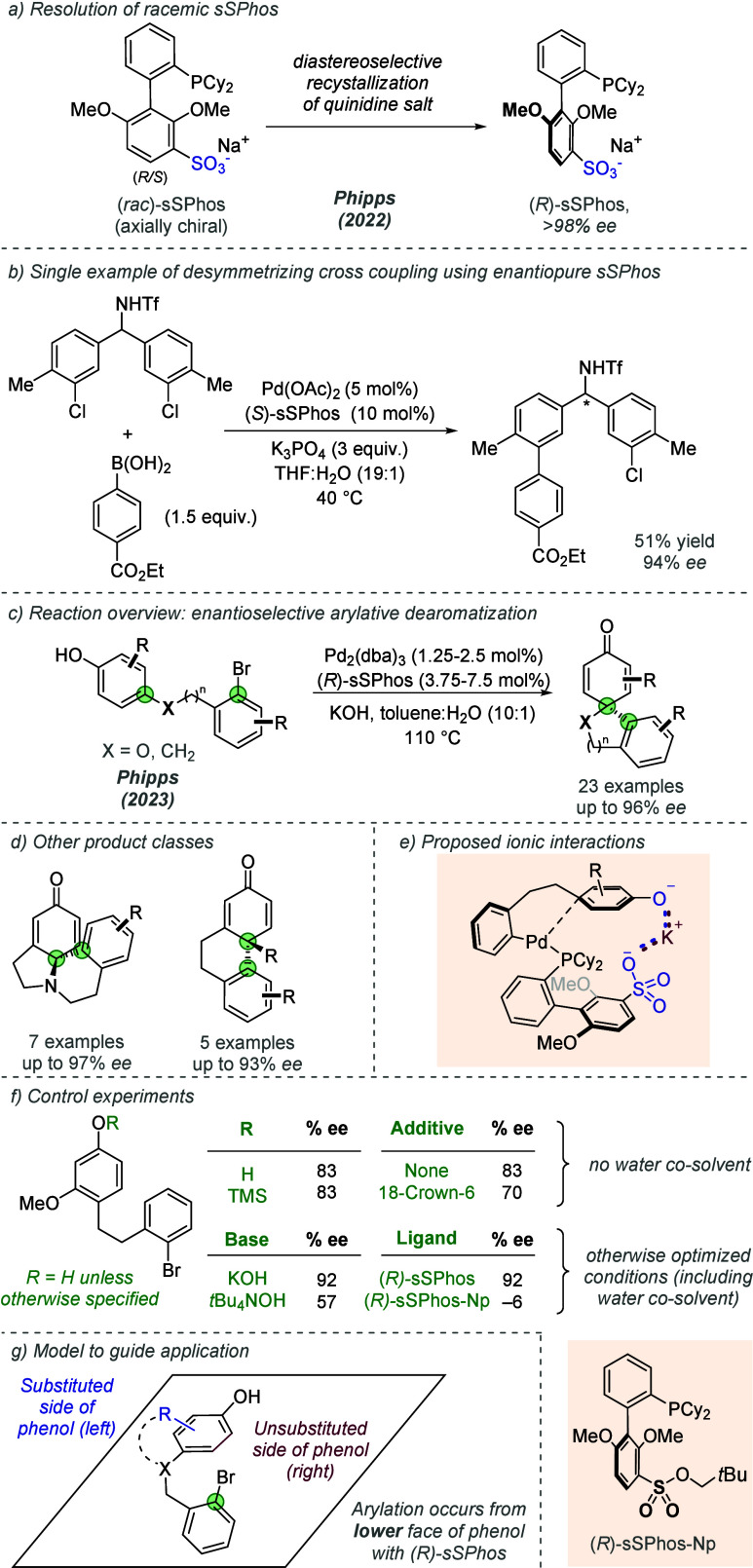
Use of (*R*)-sSPhos as a general chiral ligand for enantioselective intramolecular phenol dearomatization reactions, proposed to operate through ionic interactions.

Since their original reports on using (*rac*)-sSPhos to direct site-selective cross-couplings through electrostatically directed catalysis (Figure 11), Phipps and co-workers have also utilized enantiopure (*R*)-sSPhos to control asymmetry in palladium catalysis. This was first shown in 2022 in the context of atroposelective Suzuki-Miyaura cross-couplings of phenolic partners to form axially chiral 2,2′-biphenols.^43^ This reaction was proposed to proceed through hydrogen-bonding interactions between the phenolic coupling partners and the anionic sulfonated ligand, therefore falling outside the scope of this Review. In this work, the authors demonstrated that (*R*)-sSPhos could be readily obtained in high enantiopurity after recrystallization of its quinidine salt in a chiral resolution process (Figure 16a). The enantiopure ligand was also applied to a single desymmetrizing cross-coupling example, in which a bridging ionic interaction was proposed to enable enantiodetermining oxidative addition, in analogy with the previous site-selective work (Figure 16b**)**. (*R*)-sSPhos has recently been used by the same group as a general chiral ligand for a enantioselective intramolecular phenol dearomatization reactions to form a range of scaffolds (Figure 16c).^44^ (*R*)-sSPhos could be applied to four distinct substrate classes, two of which had been previously rendered enantioselective using bespoke chiral phosphine ligands, affording complex enantioenriched cyclohexanone scaffolds bearing a quaternary stereocenter (Figure 16c and d). The scope included substitution on the phenol, aryl halide rings, and variation of the alkyl linker length (Figure 16). Although the authors proposed hydrogen bonding interactions were at play in the Suzuki coupling to form biphenols, here with a stronger base it is likely that a phenolate forms and an ionic interaction was proposed in analogy with the authors’ earlier work on site-selective cross coupling (Figure 16e). A TMS-protected substrate, which revealed the phenolate *in situ* without an additional proton source, afforded the product in high enantioselectivity, suggesting that hydrogen bonding was not occurring (Figure 16f). The inclusion of 18-crown-6 led to a reduction in enantioselectivity, as did replacement of potassium with tetrabutylammonium. These findings pointed toward an ionic interaction involving a bridging potassium cation between a deprotonated phenol and the anionic ligand-based sulfonate. A control experiment in which the sulfonate group of the ligand was converted to a sulfonate ester gave minimal enantioselectivity, suggesting that attractive interactions are important and the sulfonate is not simply acting as a bulky steric group to exert stereocontrol. Despite the structural differences between the substrate classes, their absolute configuration was consistent with arylation occurring from the lower face of the phenol with (*R*)-sSPhos when the substituent on the phenol was positioned on the left (Figure 16g).

#### 2.2.2 Copper

**Figure 17:**
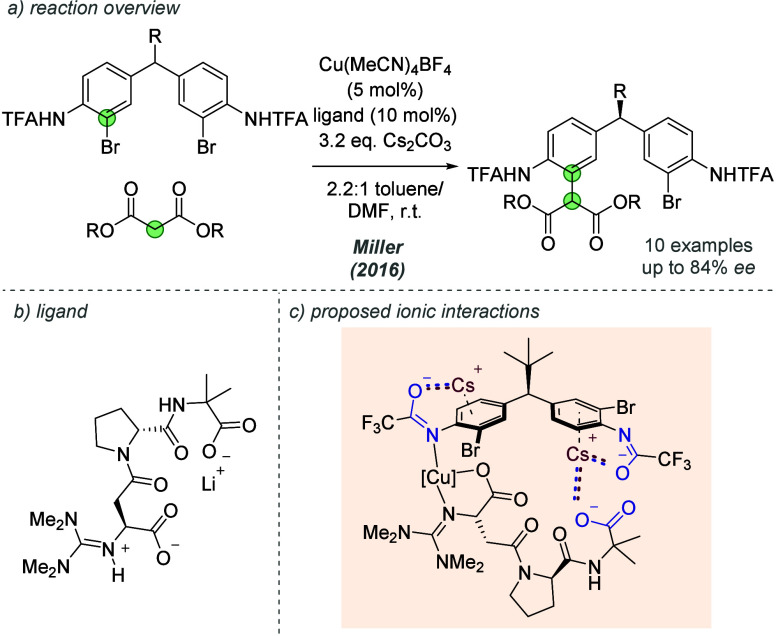
Desymmetrizing Ullman cross-coupling enabled by several noncovalent interactions acting in tandem.

Controlling the chirality at stereogenic centers distal from the site of reactivity is challenging because suitable matching between catalyst and substrate is required over longer ranges. Miller and co-workers previously demonstrated a peptide catalyst capable of the remote desymmetrizing acylation of a bis(phenol), forming a stereocenter in the middle of the substrate (not shown).^45^ They then sought to use similar peptides to control enantioselectivity in a related desymmetrizing Ullman coupling between structurally related bis-aryl bromides and malonates using copper catalysis (Figure 17a).^46^ Essential to success of the modified peptidic ligand (Figure 17b) were the terminal guanidine group for copper-ligation and the *C*-terminal carboxylate. Through mechanistic investigations, the authors proposed that several noncovalent interactions were occurring between this terminal carboxylate and the distal nonreacting arene of the substrate, allowing careful orientation during the reaction (Figure 17c). The trifluoroacetamide group (presumed to be deprotonated *in situ*) enables arene association with a cesium cation, which can also engage in an ionic interaction with the *C*-terminal carboxylate on the ligand. The arrangement and length of the peptide chain ensures that while one arene is engaged with the carboxylate, the other arene is positioned to undergo functionalization with the ligated copper. Kinetic resolution studies provided support for this hypothesis involving abridging cesium cation and removal of the distal arene gave very poor results, providing further evidence for the proposed interaction. High levels of enantioselectivity were observed with various malonate esters and substrates containing different methine substituents, although moving away from a *tert*-butyl group at the pro-stereogenic center had a detrimental impact on *ee*. Similar interactions are proposed to occur in other desymmetrization reactions from the same authors (*vide infra*).

**Figure 18:**
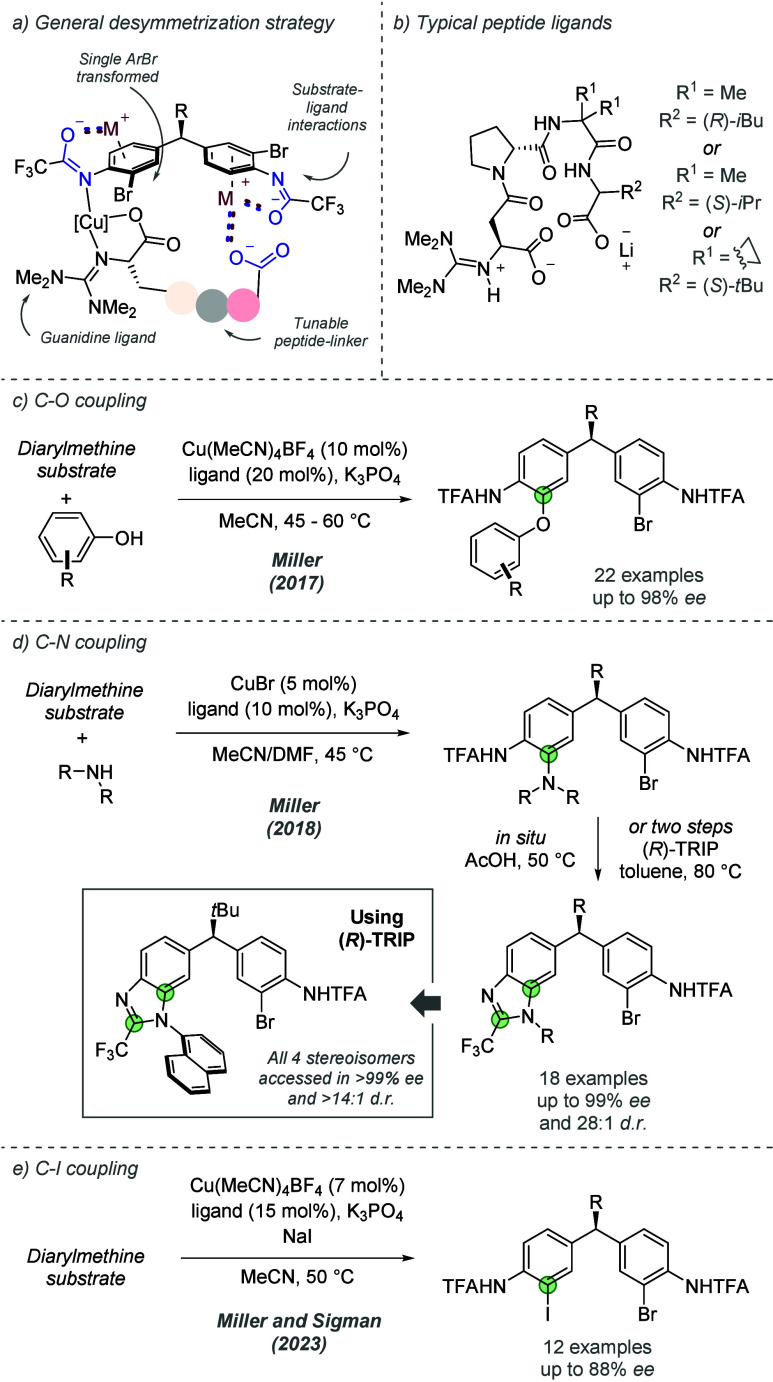
Chiral peptide ligands have been applied in several copper-catalyzed desymmetrizing reactions of diarylmethane substrates, in which ionic interactions are invoked.

Following this initial report, Miller and co-workers have demonstrated the generality of their peptide ligand design in a variety of desymmetrization reactions on related diarylmethine substrates (Figure 18a). The success of this ligand type is, in part, due to the highly tunable nature of the peptide backbone. While the arene substrate remains constant across different transformations, the varying reaction media and coupling partners are thought to impact the secondary structure of peptide ligands. Consequently, for a new transformation, optimization of amino acid monomers and overall peptide lengths is necessary to enable the optimal distal ionic interaction (Figure 18b). In 2017, based on the same hypothesis of ionic interactions between the terminal carboxylate residue and its associated cesium cation, they reported an enantioselective C–O coupling of diarylmethanes with phenols, also catalyzed by copper (Figure 18c).^47^ In addition to a broad scope of 4-substituted phenols, the reaction also displayed high levels of chemoselectivity when the phenol coupling partner contained other nucleophiles, such as indole or benzyl alcohol. Furthermore, excellent enantioselectivities and respectable yields were observed for sterically hindered 2-substituted phenols. Once again, moving away from a *tert*-butyl group at the pro-stereogenic center lowered *ee*, although cyclohexyl was also tolerated in this case. In related work, the same authors have also reported an atroposelective desymmetrizing C–O coupling of resorcinol-substituted quinazolinones using a similar dimeric peptide ligand (not shown).^48^ In this work, the substrate was structurally distinct from the diarylmethines of the previous work, with no comment on the nature of the interactions. In 2018, the same authors developed a copper-catalyzed enantioselective C–N coupling with anilines and aliphatic amines (Figure 18d).^49^ High levels of enantioselectivity were maintained and aliphatic amines and 4-substituted anilines were telescoped to benzimidazoles via cyclization with the neighboring trifluoroacetamide group. The authors found that in the case of 2-substituted anilines, cyclization onto the trifluoroacetyl group formed axially chiral benzimidazoles due to the restricted rotation about the C–N axis. Use of a single enantiomer of the chiral phosphoric acid TRIP allowed this cyclization to occur with very high diastereoselectivity. This work demonstrated catalyst control over two different stereogenic elements: a chiral copper complex controlled the desymmetrizing copper-catalyzed C–N coupling, presumed to be assisted by the ionic interactions proposed in the previous work, and a chiral phosphoric acid catalyst enabled the atroposelective cyclization. All four stereoisomers of a given benzimidazole were accessible through appropriate choice of either enantiomer of both catalysts. More recently, the same group demonstrated that the C–N coupling reaction could be applied in an intramolecular context after an initial C–C bond forming Ullman coupling placed the aniline in proximity to the second aryl bromide site (not shown).^50^ This work showcased elegant control in macrocyclizations of enantioenriched precursors through application of matched catalyst effects, with ionic interactions being thought to play an important role via the associated cation of the deprotonated peptide.^51^ The latest advance from Miller, Sigman and co-workers in this area was to develop an enantioselective Finkelstein reaction of diarylmethane substrates using a chiral peptide ligand (Figure 18e).^52^ After formation of an enantioenriched aryl iodide, strategic use of cross-coupling chemistry enabled the formation of several functionalized products, while leaving the bromide untouched. As before, considerable steric bulk at the methine bridge is essential for achieving high enantioselectivity and the nature of this trend was investigated using steric parametrization techniques. It was attributed to the impact of the methine substituent on aromatic ring conformations which ultimately affect enantioselectivity. Interestingly, a secondary kinetic resolution process was found to contribute to the high enantioselectivity obtained.

There has been a considerable interest in the development of chiral, functionalized NHC ligands for transition-metal-catalyzed processes.^53^ Among these, a class which contains peripheral hydroxyl groups on the *N*-substituted groups have been shown to constitute effective ligands within copper catalysis. Relevantly to this Review, there have been several cases where ionic interactions between the ligand-based alkoxide salt and the substrate are proposed to play a role in stereocontrol. While this Review will not cover the wider development and application of these ligands,^54^ the examples presented below aim to highlight the proposed importance of ionic interactions across several different reactions and ligand iterations.

**Figure 19:**
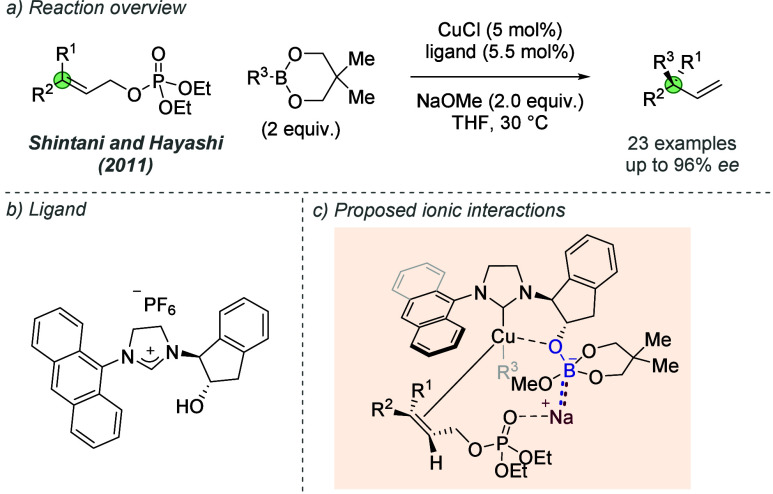
Use of a chiral hydroxylated NHC ligand in asymmetric allylic substitution in which an ion-pairing interaction with the sodium cation of a boronate intermediate is proposed.

In 2011, Shintani, Hayashi and co-workers reported the regio- and enantioselective allylic substitution of allyl phosphates with aryl- and alkenylboronates (Figure 19a).^55^ This work featured a chiral NHC ligand containing an indanol-derived hydroxyl, which afforded products in higher enantioselectivities when compared to previous ligand iterations (Figure 19b). In addition to the bidentate chelation provided by this ligand design, the authors proposed that the hydroxyl group has an additional role in orientating the allyl phosphate for the enantiodetermining oxidative addition step (Figure 19c). The phosphate group was proposed to coordinate to the sodium countercation of the anionic boronate species, which is itself held in proximity to the copper center through binding of the ligand hydroxyl. Although a tentative rationalization, this importance of the cation was further invoked in subsequent studies.

**Figure 20:**
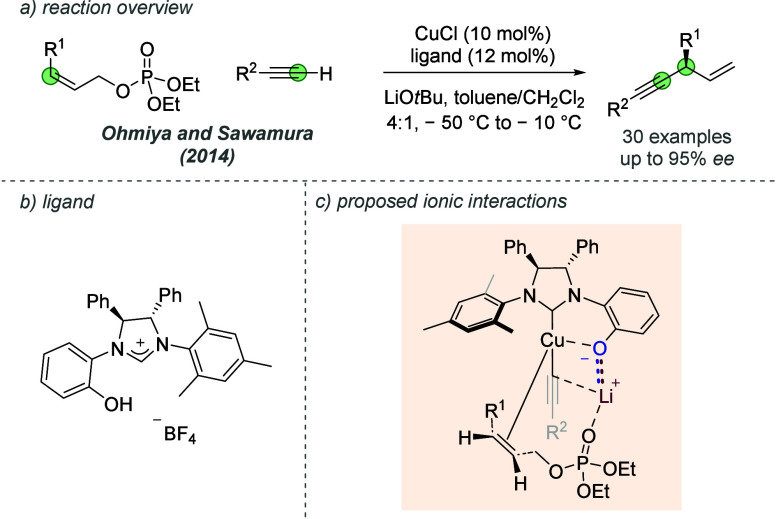
Application of a chiral hydroxylated NHC ligand in an asymmetric allylic substitution with terminal alkynes.

A related example where a similar interaction was invoked for a different allylic substitution reaction was by Ohmiya, Sawamura and co-workers in 2014 (Figure 20a).^56^ In this case, a related ligand featuring an *ortho*-phenol was required to effectively direct the coupling of a range of allyl phosphates and terminal alkynes in high regio- and enantioselectivities (Figure 20b). It was proposed that the bridging lithium cation between the phenolate and acetylide carbon provided an attractive interaction point for the phosphate leaving group, suitably positioning the alkene for the oxidative addition into the Cu(I) species and following steps (Figure 20c). The authors also proposed detailed models for enantioinduction, combining their findings on the importance of the ligand design and the associated countercation, to rationalize the success of *Z*-allyl phosphates compared to the corresponding *E* isomers.

**Figure 21:**
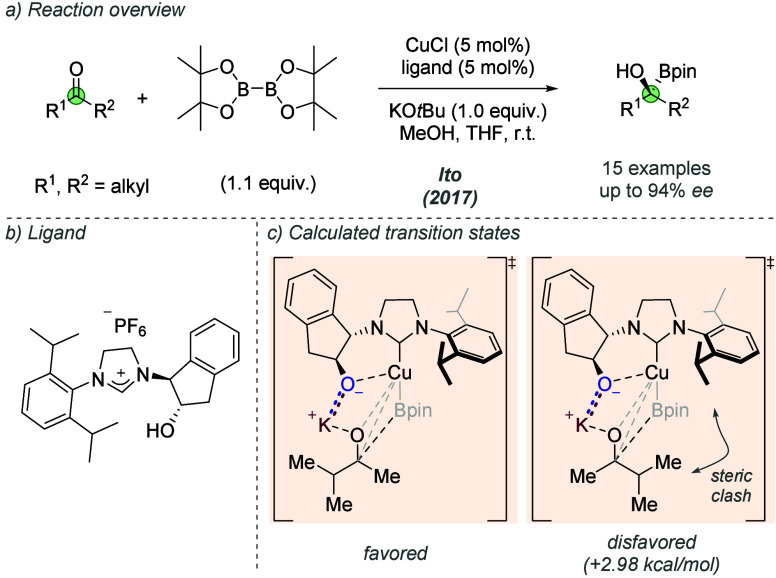
Enantioselective borylation of ketones using a phenolic NHC ligand.

In 2017, Ito and co-workers returned to a similar indanol-derived NHC ligand scaffold to enable catalytic enantioselective borylation of aliphatic ketones (Figure 21a, b).^57^ A range of alkyl-methyl ketones were borylated in high enantioselectivity. DFT calculations investigated the mode of stereocontrol and suggested that the ligand-based potassium alkoxide (Figure 21c) enables bidentate binding to the copper complex. Also important was the coordination of the carbonyl oxygen to the associated potassium cation of the alkoxide. These two factors ensured the formation of a conformationally rigid transition state in which *re*-face addition to the ketone was preferred, minimizing the steric clash with the bulky isopropyl groups on the NHC. Overall, the interactions involve an ionic interaction between the alkoxide and cation and also a cation-dipole interaction of the latter with the carbonyl undergoing addition. Consistently, a control experiment in which the free alcohol of the ligand was methylated afforded the product in low enantioselectivity (not shown).

**Figure 22:**
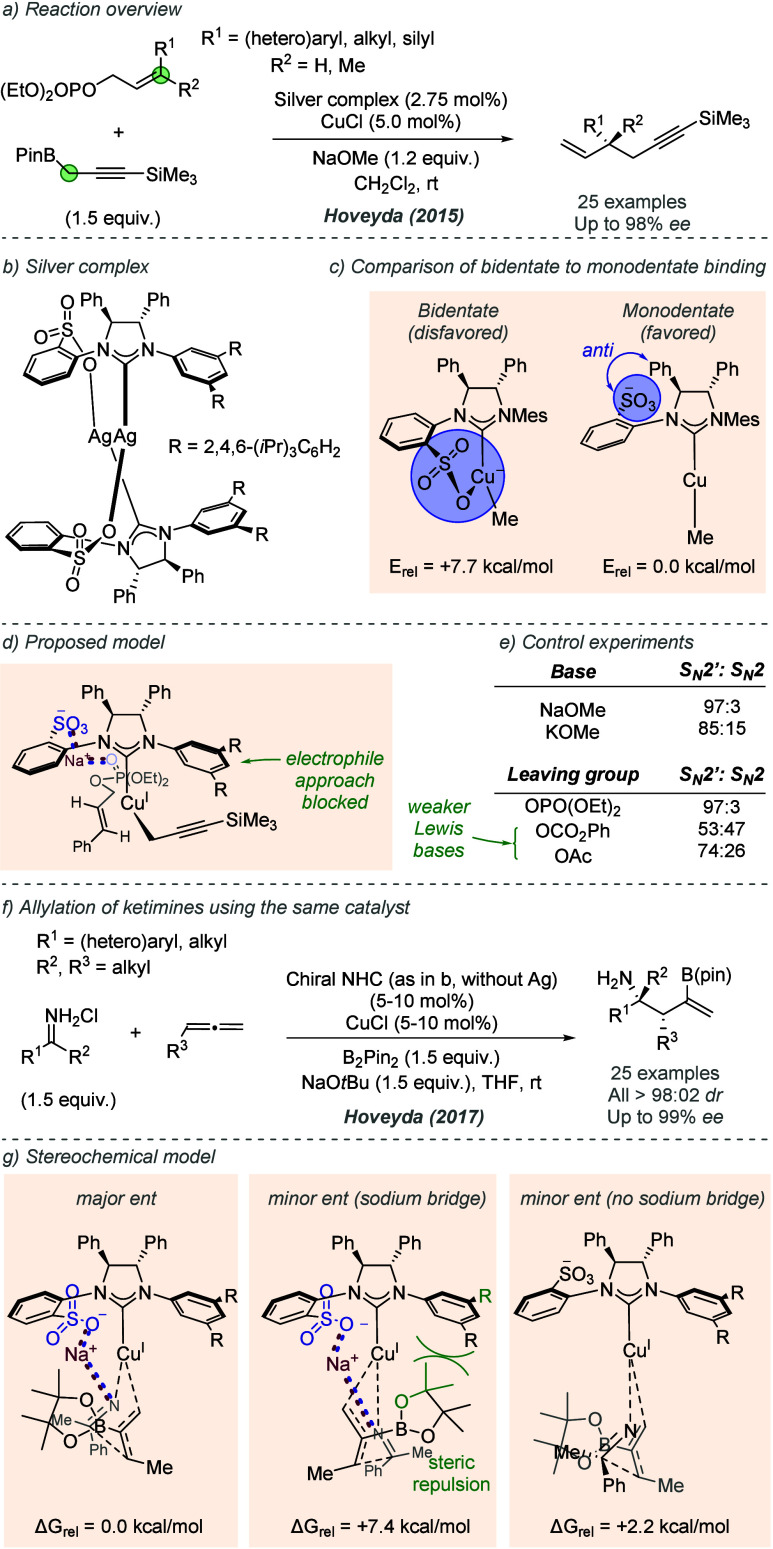
Use of sulfonated NHC ligand in copper catalyzed allylic substitution, and studies which led to the hypothesis of a bridging cation between the ligand and substrate.

A related series of functionalized NHC ligands containing peripheral sulfonate groups instead of phenols have been pioneered by Hoveyda and co-workers. The authors first disclosed copper-catalyzed conjugate additions using a sulfonated NHC in 2007,^58^ and have since reported a range of highly enantioselective reactions using this ligand, as well as closely related analogues.^59^ The authors initially proposed that the sulfonate could act as a Lewis base, coordinating to the copper center to form a bidentate NHC ligand.^59e^ Detailed mechanistic studies completed as part of a 2015 study into a highly branched-selective allylic substitution led the authors to advance an alternative hypothesis, in which they suggested that a bidentate copper complex is significantly higher in energy than a monodentate one where the sulfonate is uncomplexed (Figure 22a–c).^60^ After careful consideration, the authors advanced a proposal whereby a Lewis basic functional group on the substrate interacts with the alkali metal cation (in this case Na^+^) that is itself ion-paired with the catalyst sulfonate group (Figure 22d). This effect was proposed to be key to the excellent branched selectivity, which was observed to vary with the size of the alkali metal cation used, as well as the Lewis basicity of the group in the substrate (Figure 22e). The authors invoked an analogous set of interactions in several subsequent studies, such as a 2017 report on copper catalyzed allylation of imines using the same ligand, where it was proposed that the associated sodium cation of the sulfonate interacts with the nitrogen of the imine to provide organization and high *ee* (Figure 22f).^61^ A stereochemical model based on DFT calculations invoked an attractive interaction between the pendant sulfonate group, sodium cation, and imine, in analogy with the earlier work (Figure 22g). In the favored TS, the very large R substituents on the nonsulfonated ring force the B(pin) to occupy the front left quadrant (Figure 22g, left). In the disfavored TS, steric repulsion between the large group of the catalyst and the B(pin) were found to increase the energy of the proposed structure by +7.4 kcal/mol (Figure 22g, middle). The authors also modeled an alternative transition state leading to the minor enantiomer, in which the sodium bridge with the imine of the substrate was not formed (Figure 22g, right). The authors suggested that this was a more likely pathway to the disfavored enantiomer because of its lower relative energy.

Hoveyda and co-workers have invoked similar interactions in a number of other reports.^62^ In a detailed and comprehensive 2020 review which contained various unpublished stereochemical models to rationalize past transformations, the authors suggested that this “cation bridging” mode is likely to be occurring in several other copper-catalyzed transformations using this ligand.^63^

**Figure 23:**
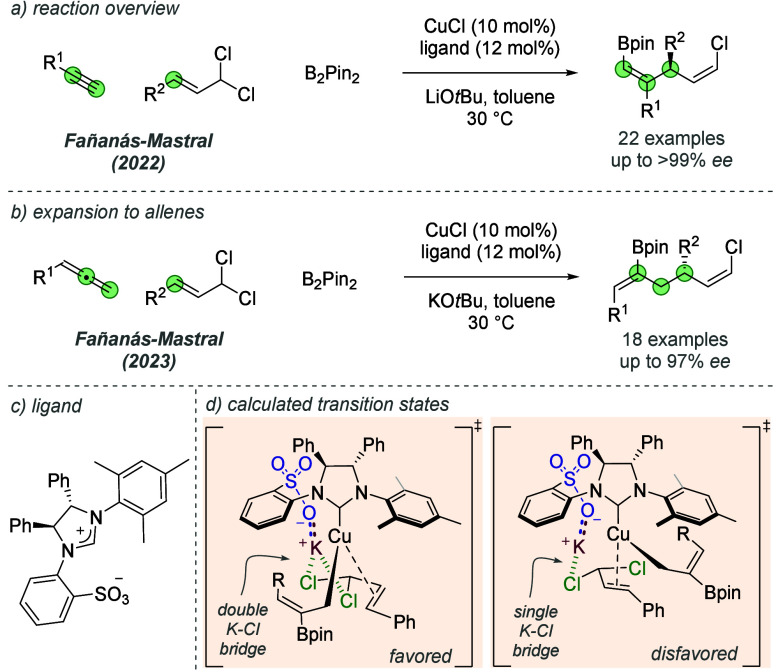
Further applications of sulfonated NHC ligands in enantioselective copper-catalyzed borylative coupling reactions.

In 2022, Fañanás-Mastral and co-workers demonstrated the application of similar chiral sulfonated NHCs in the copper-catalyzed enantioselective borylative coupling of terminal alkynes and allylic dichlorides (Figure 23a,c).^64^ The authors were motivated to use this new class of allylic substrate since it contains a leaving group, while also retaining an alkenyl chloride following the reaction. DFT calculations suggested an important role of the gem-dichloride group in engaging in a lithium cation bridge interaction with the ligand-based sulfonate, similarly to the interaction proposed by Hoveyda and co-workers. The same authors extended this reaction to encompass allene coupling partners (Figure 23b).^65^ The sulfonated NHC ligand used in these studies (Figure 23c) gave superior control over numerous aspects of selectivity when compared to other hydroxylated NHC scaffolds. After formation of a σ-allyl-Cu(I) intermediate and coordination of the allylic dichloride, two competing transition states were proposed through DFT for the subsequent stereodetermining oxidative addition (Figure 23d). In the disfavored transition state, a single attractive interaction between the sulfonate potassium countercation and a substrate chloride unit was invoked. In the favored transition state, a double cation bridge with both chloride units was proposed; the authors suggested that the additional stabilization from the second interaction accounted for the observed enantioselectivity.

#### 2.2.3 Iridium

**Figure 24:**
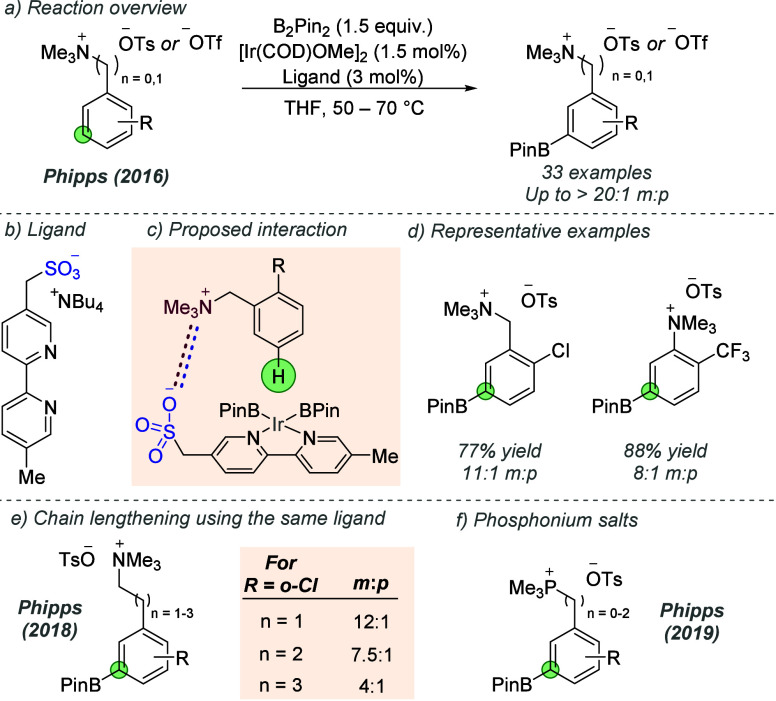
*meta*-Selective C–H borylation directed by an ion-pairing interaction using a sulfonated bipyridine ligand.

Aryl-boron bonds are readily convertible into a range of other functional groups. Achieving selective borylation of similar aryl C–H bonds can therefore be useful for the construction of complex molecules; unsurprisingly, it has become a major research area.^66^ However, the primarily steric-based regioselectivity outcomes means that some substrates deliver mixtures of regioisomers.^67^ A growing number of researchers, commencing with the pioneering report of Kanai and Kuninobu in 2015 which used hydrogen bonding, have designed ligands for iridium that are intended to engage in attractive noncovalent interactions with the substrate to direct regioselectivity.^68^ A strategy that utilized ionic interactions was first explored by Phipps and co-workers, who in 2016 reported a *meta*-selective iridium-catalyzed borylation of aromatic quaternary ammonium salts (Figure 24a).^69^ A series of bipyridine-based anionic ligands, each bearing a peripheral anionic sulfonate group, were prepared, one of which (Figure 24b) was found to catalyze borylation with high *meta* selectivity relative to the ammonium group. The proposed interaction involved ion pair formation between the cationic ammonium on the substrate and the anionic sulfonate group on the ligand (Figure 24c). This interaction directs the iridium catalyst to undergo the selectivity-determining oxidative addition at the *meta* C–H bond, with support for this interaction provided by several control experiments. The reaction was applied to benzylamine and aniline-derived quaternary ammonium salts (Figure 24d). A subsequent report by the same group extended the scope of the transformation to ammonium salt substrates bearing longer, more flexible alkyl chains (Figure 24e). Good *meta*-selectivity could be obtained on quaternized phenethylamines and phenylpropylamines, although selectivity was eroded as the chain was extended further than this (e.g., 4:1 *m*:*p* for n = 3 in Figure 24e).^70^ Interestingly, despite the chain length increasing in the substrate, the same ligand as in the initial study was found to be optimal for all when ligand variants were evaluated. The reaction was also applied to phosphonium salts (Figure 24f).^71^ A phenylpropylammonium substrate was subjected to a DFT investigation by Datta and co-workers in 2022.^72^ This enabled the selectivity-determining oxidative addition to be compared for *meta* vs *para* C–H activation. The activation energy for oxidative addition into the *meta* C–H bond was calculated to be lower than the *para* by 5.3 kcal/mol, due to a stronger ion pairing interaction in the former due to their closer proximity, in line with the original hypothesis.

**Figure 25:**
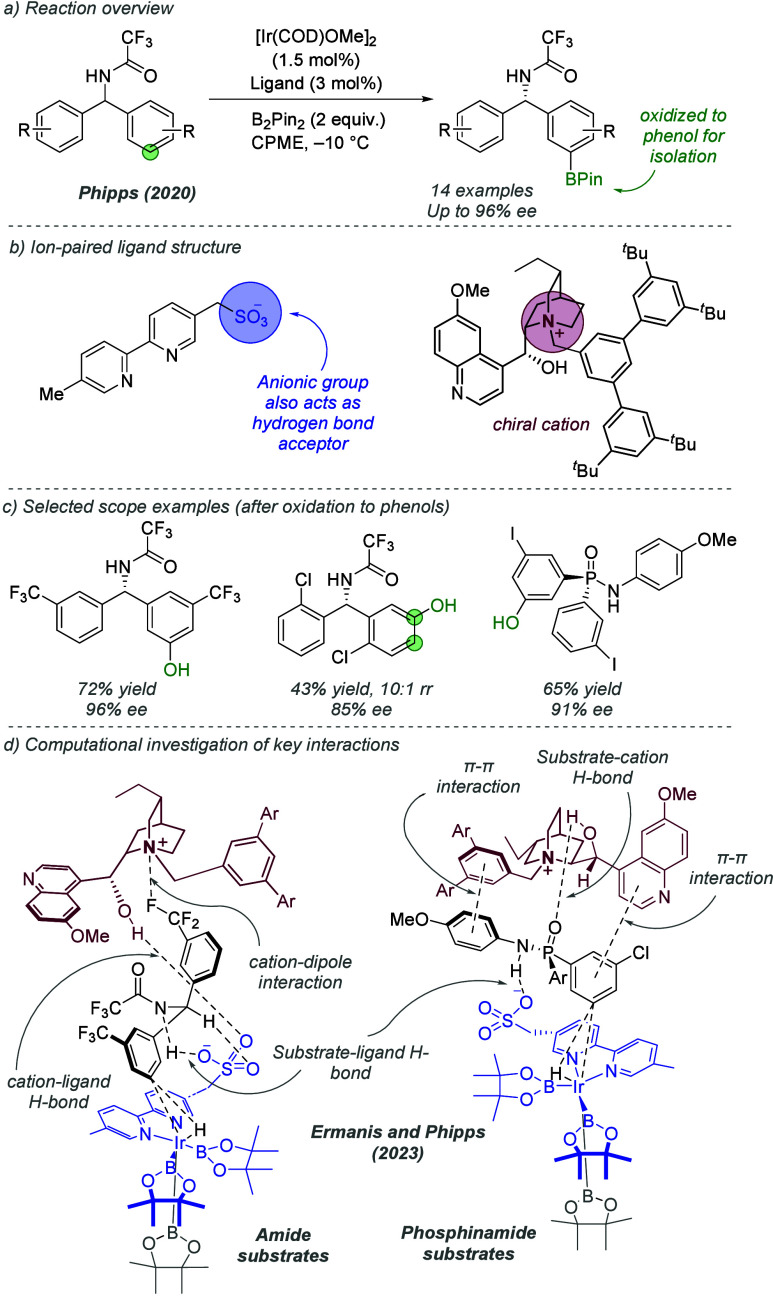
Enantioselective C–H borylation directed by a sulfonated bipyridine ligand ion-paired with a chiral cation.

The same ligand could be used with neutral amide-containing substrates–the proposed substrate–ligand interaction is a hydrogen bond and therefore outside the scope of this Review.^73^ However, this substrate–ligand hydrogen bonding mode of interaction was later combined with an ionic interaction with an associated chiral cation, which allowed the development of an enantioselective borylation of benzhydrylamides (Figure 25a).^74^ The ligand used in the previous regioselective work was always associated with a tetrabutylammonium cation to permit solubility in nonpolar solvents. The authors questioned whether replacing this achiral cation with a chiral cation, specifically one derived from a cinchona alkaloid of the type which has become established in chiral phase transfer catalysis, could allow control of enantioselectivity in the Ir-catalyzed borylation reaction (Figure 25b). After optimization of the reaction conditions and modification of the cation structure to contain a bulky, substituted benzyl group, a series of amides bearing enantiotopic aryl groups were desymmetrized to afford chiral borylated products with very high levels of enantioselectivity, controlled solely by the associated chiral cation. Some substrates posed no site-selectivity choice (e.g., Figure 25c, left), but for several substrates where there was a choice, good catalyst-controlled selectivity for the *meta*-position was also observed, demonstrating the ability of the catalyst to control both selectivity aspects (e.g., Figure 25c, middle). The method was also applied to diaryl phosphinamide substrates which afforded *P*-chiral products upon desymmetrisation (e.g., Figure 25c, right). An in-depth DFT study by Ermanis, Phipps, and co-workers investigated the interactions between the substrate, ligand, and chiral cation that were responsible for the observed enantioselectivity, alongside supporting experimental data (Figure 25d).^75^ The study revealed distinct binding modes between the substrate and catalyst for amide and phosphinamide substrates. The amide substrates were proposed to engage in two substrate–ligand hydrogen bonds involving the ligand sulfonate group; a stronger one with the amide NH and a weaker one with the diarylmethane. The chiral cation is associated with the ligand through ionic interactions but also a strong hydrogen bond with the free hydroxyl group of the alkaloid derivative. Finally, in the substrate under investigation, a cation-dipole interaction was identified between the polar trifluoromethyl group on the arene and the chiral cation (Figure 25d, left). Phosphinamide substrates differed in two key ways (Figure 25d, right). First, the hydroxyl group on the cation was found to engage in hydrogen bonding directly with the substrate through its phosphoryl oxygen, rather than with the ligand. Second, two π–π interactions between arenes on the substrate and cation were identified.

**Figure 26:**
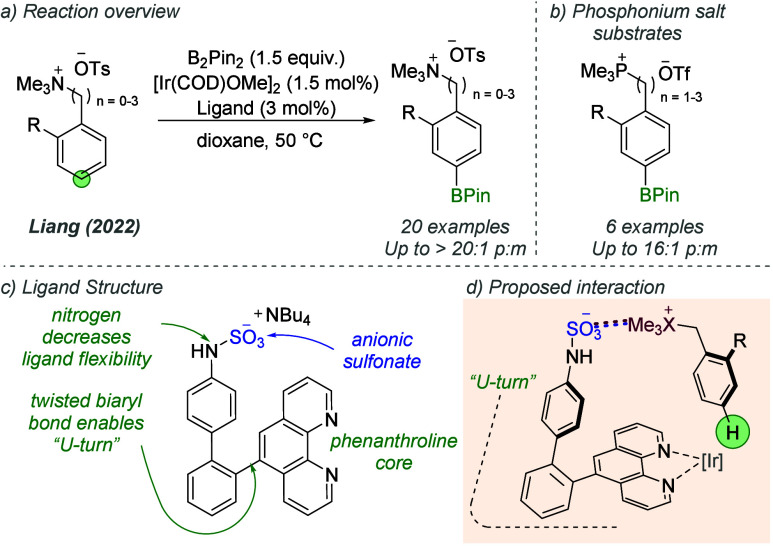
*Para*-selective C–H borylation directed by ion pairing using an extended sulfonated phenanthroline ligand.

The system developed by Phipps for pairing quaternary ammonium-containing substrates with anionic sulfonate-containing ligands has been extended to *para*-selective borylation by Liang and co-workers, by modifying the *meta*-selective ligand design reported previously (Figure 26).^76^ Both quaternary ammonium (Figure 26a) and phosphonium salts (Figure 26b) were effective substrates, and a range of tether lengths between the arene unit and onium salt were tolerated. In a divergence from the bipyridine-based ligands previously developed, this report used a phenanthroline as the ligand core, with two arene units incorporated into the tether between the sulfamate and the phenanthroline. This design enabled effective positioning of the sulfonate substituent within a “U-turn” structure formed by the *ortho*-substituted phenyl ring (Figure 26c). An NH unit located between the sulfonate and arene unit afforded higher *para*-selectivity than CH_2_, attributed to its lower flexibility. The ionic interaction between the onium salt of the substrate and the sulfonate group of the ligand directed a *para*-selective C–H oxidative addition, with high selectivity (Figure 26d).

**Figure 27:**
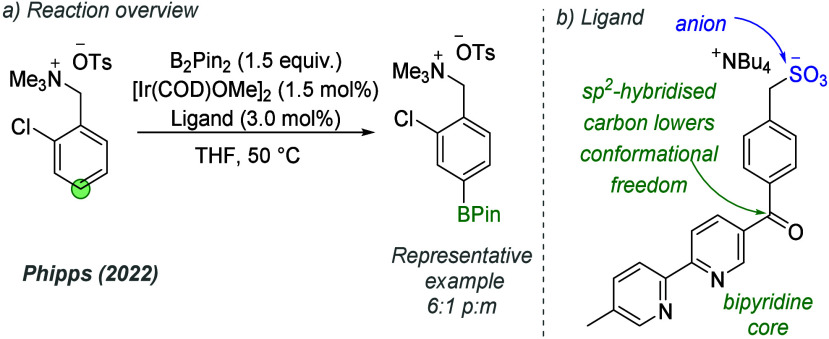
*para*-Selective borylation with extended sulfonated bipyridine ligand.

The same year, a *para-*selective borylation of ammonium salts was reported by Douthwaite and Phipps (Figure 27a).^77^ Selectivities were lower than those obtained by Liang and co-workers, up to a maximum of 6:1 *p*:*m*. Several ligands based on a bipyridine core and an arene unit between the bipyridine and the sulfonate were investigated; it appears that this single arene spacer was not sufficient to realize high selectivity. The most effective ligand (depicted in Figure 27b) contained an sp^2^-hybridized carbonyl between the bipyridine and arene, which was speculated to aid regioselectivity by lowering the conformational freedom of the ligand.

**Figure 28:**
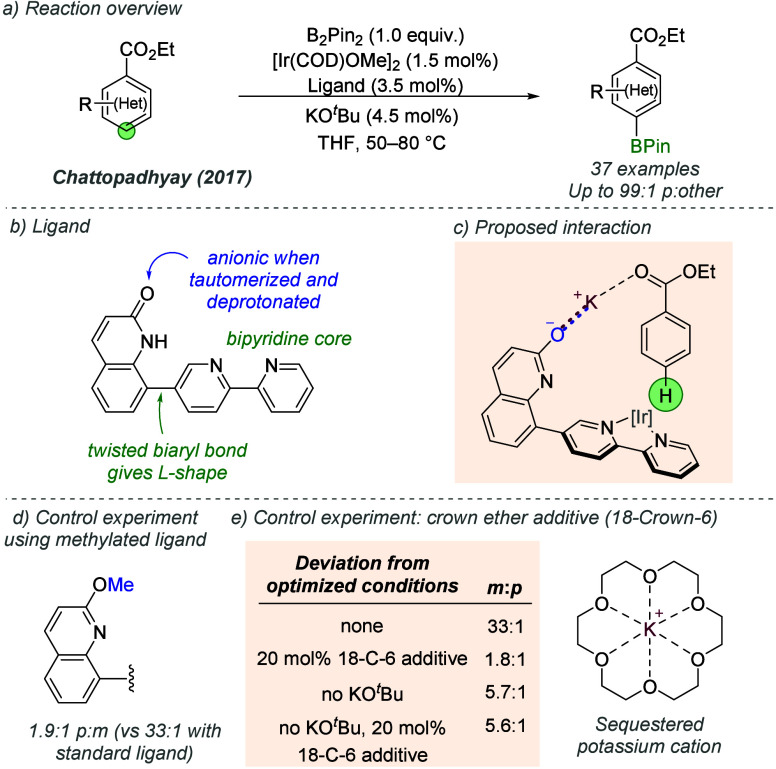
*para*-Selective borylation of aromatic esters with L-shaped bipyridine ligand.

Ionic interactions involving a charged ligand, via its associated countercation, have also been harnessed in the site-selective borylation of neutral substrates. In 2017, Chattopadhyay and co-workers reported the *para-*selective borylation of aromatic esters (Figure 28a).^78^ The ligand was based on a bipyridine core attached to a quinolone through a twisted biaryl bond, forming an “L-shaped” structure (Figure 28b). The quinolone lactam functioned as a precursor to an anionic group; when deprotonated under the reaction conditions by KO*^t^*Bu it forms the corresponding potassium salt. The proposed interaction involves an ion pair between the anionic ligand and potassium cation. The latter can interact with the carbonyl group of the substrate, directing the iridium catalyst toward the arene *para*-position (Figure 28c). Evidence for the proposed interaction was provided by an experiment with a methylated version of the optimal ligand–the high *para* selectivity was greatly eroded (Figure 28d). They also ran the reaction in the presence of 18-crown-6 and saw a similar reduction, providing further support for the importance of the potassium cation in controlling regioselectivity (Figure 28e). This effect was only observed in the presence of KO*t*Bu, providing evidence for the sequestration of the potassium cation by 18-Crown-6 (entries 3 and 4).

**Figure 29:**
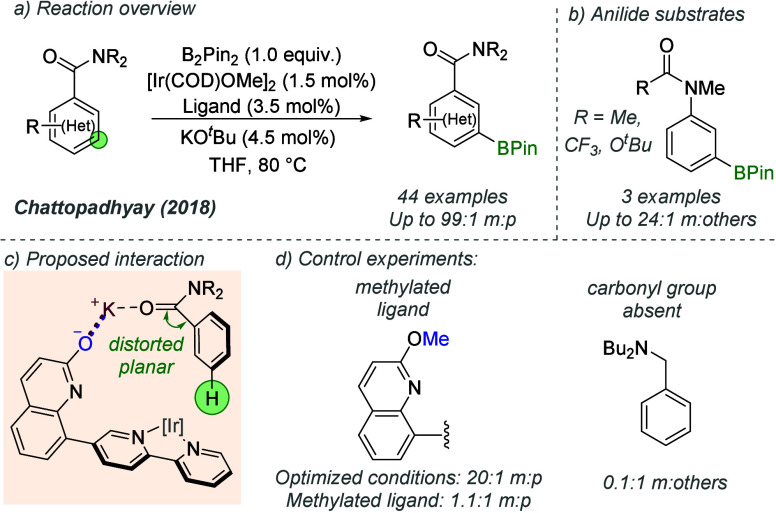
*meta*-Selective borylation of amides using L-shaped bipyridine ligand.

Chattopadhyay and co-workers used the same ligand, and almost identical reaction conditions, in the *meta*-selective borylation of aromatic amides (Figure 29a).^79^ Anilides, in which the amide nitrogen is attached directly to the aromatic ring, were also effective substrates (Figure 29b). The selectivity contrasts with that observed in the previous report on esters. The authors explained this by proposing that the amide adopts a distorted planar geometry, altering the structure of the transition state to favor C–H activation at the *meta*-position, while invoking a similar interaction to that described in the previous work (Figure 29c). Support for this interaction was provided by the poor regioselectivity obtained when an *O*-methylated ligand was used or when an amine bearing no carbonyl group was tested (Figure 29d).

#### 2.2.4 Rhodium

**Figure 30:**
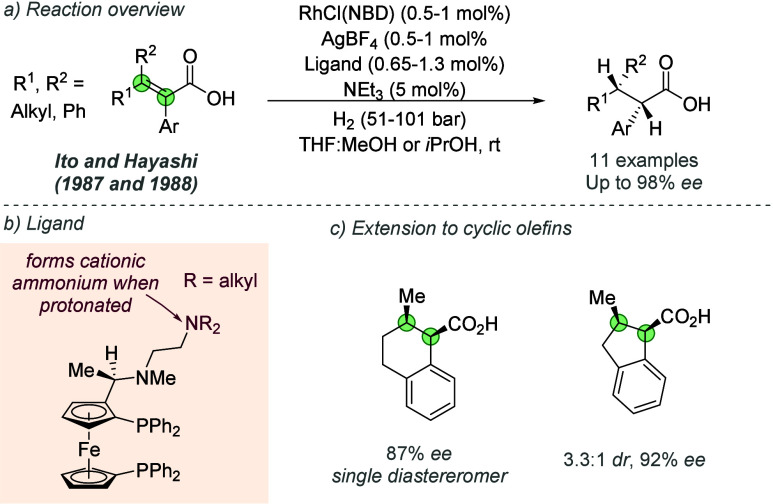
Rh-catalyzed hydrogenation of trisubstituted acrylic acids, with a ligand capable of ionic interactions with the substrate.

Following on from their pioneering work on the gold-catalyzed aldol reaction (see Figure 2), Hayashi, Kawamura and Ito applied the same ligands to Rh-catalyzed hydrogenation of trisubstituted acrylic acids with considerable success (Figure 30a and b).^80^ The ligand design was the same as in the earlier work, though the alkyl substituents on the terminal amine could be varied slightly to afford optimal enantioselectivity for each substrate (Figure 30b). In a similar fashion they proposed that the amine side chain of the ligand deprotonates the substrate and permits attractive ionic interactions between the carboxylate and the protonated tertiary amine. It seems feasible that in polar protic solvents, extensive proton transfer will occur, resulting in Coulombic interactions in addition to hydrogen bonding. This both accelerates the reaction and assists in controlling the facial selectivity in the delivery of hydrogen. The authors also showed two cyclic substrates, with excellent enantioselectivities (Figure 30c).

**Figure 31:**
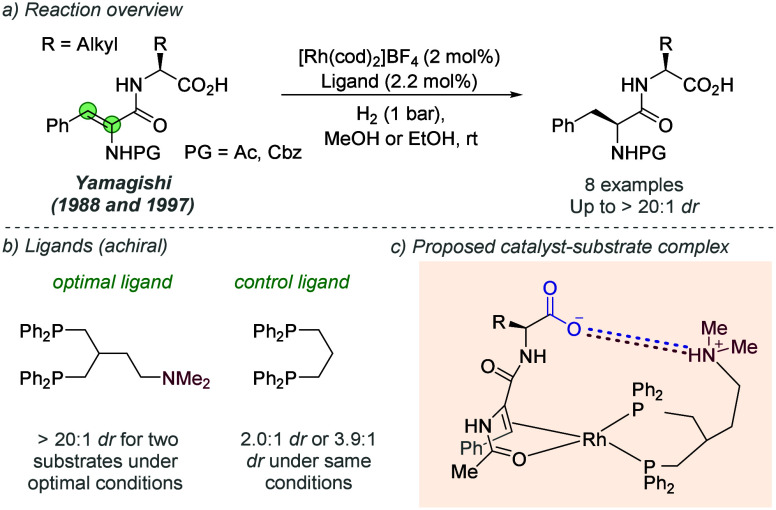
Diastereoselective hydrogenation of dehydropeptides with an achiral phosphine ligand.

Studies from Yamagishi and co-workers on diastereoselective dehydropeptide hydrogenation also made use of chiral phosphine ligands bearing pendant amine groups, which were proposed to similarly deprotonate the terminal acid and engage in an ionic interaction.^81^ This reaction is diastereoselective, rather than enantioselective, meaning that an achiral phosphine ligand may also be capable of achieving diastereoselectivity. In 1988, Yamagishi and co-workers disclosed such a ligand, capable of catalyzing the hydrogenation of similar substrates with excellent diastereoselectivities (Figure 31a).^82^ The bidentate phosphine ligand contains a pendant amino group, which was proposed to form a cationic ammonium species capable of interacting with the carboxylate group of the substrate (Figure 31b). A control ligand without this amine substituent afforded much lower diastereoselectivity, as did substrates in which the free carboxylic acid of the substrate was replaced with an ester (not shown). In 1997, the authors conducted detailed spectroscopic investigations of several rhodium-diphosphine complexes, and proposed a structure for the catalyst-substrate complex invoking the key substrate–ligand interaction (Figure 31c).^83^

**Figure 32:**
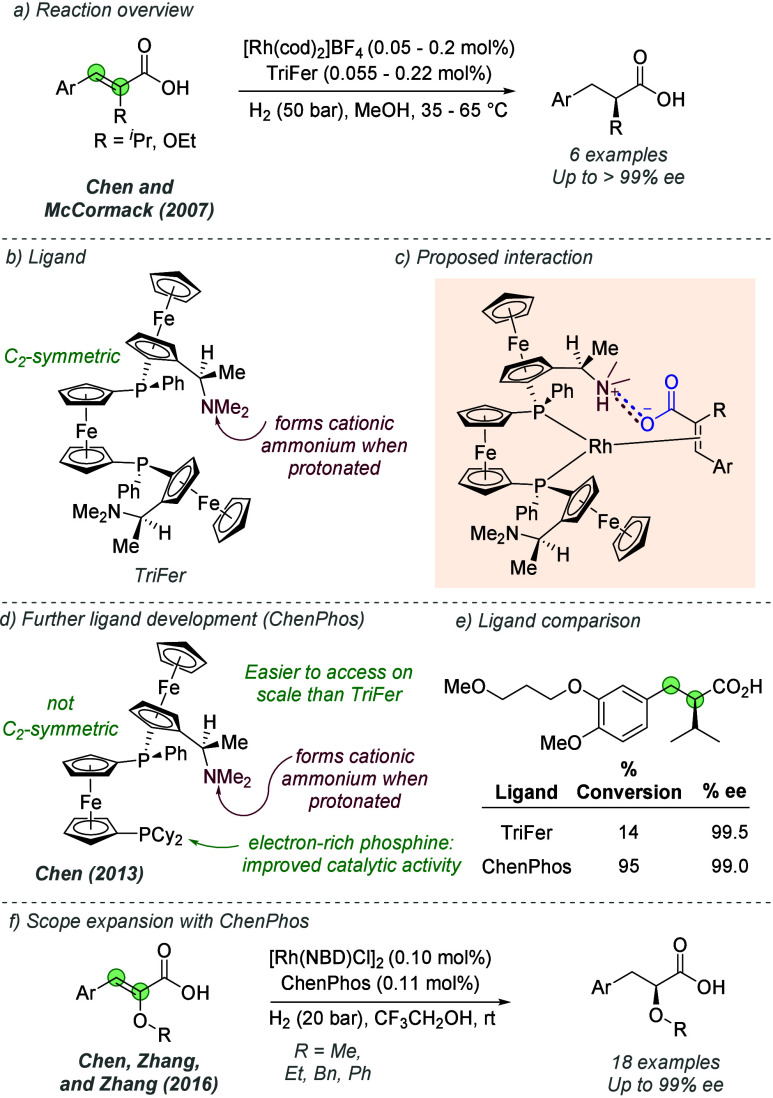
Enantioselective hydrogenation catalyzed by TriFer and ChenPhos ligands.

Building on those important earlier advances, a subsequent catalyst design that incorporated outer sphere ionic interactions in enantioselective rhodium-catalyzed hydrogenation came from Chen, McCormack and co-workers in 2007 (Figure 32a).^84^ Six α-substituted cinnamic acids were hydrogenated to afford products in high enantioselectivities. This was enabled by C_2_-symmetric ligand TriFer, which combines *P*-chirality alongside planar and *C*-chirality derived from Ugi’s amine (Figure 32b). In the proposed transition state, the amine substituent of the ligand is protonated by the carboxylic acid substrate and the ensuing ionic interaction, between the *in situ*-formed ammonium and substrate carboxylate, was proposed to explain the high enantioselectivity and activity of the catalytic system (Figure 32c). It is likely that hydrogen bonding between the amine N–H and carboxylate also plays a significant role. With the ionic interaction invoked, the standard quadrant rule could then be used to rationalize the absolute configuration of the products. Control experiments showed that if the corresponding ester was used there was no reduction, consistent with the hypothesis. Further ligand exploration from Chen and co-workers led to the development of ChenPhos in 2013 (Figure 32d).^85^ In this new ligand, one of the *P*-chiral phosphine substituents in TriFer is replaced with an achiral electron-rich phosphine group. This breaks the C_2_-symmetry present in TriFer, and results in improved catalytic activity. ChenPhos retained one tertiary amine, for an analogous outer sphere ionic interaction, and proved easier to synthesize on scale. A comparison of the activities and selectivities of the two ligands on a model substrate is shown in Figure 32e. The incrementally higher enantioselectivity obtained with TriFer (99.5 vs 99.0) was more than offset by the better conversion observed with ChenPhos, under otherwise identical conditions.

Chen, Zhang, Zhang, and co-workers expanded the scope of hydrogenation reactions with ChenPhos to α-oxy functionalized α,β-unsaturated acids in 2016 (Figure 32f).^86^ Reactivity and enantioselectivity were found to strongly depend on the solvent for this substrate class, with CF_3_CH_2_OH affording optimal results. A subsequent report from Jiang, Zhang, and co-workers in 2020 expanded the scope of α-aryloxy carboxylic acids (not shown).^87^ Jiang, Lan, Cheng, and co-workers have developed an enantioselective hydrogenation of α-substituted dienoic acids with TriFer as the ligand.^88^ In this case, DFT calculations were carried out, suggesting that the carboxylate of the substrate directly ligates the Rh(III) metal center. Meanwhile, the protonated ammonium of the ligand acts as a hydrogen bond donor to the carboxylate carbonyl, rather than an outer sphere ionic interaction that had been proposed in previous similar works. Distinguishing between the mechanistic possibilities is challenging, but the outer sphere ionic design has led to several scope advances in this area, precise origin of selectivity aside.

**Figure 33:**
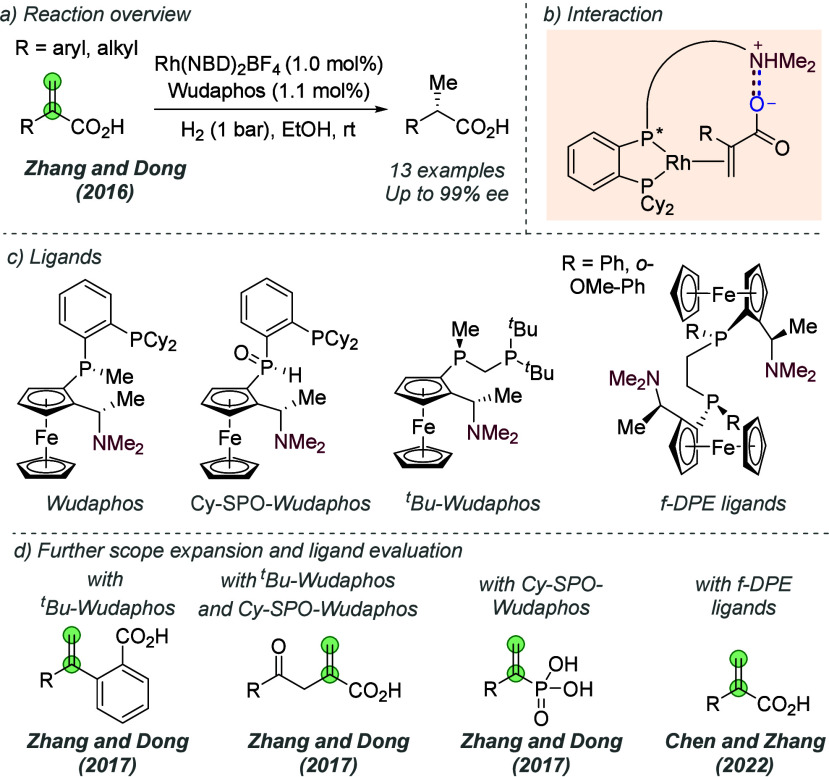
Various enantioselective hydrogenation examples catalyzed by Wudaphos and f-DPE ligands.

In 2016, Zhang, Dong, and co-workers reported an enantioselective hydrogenation of 2-substituted acrylic acids (Figure 33a).^89^ A new chiral bisphosphine ligand, Wudaphos, was developed and an outer sphere ionic interaction between ligand and substrate was proposed in analogy with the previously discussed work (Figure 33b). In the years that followed, a number of reports have explored the refinement of ligand structure and their application to various alkene substrate classes (for examples, see Figure 33c and d). *^t^*Bu-Wudaphos was applied to α,α-disubstituted terminal olefins bearing a carboxy-directed group^90^ and α-methylene-γ-keto-carboxylic acids,^91^ while SPO-Wudaphos was applied to α-methylene-γ-keto-carboxylic acids and substituted ethenylphosphonic acids.^92^ Further ligand design led to the development of C_2_-symmetric chiral ferrocene-based diphosphinoethane ligands, termed f-DPE ligands.^93^ These ligands were effective at catalyzing the asymmetric hydrogenation of the original 2-substituted acrylic acid substrate class.

**Figure 34:**
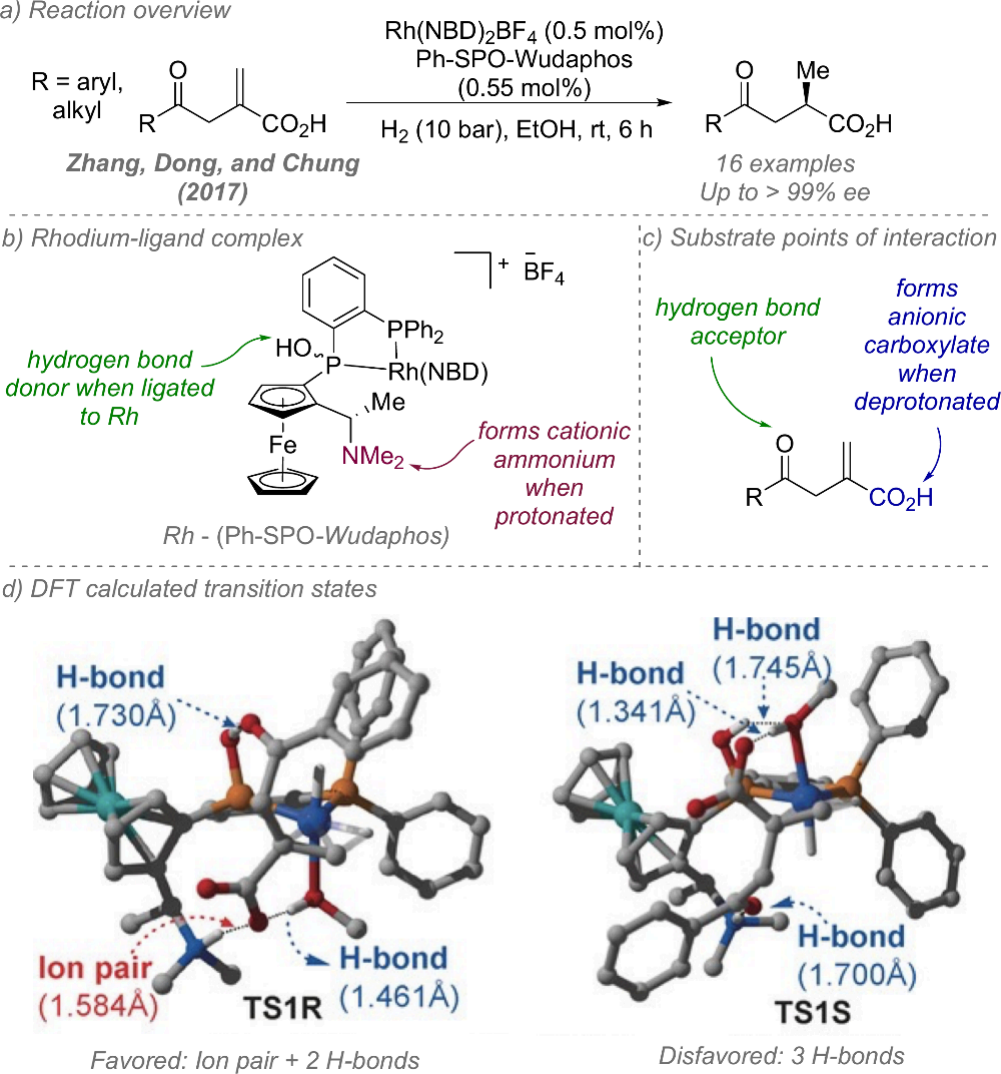
Enantioselective hydrogenation of α-methylene-γ-ketocarboxylic acids controlled by ion-pairing and hydrogen bonding interactions. Figure in panel d reproduced with permission from ref (94). Copyright 2017 Wiley-VCH Verlag GmbH & Co. KGaA, Weinheim.

Upon ligation to rhodium, the secondary phosphine oxide of SPO-Wudaphos ligands can form a hydroxyl group resembling that of phosphinous acid,^94^ and it is proposed that this can act as a hydrogen bond donor (Figure 34b). Zhang, Dong, Chung and co-workers utilized this feature in the enantioselective hydrogenation of α-methylene-γ-ketocarboxylic acids (Figure 34a). This multifunctional ligand was paired with substrates bearing an anion precursor in the carboxylic acid and a hydrogen-bond acceptor in the ketone (Figure 34c). DFT calculations revealed a favored transition state involving both ion pairing and hydrogen bonding interactions, and a disfavored transition state where the ion pair is unable to form (Figure 34d).

**Figure 35:**
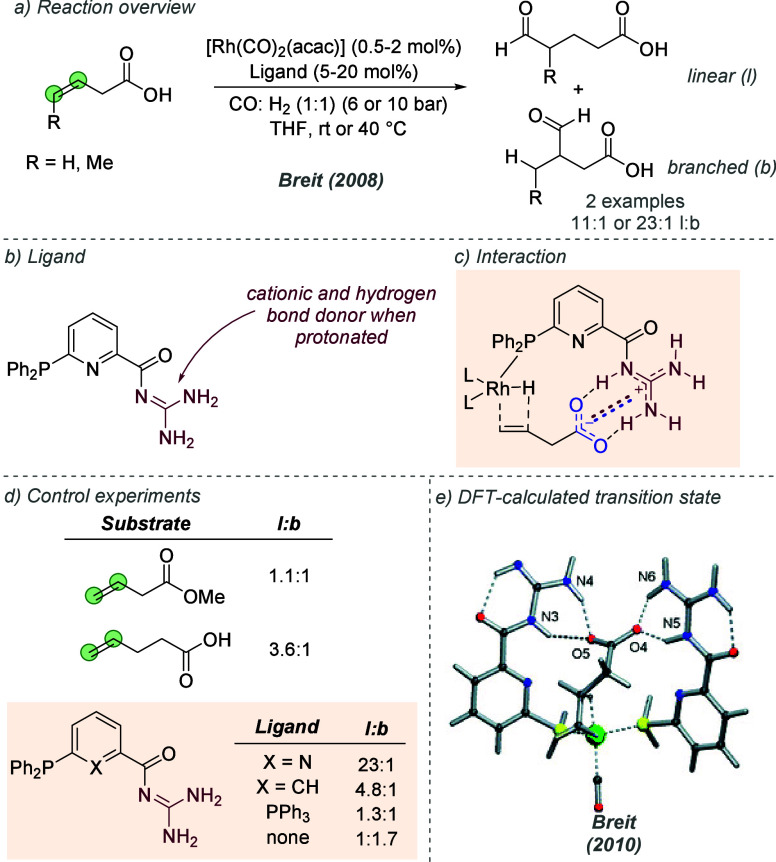
Regioselective hydroformylation directed by a carboxylic acid. Figure in panel e reproduced with permission from ref (96). Copyright 2010 Wiley-VCH Verlag GmbH & Co. KGaA, Weinheim.

Hydroformylation is an important process, often rhodium catalyzed, which presents a linear vs branched selectivity challenge in many substrates. Šmejkal and Breit presented an innovative solution to this problem that uses a combination of ionic and hydrogen bonding interactions in a manner related to the above strategies–an acidic substrate is deprotonated by a ligand for rhodium which bears a basic group. This was first demonstrated in the linear-selective hydroformylation of unsaturated carboxylic acids (Figure 35a).^95^ Two substrates, bearing a terminal or *cis*-configured olefin which was β,γ-unsaturated with respect to a carboxylic acid directing group, were selectively hydroformylated with good linear:branched selectivities. The triarylphosphine ligand contained a guanidine unit, which was proposed to become protonated by the carboxylic acid substrate under the reaction conditions (Figure 35b). Through a combination of hydrogen bonding and ion pairing, the carboxylic acid-guanidine interaction was proposed to direct the rhodium catalyst toward the distal position of the olefin (Figure 35c). A control experiment in which the carboxylic acid directing group was replaced with an ester afforded poor selectivity, providing evidence for the proposed interaction, while a γ,δ-unsaturated carboxylic acid showed much poorer 3.6:1 selectivity, demonstrating the relevance of the distance between the directing group and reactive site (Figure 35d). Other ligands gave significantly poorer outcomes. In 2010, a detailed mechanistic study was carried out using DFT calculations, and this surprisingly suggests that two ligand-based guanidinium units from two separate ligands on rhodium are involved in binding a single carboxylate (Figure 35e).^96^ One of these is neutral and the other cationic, with four distinct hydrogen bonds identified. Clearly there is a strong hydrogen bonding component to these binding interactions, but the ionic contribution is likely also important–in practice it is difficult to disentangle the two. An additional report from 2008 showed that the same ligand could also be applied to decarboxylative hydroformylation of α,β-unsaturated acids.^97^ In a later development, a regioselective hydroformylation of internal alkynes was developed, and in this case a catalytic amount of a Bro̷nsted acid (equivalent to the ligand loading) was added.^98^ This was proposed to protonate the guanidine group on the ligand and permit better hydrogen bonding interactions with the carboxylic acid substrate in its protonated form; in this case, ionic interactions are not invoked as a component of the attractive forces.

**Figure 36:**
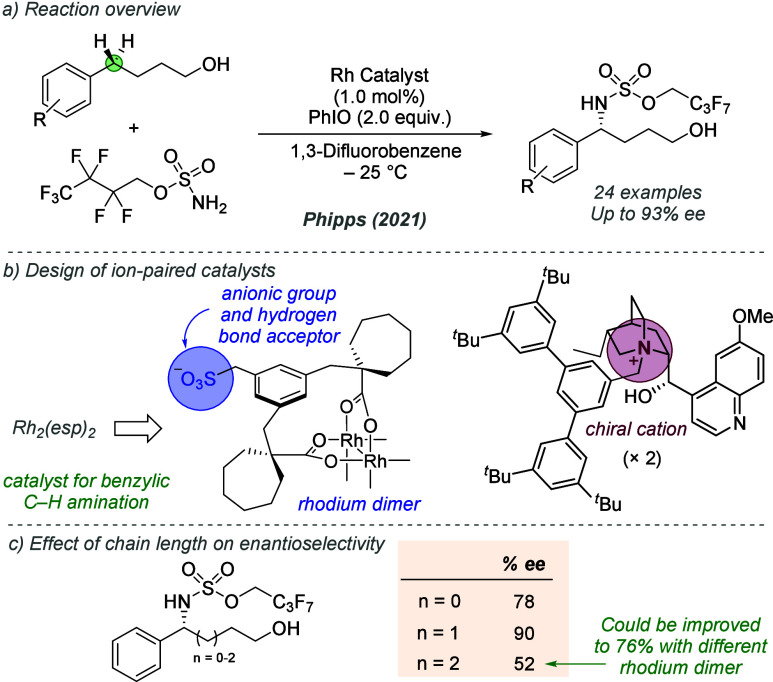
Enantioselective C–H amination of alcohols using an anionic rhodium complex paired with chiral cations.

Moving away from hydrogenation and hydroformylation, outer sphere ion pairing interactions have also been applied to catalytic nitrenoid transfer using rhodium complexes, for the enantioselective construction of C–N bonds.^99^ In 2021, Phipps and co-workers reported the enantioselective benzylic C–H amination of substrates bearing a pendant alcohol directing group (Figure 36a).^100^ Building on their previous work, in which they used anionic bipyridine ligands associated with a chiral cation to realize enantioselective arene borylation (*vide supra*), they designed an anionic, bis-sulfonated version of Rh_2_(esp)_2_, an established catalyst for the amination of benzylic C–H bonds. Chiral cinchona alkaloid-derived chiral cations, similar to those used in the earlier work with iridium catalysis, could be associated with these to give a chiral ion-paired Rh(II/II) dimer (Figure 36b). The authors anticipated that a hydrogen bonding interaction with the substrate may be necessary to provide organization, and it was proposed that the substrate primary alcohol could interact with the ligand sulfonate group in this manner. This sulfonate group would additionally engage in ion pairing with the chiral cation, providing a well-defined chiral environment for the C–H amination. A chain length of four methylenes between the alcohol and arene (phenylbutanols) was found to give optimal *ee*; under these initial conditions extending or shortening this chain gave reduced selectivity, although this could be tuned to some extent by cyclizing the geminal dimethyl groups on the ligand scaffold (Figure 36c, cycloheptyl shown). Within the phenylbutanol substrate class, a range of substituted arenes could be tolerated. A control experiment without the primary alcohol gave very poor reactivity and selectivity (not shown). The quinoline nitrogen was proposed to undergo reversible axial ligation to the rhodium complex, which appeared to improve reactivity compared to Rh_2_(esp)_2_.

**Figure 37:**
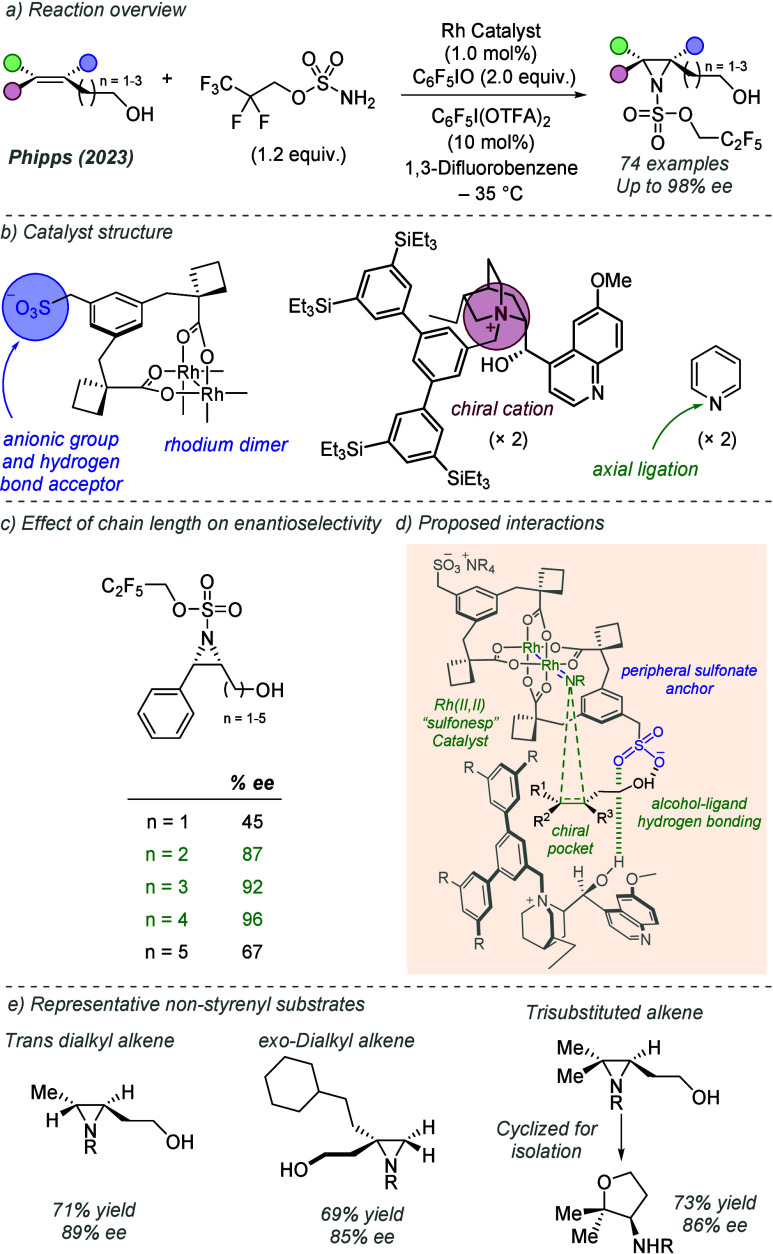
Enantioselective aziridination of alkenyl alcohols using anionic rhodium complexes paired with chiral cations.

Phipps and co-workers subsequently applied the same catalyst family to alcohol-directed enantioselective alkene aziridination (Figure 37a).^101^ Building on the C–H amination, the reaction conditions were further refined to allow high enantioselectivity across a wide range of substrates. Specifically, C_6_F_5_IO was used in place of PhIO as a more soluble oxidant at low temperature, and a substoichiometric additive of C_6_F_5_I(OTFA)_2_ (releasing trifluoroacetic acid) was observed to give an increase in *ee*. A serendipitous discovery revealed that adding the Rh complex in a form in which the axial sites are ligated with pyridine was also found to give a small but consistent boost to enantioselectivity (Figure 37b). By combining these optimization findings, very high enantioselectivity in the aziridination could be obtained for three different chain lengths of styrenyl alkenyl alcohols (Figure 37c). As before, the authors proposed that the substrate hydroxyl engages in hydrogen bonding with the sulfonate of the ligand, which is also ion-paired with the chiral cation tocreate a highly organized chiral pocket for nitrene transfer (Figure 37d). The authors also carried out a systematic study to examine the compatibility of nonstyrenyl homoallylic alcohols bearing various degrees of alkyl substitution. This showed that disubstituted olefins, trans-dialkyl and exodialkyl homoallylic alcohols delivered excellent results, as did one class of trisubstituted olefins (Figure 37e). Based on the findings of this systematic study, the authors proposed a mnemonic to assist users in determining which alkenyl alcohols may give good outcomes.

**Figure 38:**
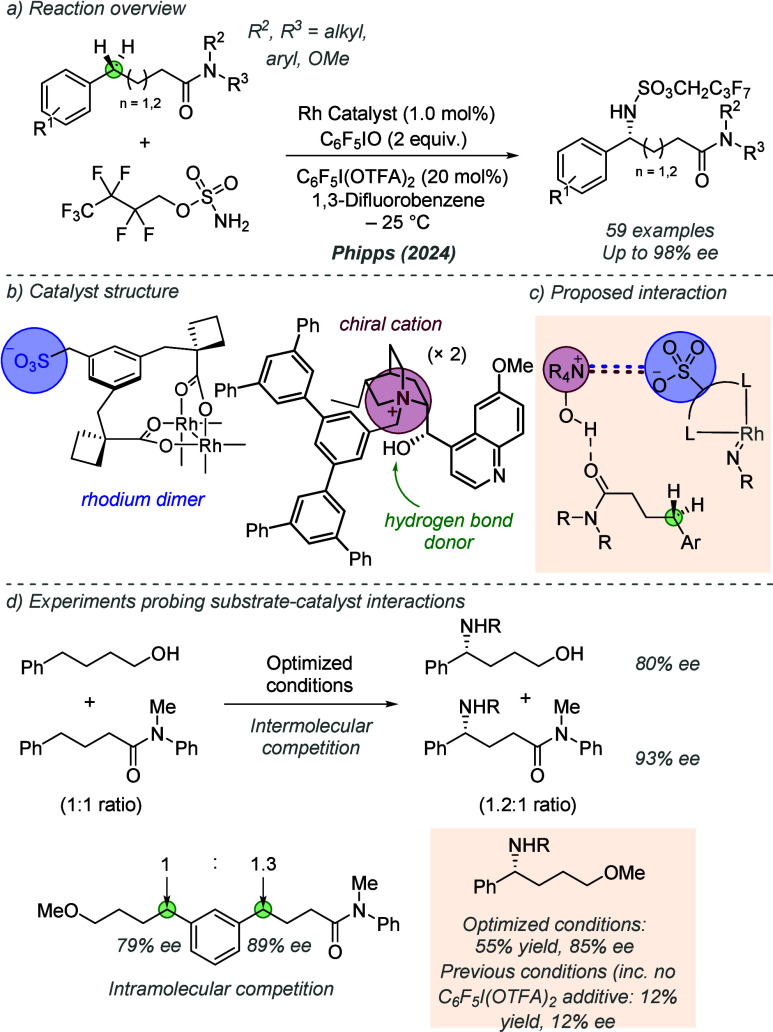
Enantioselective C–H amination directed by tertiary amide-containing substrates.

The same authors subsequently extended the C–H amination reaction to substrates bearing a tertiary amide directing group rather than an alcohol (Figure 38a).^102^ Very similar ion-paired catalysts were found to be optimal (Figure 38b). In contrast with the previous work, these substrates now lack a hydrogen bond donor, suggesting that a different mode of interaction between substrate and catalyst may be operative in this case. The authors proposed that the hydroxyl group of the chiral cation may be acting as a hydrogen bond donor, with the tertiary amide of the substrate constituting the corresponding acceptor. This interaction can then direct the rhodium nitrenoid to one of the two enantiotopic benzylic protons in the chiral environment provided by the cinchona alkaloid-derived cation (Figure 38c). In this reaction, the use of 20 mol % of C_6_F_5_I(OTFA)_2_ (releasing trifluoroacetic acid) as an additive was found to have a significant favorable effect on enantioselectivity. Very broad tolerance of substitution on the arene and amide was exhibited, and two different chain lengths gave excellent results: aryl butyric acid-derivatives and aryl valeric acid derivatives. Various competition experiments were carried out to evaluate the relative effectiveness of alcohol and amide directing groups. Under the optimized conditions, these appear to have similar directing capability (Figure 38d, upper). It was also discovered that even methyl ethers can be effective directors (Figure 38d, lower left and right). Interestingly, in the absence of C_6_F_5_I(OTFA)_2_ (as in the initial 2021 report), these gave very poor outcomes. The authors proposed that inclusion of the TFA-releasing additive allows substates bearing hydrogen bond acceptor groups to interact productively with the cation, and that the acid may change the conformation of the chiral pocket in these cases to enable superior enantioselectivities.

**Figure 39:**
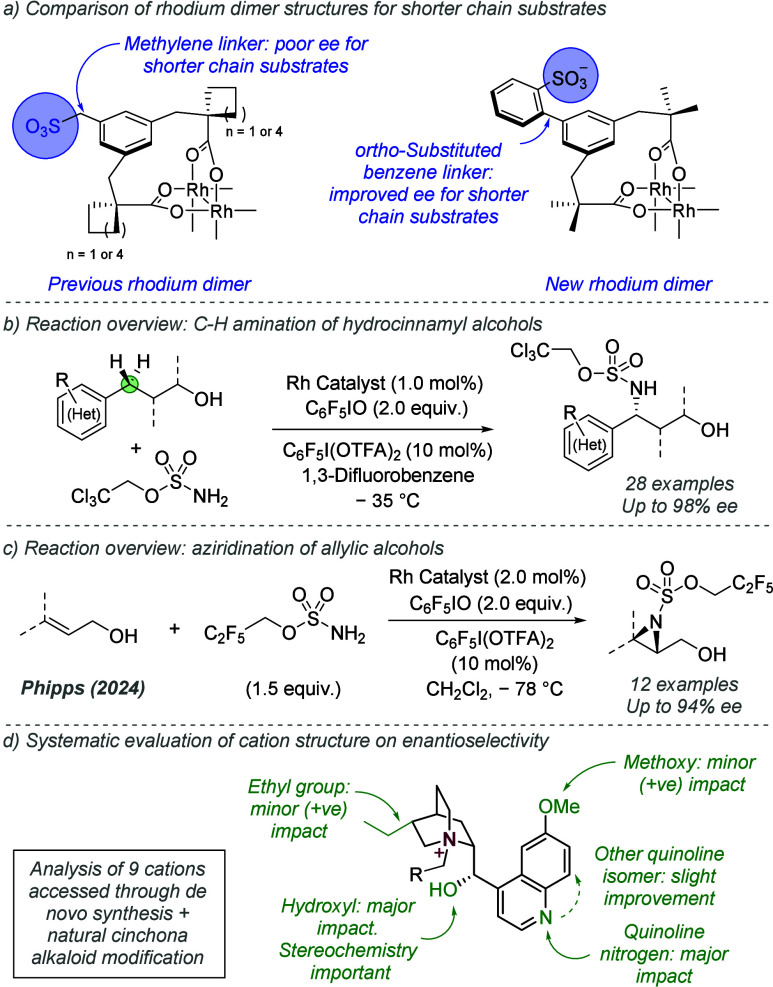
Extension of C–H amination and aziridination to shorter chain substrates and a systematic evaluation of the chiral cation structure.

For the above primary alcohol-directed C–H amination and aziridination reactions, the shortest chain lengths (hydrocinnamyl alcohols and allylic alcohols respectively) gave significantly poorer enantioselectivity under the previously developed reaction conditions (Figure 36 and Figure 37).^100,101^ This represented a significant limitation of the methodology because γ-amino alcohols (from amination of hydrocinnamyl alcohols) can be oxidized to form synthetically valuable β-amino acids, while the aziridination of allylic alcohols is the nitrogen transfer analogue of the Sharpless Asymmetric Epoxidation. In a recent report, Phipps and co-workers overcame these limitations through refinement of the reaction conditions and catalyst structure.^103^ It was hypothesized that reducing the distance between the sulfonate group on the catalyst and the Rh metal center would better accommodate shorter chain lengths–this was achieved by replacing the previously used methylene linker with an *ortho*-substituted benzene linker (Figure 39a). This modification, combined with use of the C_6_F_5_I(OTFA)_2_ additive and pyridine-ligated Rh complex (*vide supra*), allowed C–H amination of hydrocinnamyl alcohols with excellent enantioselectivities and a broad scope (Figure 39b). Meanwhile, prenol-type allylic alcohols were converted to the corresponding aziridines in high *ee* using the same new catalyst design (Figure 39c). In this study, the authors also probed the various structural features of the cinchona alkaloid-derived chiral cation in a systematic “knockout” study, to try to establish the features responsible for its effectiveness as a chiral cation in these reactions (Figure 39d). Nine cations, accessed through a combination of *de novo* synthesis and the modification of naturally occurring cinchona alkaloids, were compared side-by-side in the amination of hydrocinnamyl alcohol. Two of these had the quinoline nitrogen replaced with CH and one featured the quinoline nitrogen moved to the adjacent ring of the quinoline. The ethyl group on the quinuclidine core and methoxy substituent on the quinoline were found to have a very minor effect on *ee*. Intriguingly, the impact of the quinoline nitrogen was dramatic: very poor enantioselectivities were obtained in its absence and once it was reintroduced, irrespective of position within the quinoline, *ee* was returned to excellent levels. The free hydroxyl also proved a key structural feature, with poor enantioselectivity obtained if it was methylated and inferior selectivity if its naturally occurring stereochemistry within the alkaloid was inverted. The precise role played by the quinoline nitrogen remains unclear, but the authors hypothesize that it could be acting as a base and is partially protonated by the acidic additive, leading to a more general catalyst through variation of its conformation.

### 2.3 Ligand Is Neutral but Anion Binding

A different design strategy that incorporates ionic interactions into the outer sphere of transition metal complexes involves the ligand containing a functional group, such as a urea or thiourea, with the intention that it may engage in binding with the anion of a suitable ionic substrate. Therefore, one might engineer an ionic interaction between substrate and catalyst by virtue of the substrate’s anion being abstracted and bound. The concept of anion binding catalysis has been introduced and extensively developed by Jacobsen, with most cases not involving metals.^7c^ In this section, we will deal with examples where this concept has been applied in the outer ligand sphere. In addition there are several examples, covered in Section 3.1, where this approach is applied to abstract an anionic ligand to give a cationic metal center.

#### 2.3.1 Rhodium

**Figure 40:**
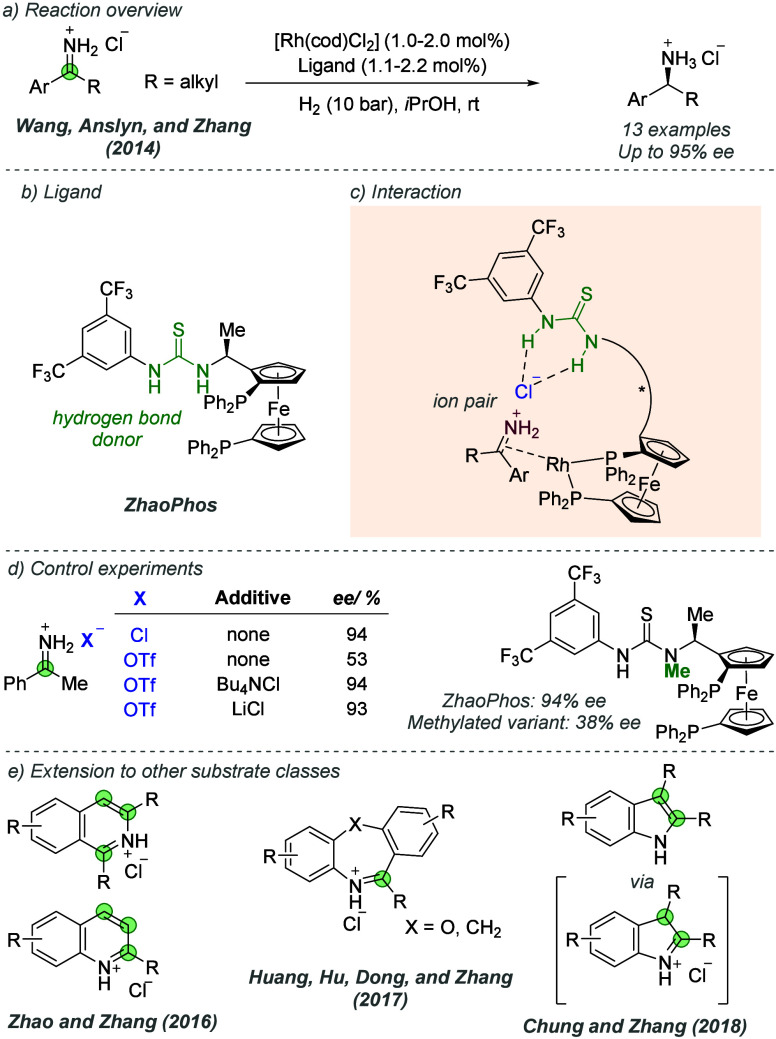
Enantioselective hydrogenation of iminium chlorides with a thiourea-containing bisphosphine ligand.

Initially reported by Wang, Anslyn and Zhang in 2014, several benzylic iminium chlorides underwent enantioselective reduction using a rhodium catalyst to afford benzylic ammonium chlorides with excellent enantioselectivities (Figure 40a).^104^ The ligand for rhodium, which subsequently became known as ZhaoPhos, contained both point and planar chiral elements, and could function as a hydrogen bond donor through its thiourea group (Figure 40b). The proposed interaction involved the ligand hydrogen bonding to a chloride anion of the substrate, which exists as an ion pair with the prochiral iminium species, controlling the facial selectivity of the hydride delivery (Figure 40c). Seeking evidence for the proposed interaction, the authors found that replacing the chloride anion with a triflate resulted in much poorer enantioselectivity (Figure 40d, left). However, enantioselectivity could be restored through the addition of an appropriate chloride-containing additive, such as Bu_4_NCl or LiCl. A thiourea ligand in which one of the hydrogen bond-donating nitrogen atoms was methylated also afforded worse enantioselectivity, consistent with the proposed model (Figure 40d, right). Subsequent reports have extended rhodium-catalyzed hydrogenation using ZhaoPhos to several unsaturated nitrogen heterocycles (Figure 40e). In 2016, Zhao, Zhang, and co-workers reported a reduction of both double bonds in quinolines and isoquinolines to afford the corresponding tetrahydroquinolines and tetrahydroisoquinolines.^105^ Huang, Hu, Dong, and Zhang reported a reduction of 7-membered cyclic iminium substrates in 2017.^106^ Finally, Chung and Zhang disclosed a reduction of indoles in 2018, in which the ion-paired iminium substrate is formed upon addition of HCl.^107^

#### 2.3.2 Iridium

**Figure 41:**
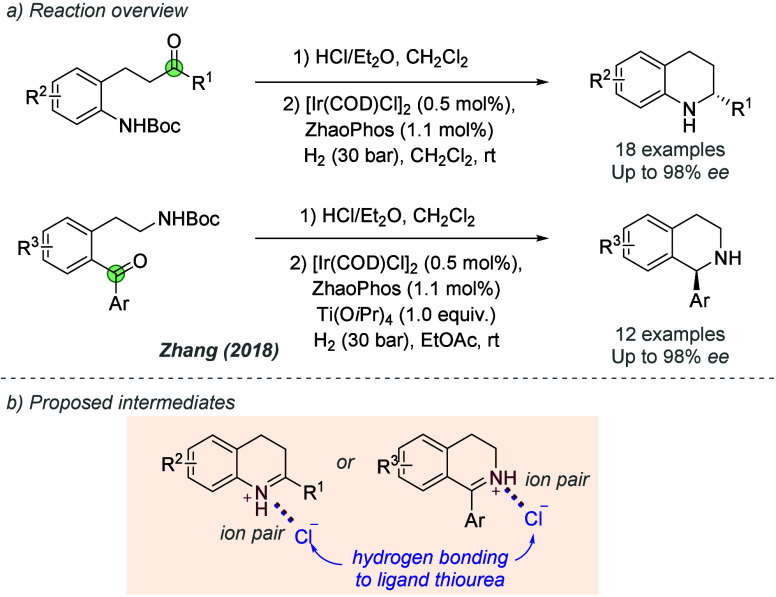
Enantioselective reductive amination with ZhaoPhos to afford chiral tetrahydroquinolines and tetrahydroisoquinolines.

Several reports have emerged using ZhaoPhos as a chiral ligand for iridium as well as rhodium, with an analogous substrate–ligand interaction involving abstraction by the catalyst of the anion from the substrate. In 2018, Zhang and co-workers reported an intramolecular enantioselective reductive amination of prochiral ketones with Boc-protected amines.^108^ Two substrate classes were investigated, affording either a chiral tetrahydroquinoline (Figure 41a, top) or tetrahydroisoquinoline (Figure 41a, bottom), with excellent enantioselectivities obtained. The reaction was conducted in two stages. First, addition of hydrochloric acid deprotected the amine, triggering a cyclization to afford an iminium salt with a chloride counteranion (Figure 41b). Next, the crude intermediate was subjected to enantioselective Ir-catalyzed hydrogenation conditions, whereby the chloride anion was proposed to engage in a hydrogen bonding interaction with the chiral ligand, ZhaoPhos. Thus, the ionic interaction between the catalyst-bound chloride anion and the cationic substrate is considered to be an integral component of the transition state.

**Figure 42:**
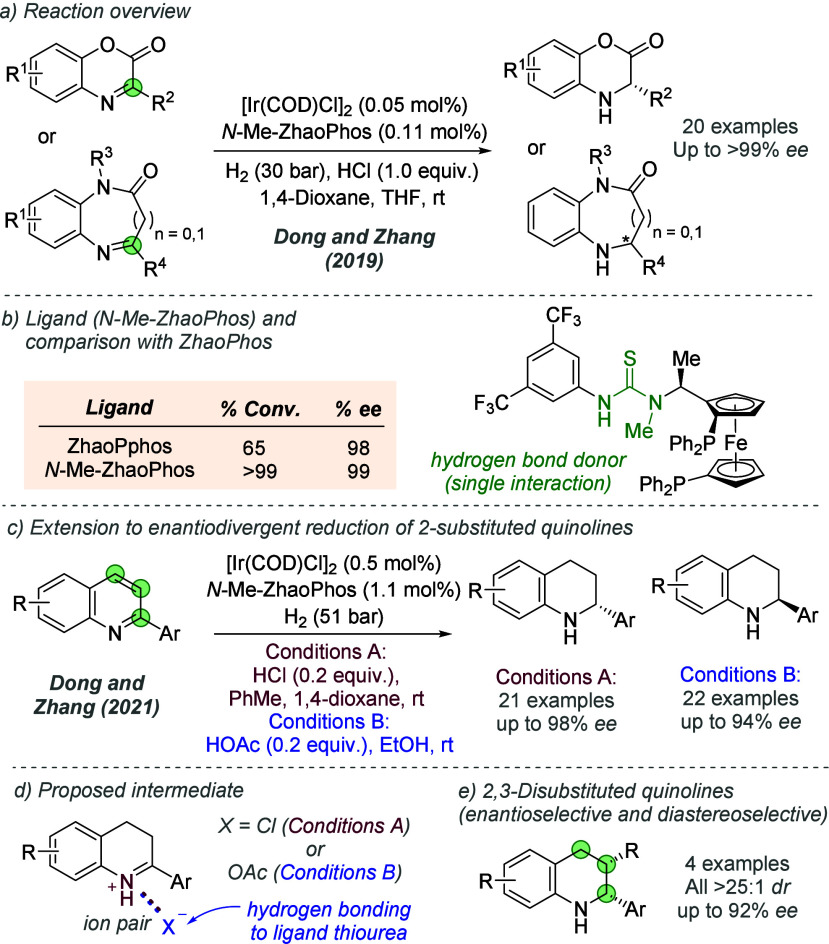
Enantioselective reduction of unsaturated nitrogen heterocycles with *N*-Me-ZhaoPhos.

In 2019, Dong and Zhang reported an enantioselective reduction of benzoxazinones and related heterocycles, with a similar catalytic system (Figure 42a).^109^ Rather than subjecting a preformed ionic substrate to the hydrogenation, this was formed *in situ* in the reaction by the addition of HCl. Interestingly, best results were obtained with a variant of ZhaoPhos, in which one of the hydrogen bond donor groups of the thiourea was methylated (*N*-Me-ZhaoPhos, Figure 42b). While Zhaophos was proposed to interact through two hydrogen bonds, *N*-Me-ZhaoPhos is capable of just one hydrogen bonding interaction with the chloride anion. A comparison of both ligands under otherwise identical conditions revealed significantly higher conversion and marginally improved enantioselectivity for the latter. The same authors extended this to an enantioselective reduction of 2-substituted quinolines using the same ligand (Figure 42c).^110^ In this work, the authors uncovered a surprising dependence of the absolute configuration of the product on the solvent: solvents of lower polarity, including CH_2_Cl_2_, toluene, and ethyl acetate, favored one enantiomer (the (*R*)-configured product), whereas more polar solvents, such as alcohols, favored the opposite enantiomer. The reaction conditions were separately optimized for the formation of each enantiomer (Conditions A and B), although the final optimized conditions differed only in the solvent and acid additive, delivering either enantiomer in excellent enantioselectivities. Although the reasons for this divergence were not elucidated, the authors carried out several control experiments which suggested that the carbon–carbon double bond was reduced first, forming a partially unsaturated ion-paired iminium intermediate (Figure 42d). The anion, which differs between both sets of reaction conditions, was proposed to form a single hydrogen bond to the catalyst. 2,3-Disubstituted quinolines also performed well in the reaction for one enantiomeric series, with excellent diastereoselectivity and enantioselectivity for four examples (Figure 42e).

**Figure 43:**
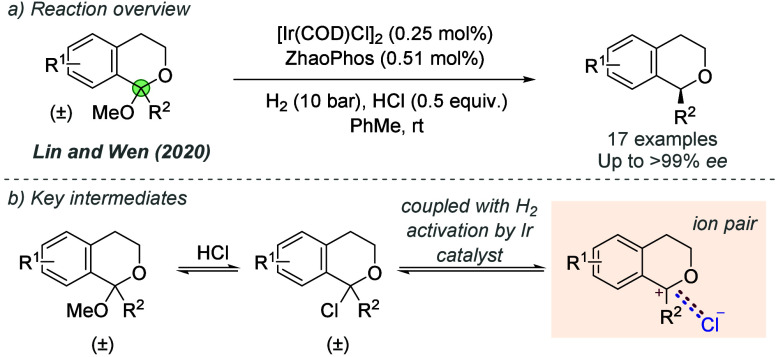
Enantioselective reduction of masked oxocarbenium ions to form isochromanes with ZhaoPhos.

Moving away from nitrogen heterocycles, Lin, Wen and co-workers applied ZhaoPhos to an enantioselective synthesis of isochromanes from oxocarbenium ions, which were introduced into the reaction as racemic methoxy-substituted isochromanes (Figure 43a).^111^ Under the reaction conditions, which include a catalytic HCl additive, the substrate can undergo reversible conversion to the corresponding chloro- intermediate (Figure 43b, left). A chloride anion can then be reversibly abstracted to afford an ion-paired oxycarbenium (Figure 43b, right). DFT calculations revealed a lowest energy pathway in which the C–Cl bond cleavage and activation of H_2_ by the iridium catalyst occurred in a single step. As before, the chloride anion is thought to engage in hydrogen bonding interactions with the thiourea of ZhaoPhos to enable selective delivery of the hydride.

## 3 Ionic Interaction Between Charged Metal and Counterion

In the preceding section, the ionic interaction invoked in each transformation occurred in the outer sphere of the metal complex. We will now consider the situation in which the transition metal itself is proposed to carry a formal charge, and is associated with a counterion of opposite charge, with the resulting ionic interaction leveraged to control some form of selectivity in the reaction. There are two scenarios in which this may occur: the metal can be either cationic, bearing a positive charge or anionic, bearing a negative charge. More examples of the former exist due to the greater prevalence of cationic transition metal complexes in catalytic cycles, but instructive and powerful examples of the latter have also been developed. These two scenarios will be considered in turn.

### 3.1 Cationic Metal Complexes with a Counteranion

**Figure 44:**
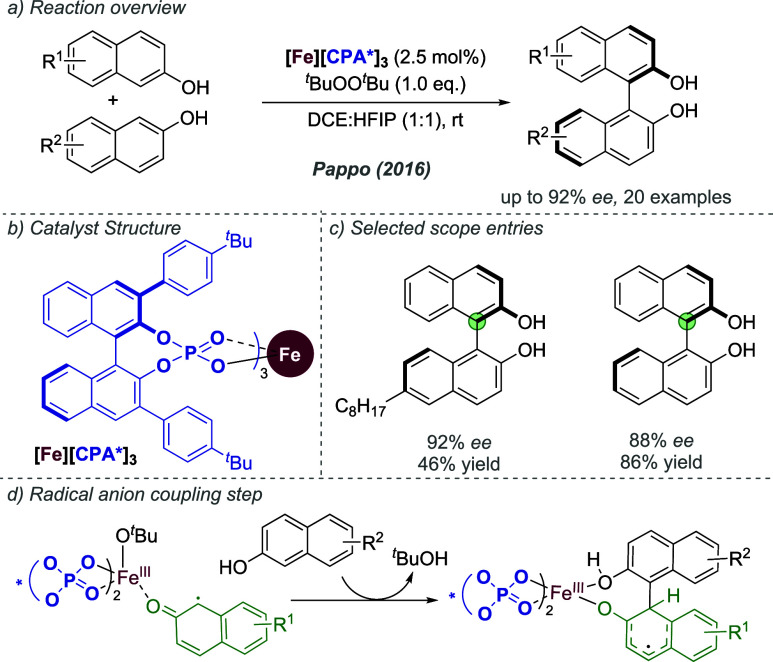
Enantioselective Fe-catalyzed coupling of substituted 2-naphthols using an Fe(phosphate)_3_ catalyst in which mechanistic studies suggest that the phosphates remain bound as ligands throughout.

As mentioned above, it is relatively common to encounter catalytically active transition metal complexes in which the metal is proposed to carry a formal positive charge. This is attractive from a system-design angle, since one can now imagine invoking ionic interactions with a chiral counteranion, or even a charged substrate, to control enantioselectivity or site-selectivity in the ensuing chemistry. Although undoubtedly a valuable design principle, attention must be drawn to the well-acknowledged ambiguities that arise, which relate to whether this constitutes a “true” anion or whether it is coordinated to the metal through a dative covalent bond that would be typical of a classical ligand (and therefore not a “true” counteranion). The reality is a continuum: at either end there are extremes in which the nature of this bonding can be confidently understood, either through theoretical rationalization or detailed mechanistic study. There are also instances where ionic interactions seem plausible based on established understanding of a particular reaction type (for example, the reactive π-allyl palladium intermediates being formally cationic), even if there is no definitive evidence for this in a given reaction. But in many cases, it is difficult to establish definitively whether ionic interactions are involved, or the “anion” is directly ligated, constituting a formally neutral complex. This ambiguity in no way prevents the invocation of ionic interactions from being a useful design principle. But it can make categorizing methods, such as in a Review like this one, challenging when the precise nature of the bonding in the reactive complex is not elucidated. To illustrate this point, we discuss two scenarios that can be considered the “extremes” of this continuum that both utilize chiral phosphates as counterions/ligands. One of the earliest examples of using chiral phosphates in combination with transition metals was from the Toste and co-workers, which combined these with cationic gold(I) complexes. Catalysis using cationic gold(I) had, at that time, been undergoing rapid development and typically involved phosphine-ligated complexes.^112^ Gold(I) was established to have a linear coordination geometry with two available sites for ligation, making asymmetric catalysis using a chiral phosphine difficult due to the distance of the ligand-located chirality from the bound substrate.^113^ Toste and co-workers hypothesized that the cationic nature of the active gold(I) catalyst could be leveraged to provide an extra means for introducing chiral information–through ionic interactions with a chiral phosphate. This transformation will be discussed in more detail below, in section 3.1.1. But the key point is that if one coordination site is occupied by a phosphine and the other is occupied by the bound substrate during activation, then there remain no vacant coordination sites for ligation of the phosphate. Therefore, its role as an anion, associated with the metal complex primarily through ionic interactions, is clear. One example that illustrates the other case involves the work of Pappo and co-workers using iron phosphate and iron bis-sulfonate complexes for the enantioselective, oxidative coupling of 2-naphthols and/or 2-amino-naphthalenes.^114^ To use the first report as an example, in 2016 Pappo and co-workers reported the enantioselective oxidative homocoupling and cross-coupling of 2-naphthols, catalyzed by chiral iron phosphate complexes (Figure 44a,b).^114a^ Optimization resulted in Fe(ClO_4_)_3_ as the iron source, *t*-butyl peroxide and a BINOL-derived phosphate, which together homocoupled 2-naphthol in 74% *ee* (not shown). Preparation of a discrete Fe(phosphate)_3_ complex enabled an increase to 88% *ee* (Figure 44c) for the optimization substrate and cross-coupling could be achieved using partners with sufficiently different oxidation potentials. Throughout this work the authors carried out detailed mechanistic studies using a variety of analytical techniques to come up with a clear mechanistic proposal (Figure 44d). They concluded that the phosphates remain ligated to the iron metal centers as anionic ligands throughout the transformation, according to the mechanism shown which involves a radical anion coupling step between an electrophilic naphthoxyl radical ligand bound to the Fe, and a 2-naphtholate.

Although these two examples are very well-defined, most examples of transition metal catalysis using anions such as phosphates lie between these two, in that the nature of the bonding has not, or perhaps cannot, be rigorously defined. Even in cases where there is an X-ray structure of an intermediate which contains covalently bound anionic ligands, there is always the caveat that this might not reflect the reactive complex in solution. As this Review focuses on the use of ion-pairing as a strategy for designing catalytic systems, we will survey examples where the author proposes or implies that the metal is cationic during the selectivity-determining step and that an ionic interaction is important, even if definitive evidence is not provided. We adopt this approach because we believe that the design principle centered on chiral anions is a compelling one and can lead to creative hypotheses, even if it subsequently transpires that direct ligation of the chiral anion may be occurring. Organic chemistry development, and in particular catalyst design, is a framework-led process: new ways of thinking can help drive organic chemistry development, even in the presence of mechanistic uncertainty.

#### 3.1.1 Gold

Asymmetric catalysis using gold complexes has, in the last two decades, developed very rapidly and remains an active research area. Since stereocenters are formed in many of these transformations, researchers have focused on inducing asymmetry from the earliest efforts. A challenge that became apparent was the linear coordination geometry typically possessed by gold(I) complexes, the most widely utilized. In such instances, the stereochemical information on typical chiral ligands, such as phosphines, may be some distance from the substrate bound at the other coordination site (see previous discussion). As can be seen from the examples below, ion-pairing has proved to be a powerful strategy indeed for rendering gold catalysis enantioselective.

**Figure 45:**
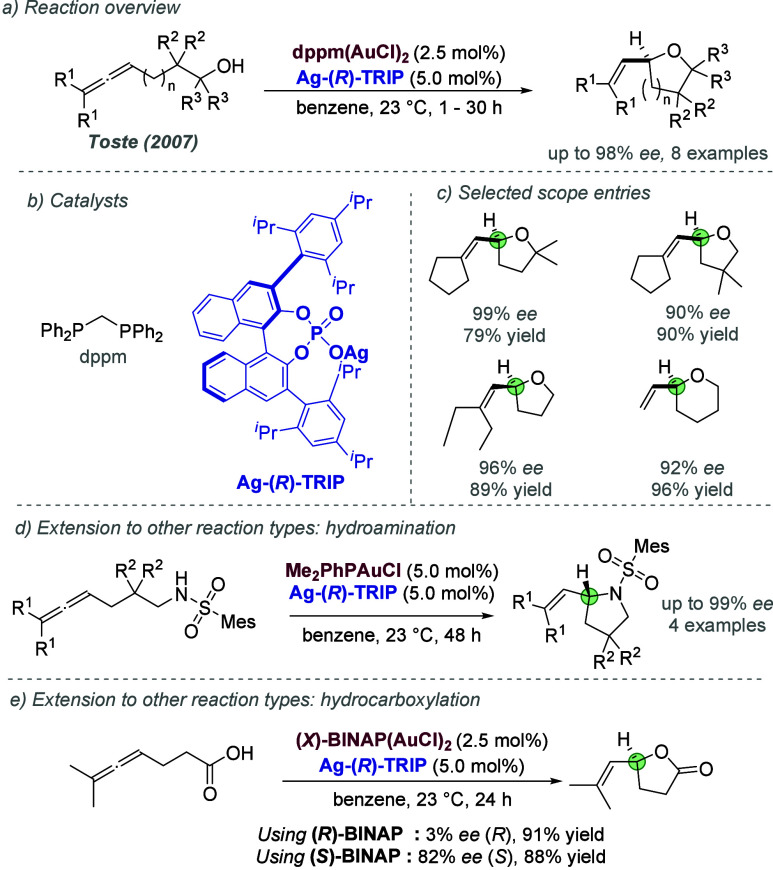
Asymmetric hydroalkoxylation, hydroamination, and hydrocarboxylation using a chiral anion strategy for gold(I)-complexes.

Recognizing the opportunity presented by the fact that many reactive gold complexes are cationic, Toste and co-workers in 2007 described an innovative strategy in which a cationic gold(I)-complex is ion-paired with a chiral BINOL-derived phosphate to enable a highly enantioselective intramolecular hydroalkoxylation of allenes (Figure 45a).^115^ The same authors had previously reported an intramolecular hydroamination of allenes using chiral bisphosphine ligands such as BINAP and SEGPHOS, proposed to form dinuclear gold-phosphine complexes, in which electronic tuning of the associated benzoate anions by inclusion of nitro groups was found to significantly impact *ee*.^116^ When attempting to extend this reaction to allene hydroalkoxylation, it was found that those, and related complexes based on chiral phosphines gave poor outcomes. Given the pronounced effect on *ee* of the achiral anions in the previous work, the authors hypothesized that chiral anions could be used in their place. At least one gold center of the dinuclear complex was anticipated to remain cationic throughout the catalytic cycle, and this could allow the chiral counteranion(s) to remain in close proximity, providing a defined chiral environment in which the reaction can take place. A dinuclear gold complex containing the achiral, bidentate phosphine dppm delivered excellent results when used in combination with two relative equivalents of Ag-(*R*)-TRIP (Figure 45a and b). Nonpolar solvents were essential for obtaining high *ee*, providing support for the importance of ion-pairing. Various allenol substrates were evaluated, varying the allene terminus and introducing substituents α and β to the alcohol, with high levels of enantioselectivity obtained in the tetrahydrofuran products (Figure 45c). The authors also demonstrated the strategy was effective for hydroamination of allenes with sulfonamide nucleophiles, and showed that some substrates which performed poorly under the previously reported catalyst system (chiral bisphosphine only, no chiral anion) were much improved (for example, those in Figure 45d). Additionally, they demonstrated the viability of hydrocarboxylation using carboxylic acid nucleophiles (Figure 45e). In this case, chirality located solely on either the ligand or the counterion performed poorly, but the combination of (*S*)-BINAP with a chiral phosphate produced the product in 82% *ee* with a clear matched/mismatched effect. This demonstrates how the effect of a conventional chiral ligand can be magnified by a chiral counteranion, providing a unique solution to a challenging problem in asymmetric catalysis.

**Figure 46:**
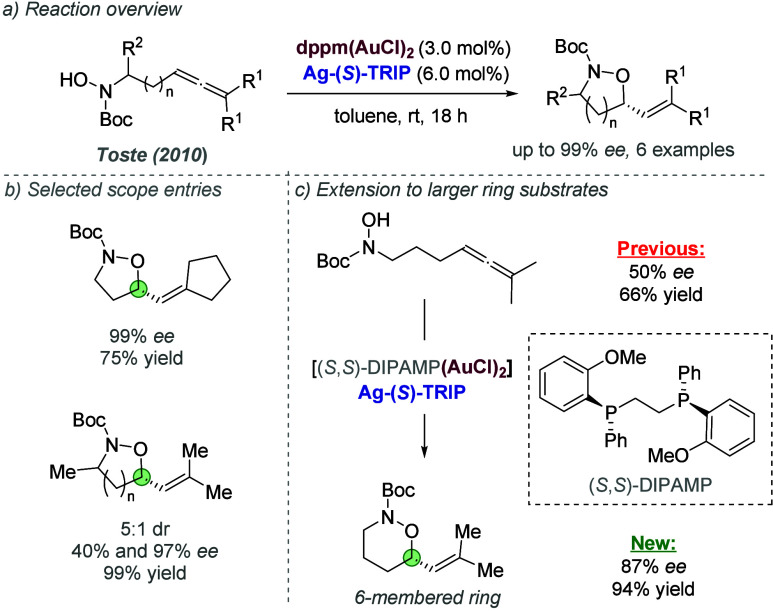
Asymmetric hydroxylation of allenes with hydroxylamine-derived nucleophiles using gold(I)-complexes ion-paired with (*S*)*-*TRIP.

In a further development, also building on the prior work on hydroamination of allenes using dinuclear gold(I) complexes with chiral phosphines,^116^ Toste and co-workers in 2010 reported the enantioselective intramolecular addition of hydrazines and hydroxylamines to allenes, allowing access to chiral isoxazolidines, tetrahydrooxazines, and pyrazolidines (Figure 46a).^117^ High levels of enantioselectivity could be obtained for hydrazine and hydroxylamine hydroamination using conventional BINAP and SEGPHOS-ligated gold(I) complexes in combination with achiral *p*-nitrobenzoate anions. However, for oxygen nucleophiles, specifically hydroalkoxylation to form isoxazolidines, these complexes based on chiral phosphines alone were ineffective–the authors supposed that less coordinating, chiral counterions might provide a solution. They found that by returning to the achiral [dppm(AuCl)_2_] complex combined with two relative equivalents of Ag-(*S*)-TRIP, as in the previous work, excellent *ee* could be obtained in the hydroalkoxylation reaction to form isoxazolidines, allowing the reaction scope to be explored (Figure 46b). When they attempted to form the analogous six-membered ring heterocycle, an oxazine, the *ee* was found to be poorer. This could be improved by combining the silver phosphate with the chiral phosphine DiPAMP (a matched/mismatched effect was observed) – another illustration of the power of combining chiral ligand and chiral anion strategies (Figure 46c).

**Figure 47:**
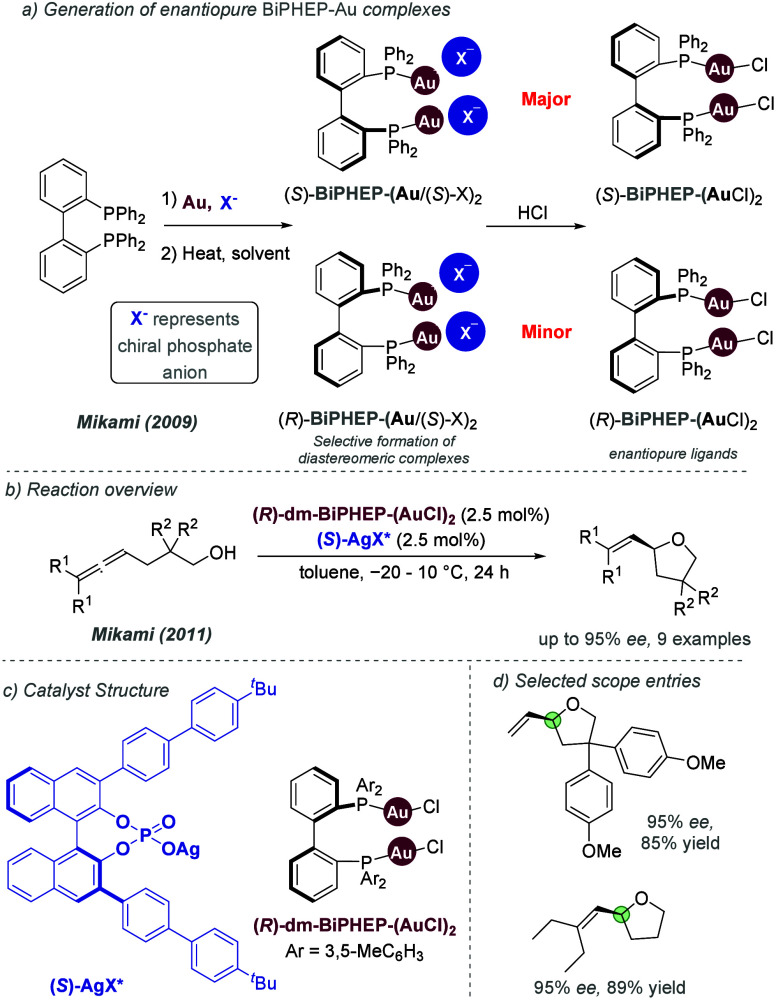
Asymmetric hydroalkoxylation of allenes to give tetrahydrofurans catalyzed by chiral gold(I)-complexes based on dm-BiPHEP ligands ion-paired to chiral silver phosphates.

In 2009 Mikami and co-workers disclosed how the axial chirality of BiPHEP-gold complexes could be controlled via the formation of diastereomeric salts with 3,3′-substituted BINOL-derived phosphates after thermal equilibration.^118^ Isomerization at high temperatures (typically 100 °C) formed the thermodynamically favored (*S*)-BiPHEP/(*S*)-phosphate diastereomeric complexes with extremely high diastereoselectivity, depending on the specific combination. The authors showed that the corresponding (*S*)-BiPHEP-(AuCl)_2_ complex could be reformed in >99% *ee* by treatment with HCl at low temperature (Figure 47a). This complex, upon treatment with silver *para*-nitrobenzoate, was then shown to give high *ee* as a catalyst in intramolecular allene hydroamination, in analogy with the previous work of Toste using more conventional chiral bisphosphines.^116^ Building on that initial study, the same authors subsequently reported the synergistic combination of these enantiopure chiral (*S*)-BiPHEP-(AuCl)_2_ complexes with chiral silver phosphates to take advantage of a chiral counterion strategy.^119^ They optimized this for the intramolecular hydroalkoxylation of allenes using alcohol nucleophiles on a substrate which features no substituents on the far end of the allene (R^1^=H in Figure 47b). This challenging substrate gave poor *ee* outcomes when using the chiral phosphate anion alone, but this improved when a chiral phoshine was used in partnership and the enantiopure BiPHEP-(AuCl)_2_ complexes performed well in this regard. The optimal combination was the silver phosphate salt depicted, together with the 3,5-dimethylsubstituted BiPHEP-(AuCl)_2_ complex shown (Figure 47c). The reaction scope was explored to give a collection of substituted tetrahydrofuran derivatives with up to 95% *ee* (Figure 47d).

So-called "tandem" reactions are more mechanistically complex, so determining whether ionic interactions are at play can be more challenging. For example, in 2009 Gong and co-workers reported a tandem hydroamination followed by asymmetric transfer hydrogenation, using an achiral gold complex and chiral Bro̷nsted acid catalyst system with a Hantzsch ester hydride source.^120^ In this case the chiral phosphoric acid catalysis and gold(I) catalysis are completely independent and so fall outside the scope of this Review. Similarly, Liu and Che in 2009 reported the synthesis of chiral secondary amines by a gold(I)/chiral Bro̷nsted acid tandem hydroamination and transfer hydrogenation reaction.^121^ A series of control experiments revealed that the enantioselective hydrogenation step could be catalyzed by the chiral phosphoric acid additive alone, leading to the conclusion that the gold is not likely to be involved in the selectivity-determining step. In 2011, Gong and co-workers reported an enantioselective gold(I)-catalyzed cyclization of alkynols using an achiral dialkylbiarylphosphine ligand for gold, together with a chiral phosphate anion.^122^ The initial step of this reaction involves gold-catalyzed enol formation followed by rearrangement to an oxonium ion. Following this, there were two possible roles of the chiral phosphate proposed in inducing selectivity: the first involved the gold catalyst in which the phosphate was proposed to act as a chiral anion for gold, and the second in which gold was not involved in the stereodetermining step. Although high *ee* was obtained for the optimization substrate, scope exploration suggested that broadly high selectivity could not be achieved.

**Figure 48:**
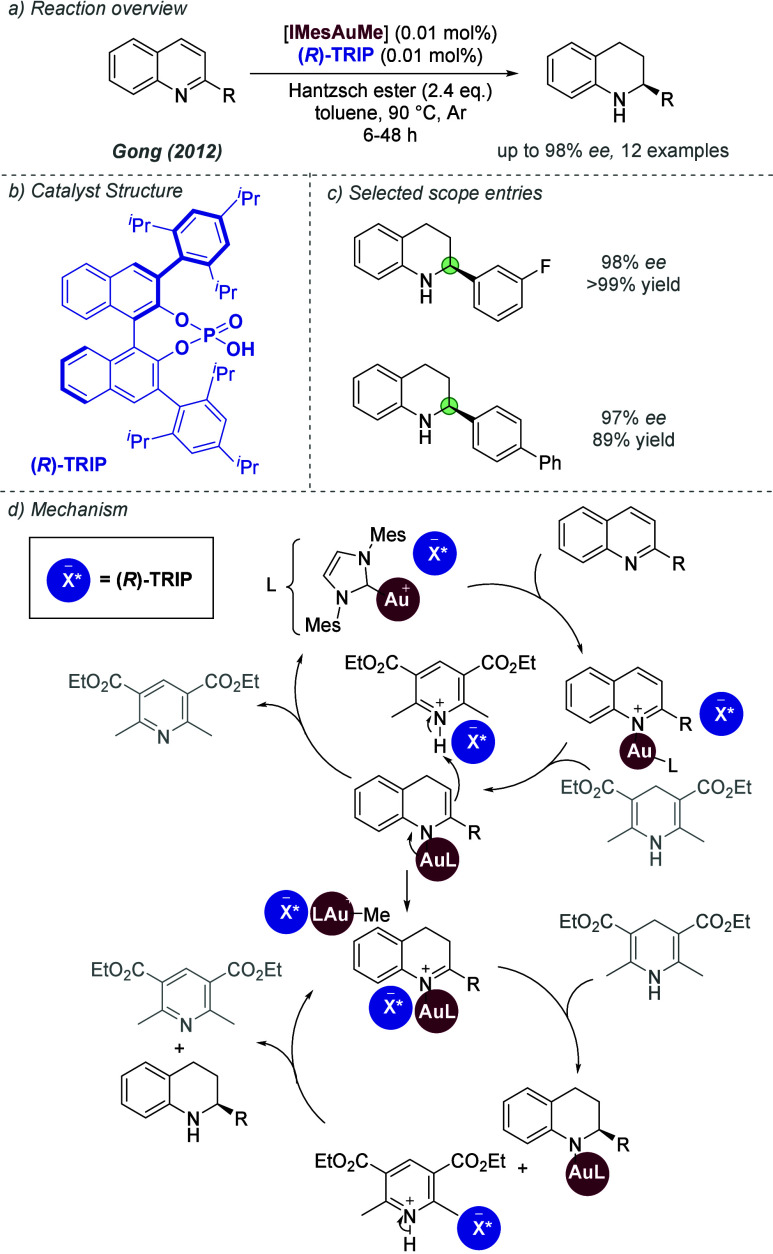
Asymmetric transfer hydrogenation of 2-arylquinolines with a gold(I) carbene complex and phosphoric acid.

In 2012, Tu and Gong reported a double asymmetric transfer hydrogenation of quinolines to give chiral tetrahydroquinolines (Figure 48a).^123^ The achiral NHC IMes was found to be the best ligand for gold, and the reaction was accelerated by addition of a chiral phosphoric acid, with TRIP being optimal for *ee*. Control experiments revealed that the gold carbene precursor complex alone did not promote the reaction. The reaction scope consisted of a variety of 2-arylquinolines; aliphatic groups in the 2-position gave poorer outcomes (Figure 48c). The authors propose a mechanism whereby the gold(I) complex acts as a Lewis acid, coordinating to the nitrogen in the quinoline ring to activate this species toward initial reduction from the Hantzsch ester (Figure 48d, top cycle). This process is proposed to repeat for the second hydrogenation (lower cycle), with the authors suggesting that gold is playing a key role in activating the iminium intermediate toward reduction, with the associated chiral phosphate anion controlling asymmetry. Because it is well established that chiral phosphoric acids can control selectivity in transfer hydrogenations of related substrates, a detailed interpretation of the roles of the phosphate and gold is difficult without deeper mechanistic studies.

**Figure 49:**
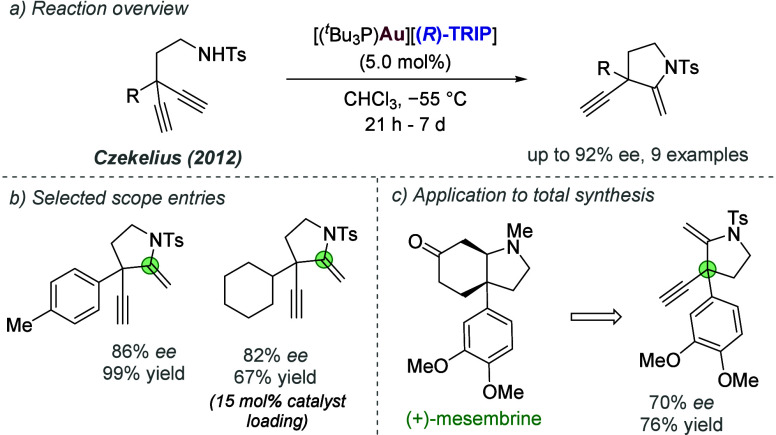
Desymmetrizing cyclization of diynamides to give pyrrolidines catalyzed by achiral gold(I) complexes with a chiral phosphate counteranion.

Czekelius and co-workers in 2012 reported an intramolecular enantioselective cyclization of diynamides to give chiral pyrrolidines (Figure 49a).^124^ They began by demonstrating that conventional chiral phosphine ligands for gold(I) gave very poor enantioselectivity, but good reactivity. They addressed this problem by adopting the chiral counterion strategy pioneered by Toste and found that, even for triphenylphosphine gold complexes, inclusion of TRIP as a chiral anion could deliver *ee* values of up to 56%. Ultimately, (*R*)-TRIP in combination with *t*Bu_3_PAuCl catalyzed the desymmetrization reaction in high enantiomeric excess (up to 92% *ee*); optimization also revealed the importance of a low reaction temperature of −55 °C. While the reaction could tolerate aromatic and aliphatic groups on the same carbon as the two alkynes, for the latter *ee* was substantially reduced, even with higher catalyst loading (e.g., 82% *ee* for Figure 49b, right). The same group subsequently applied this reaction to the total synthesis of (+)-mesembrine to deliver the substituted pyrrolidine core in 76% yield and 70% *ee* (Figure 49c).^125^ Subsequent steps to arrive at the natural product incorporated a recrystallization to increase the enantiomeric excess to >99%.

**Figure 50:**
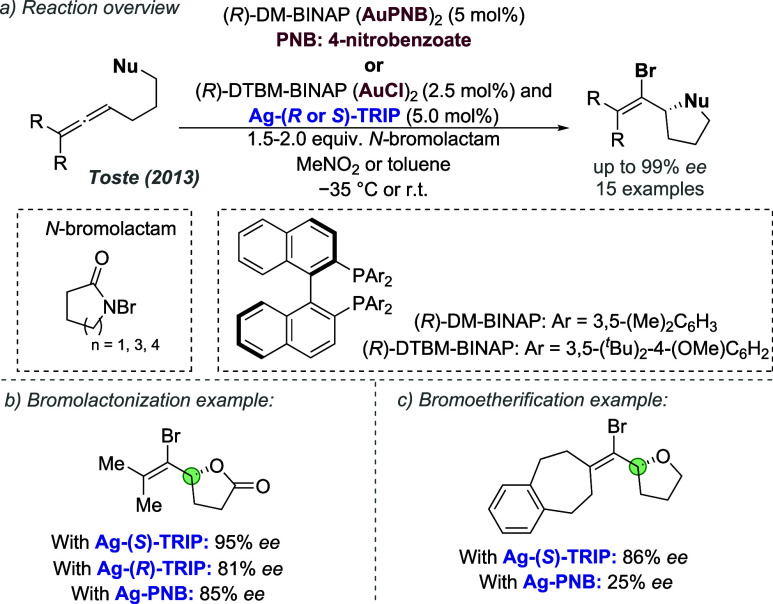
Enantioselective gold(I)-catalyzed bromolactonization and bromoetherification of allenes enabled by a chiral phosphate counterion strategy.

In 2013, Toste and co-workers reported a study which sought to replace the terminating protodeauration step in allene additions with halodeauration, to afford vinyl halide products in an overall enantioselective halocyclization of allenes (Figure 50a).^126^ After some experimentation, *N-*bromolactams proved to be excellent electrophilic bromine sources, giving high yield and *ee* for a hydroamination reaction which formed a chiral pyrrolidine. A dinuclear gold complex bearing a chiral phosphine ligand paired with achiral nitrobenzoate anions was found to give high *ee*, and a broad scope was demonstrated. As in previous studies, changing from a nitrogen to an oxygen nucleophile proved challenging using a complex bearing a chiral ligand alone. As before, using chiral phosphates instead of achiral *para*-nitrobenzoate (PNB) anions was an excellent solution, and allowed high *ee* to be obtained in a demonstration of bromolactonization for one substrate. Here a matched/mismatched effect was observed when using (*S*)-TRIP (95% *ee*) compared with (*R*)-TRIP (81% *ee*), with PNB resulting in an *ee* value between the two (Figure 50b). Bromoetherification could also be accomplished using this chiral anion strategy; for the example substrate depicted in Figure 50c, only 25% *ee* could obtained with an achiral anion. The authors also carried out interesting studies which suggest that the enantiodeterming step can switch between cyclization and proto/bromodeauration, in a delicately balanced mechanistic situation in which the nature of the counterion, as well as other factors, can have deciding influence.

**Figure 51:**
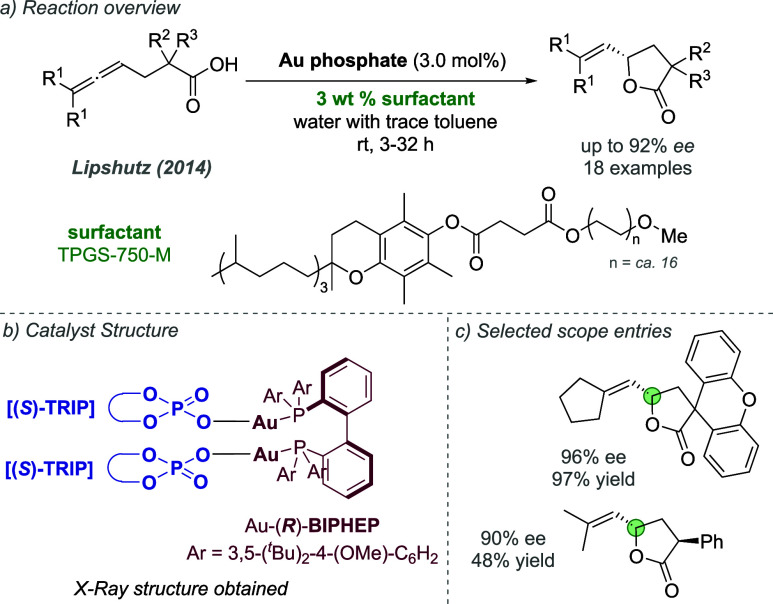
Enantioselective gold(I)-catalyzed lactonizations in aqueous, *in situ*-formed nanomicelles, in which chiral anions were crucial for high *ee*.

Lipshutz and co-workers in 2014 reported an alternative method for performing chiral anion-directed gold catalysis, using micellar catalysis. They hypothesized that the hydrophobic interior of aqueous nanomicelles could provide an ideal apolar environment for tight ion-pairing, forming a well-defined chiral pocket for catalysis.^127^ The hydrocarboxylation of allenic acids was targeted and the surfactant TPGS-750M/H_2_O was used together with 5 mol % (*R*)-BINAP-(AuCl)_2_ and various achiral silver salts (Figure 51a). This afforded the desired lactone product in typically high yield, but in many cases low *ee*, with 55% *ee* being the maximum with an achiral anion. Introduction of a chiral silver phosphate cocatalyst allowed the *ee* to be increased to 75%, with a further improvement to 88% if a preformed gold-phosphate complex was used (Figure 51b). It was found that a drop of organic solvent greatly reduced reaction times–this was proposed to “soften” the highly crystalline nature of the solids being added. A variety of alkyl substitution patterns on the allenic group were tolerated, and the carboxylate α-position tolerated various aryl substituents (Figure 51c). The authors showed that both the surfactant medium and the catalyst could be recycled upon aqueous extraction, and reused without loss of enantioselectivity in six subsequent reactions.

**Figure 52:**
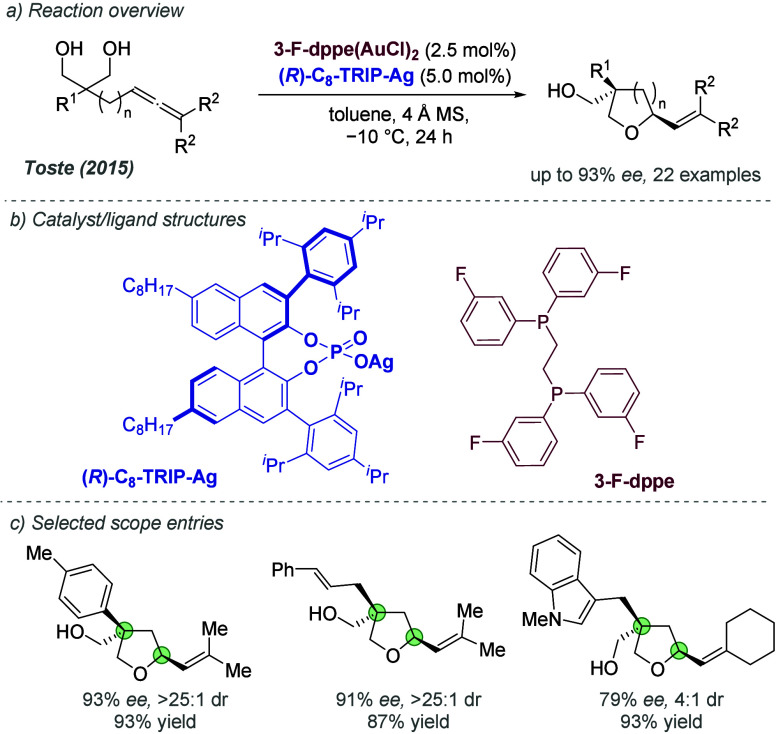
Enantioselective gold(I)-catalyzed desymmetrization of 1,3-diols via intramolecular hydroalkoxylation of allenes, controlled by associated chiral phosphate anions.

In 2015, Zi and Toste reported the enantioselective desymmetrization of 1,3-diols via hydroalkoxylation of allenes, a process which forms at least two stereocenters with typically high diastereoselectivity (Figure 52a).^128^ During optimization, a variety of mono- and bidentate achiral phosphine ligands generated achiral gold complexes that were paired with chiral phosphate counterions. Of the first ligands explored, diphenylphosphinoethane (dppe) paired with TRIP gave 57% *ee* and excellent diastereoselectivity (>25:1 d.r.). Further optimization was achieved by addition of *n*-octyl chains on the TRIP scaffold and addition of fluorine substituents to the dppe (Figure 52b), allowing up to 93% *ee*. A variety of substituents were well tolerated, as was variation at the quaternary stereocenter (Figure 52c). Interestingly, lowering the amount of phosphate to a 2:1 Au:Ag ratio caused a drop in *ee* to 78%, in contrast to the optimal result with 1:1, which gave 93% *ee* (not shown). Despite this, there was no nonlinear effect observed, providing evidence against a second chiral phosphate being closely involved during the enantiodetermining step.

**Figure 53:**
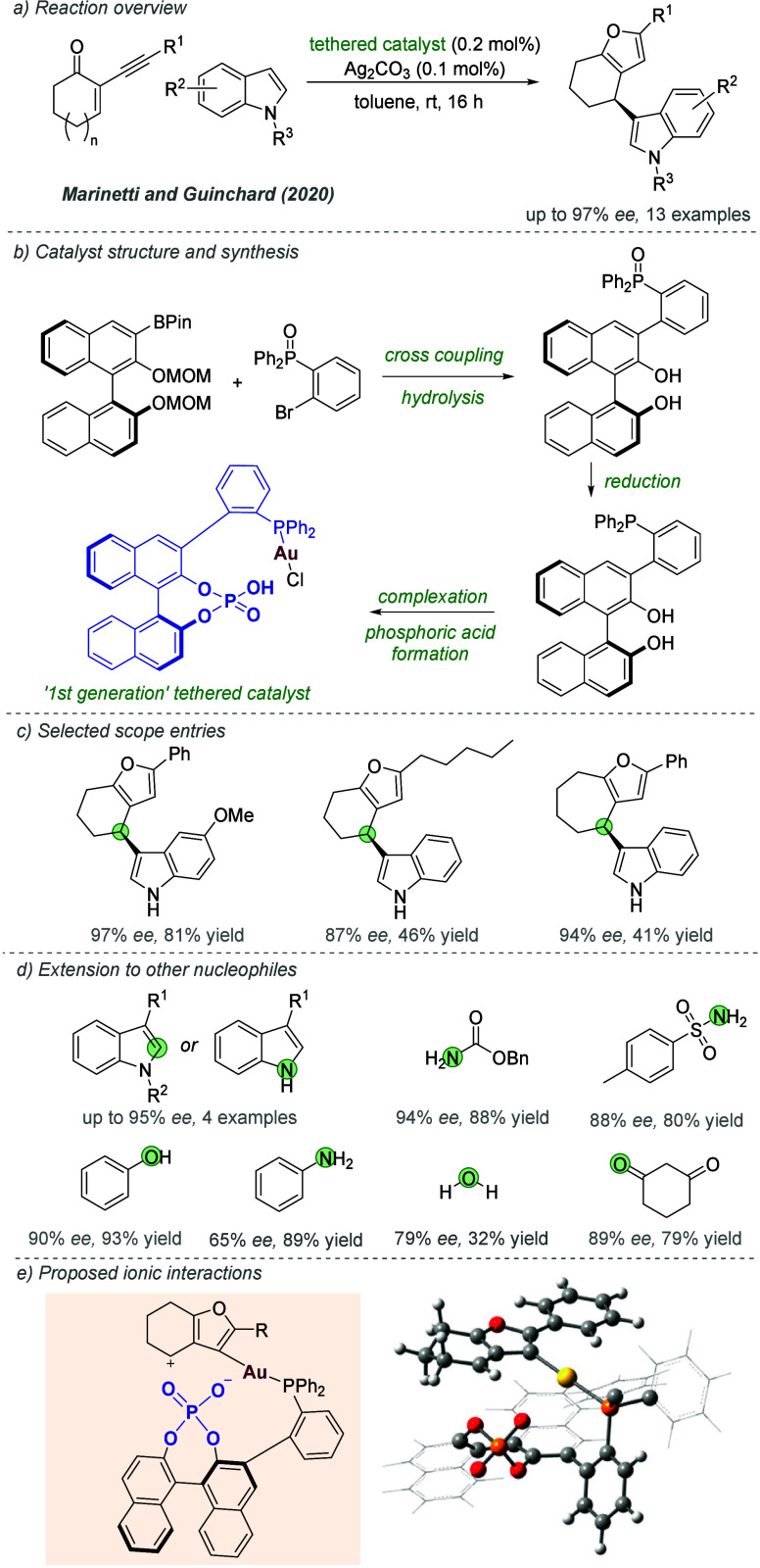
A novel ligand for gold(I) in which the chiral phosphate counterion is covalently tethered to the ligand, applied to a cycloisomerization/indole addition reaction. Panel e shows optimized geometry following DFT calculations, reproduced from ref (129).

In 2020, Marinetti, Guinchard and co-workers disclosed a development of the chiral anion strategy for asymmetric gold(I) catalysis, which they termed ‘tethered counterion-directed catalysis’.^129^ This involves tethering the chiral phosphate anion covalently to an achiral phosphine ligand with the hypothesis that this tether, if properly designed, would restrict the conformational possibilities for the chiral anion when it is ion-pairing with cationic reaction intermediates. This would hopefully allow for optimal positioning, leading to high levels of enantioselectivity. The novel bifunctional ligands were investigated in a tandem cycloisomerization/indole addition process, using 2-(phenylethynyl)-2-cyclohexenones as substrates (Figure 53a). The tethered achiral monophosphine ligand was synthesized starting from BINOL in a relatively short sequence (Figure 53b). Optimization revealed that use of toluene as the solvent gave the highest enantiomeric excess, which was much reduced in more polar MeCN, consistent with ion-pairing being important for enantioselectivity. The gold(I) catalyst loading could be reduced to very low levels (0.2 mol %), alongside 0.1 mol % Ag_2_CO_3_. Various control experiments were carried out, which showed that all aspects of the catalyst structure are important for high *ee*, in line with the original hypothesis. The scope was examined, and initially a variety of substituted indoles were subjected to the reaction (Figure 53c). Blocking the indole 3-position allowed substitution to occur at either nitrogen or the 2-position. The authors then explored different nucleophiles including benzylcarbamate, tosylamide and phenol, all of which could give high levels of enantioselectivity, although water was lower (Figure 53d). The authors also discovered that the silver cocatalyst could be omitted, and excellent results could still be obtained, albeit at a slightly lower reaction rate–a beneficial feature of this catalyst design which is not common in gold catalysis more generally. The proposed mechanism begins with activation of the enone by the cationic gold(I) center, promoting the cycloisomerization step and giving rise to a prochiral carbocation intermediate (not shown). Nucleophilic addition to the carbocation is followed by a protodeauration step, which could be assisted by the phosphate. It is suggested that the enantioselectivity obtained arises from the highly organized, ion-paired intermediate, creating a well-defined chiral environment in which the nucleophile addition can take place (Figure 53e). DFT calculations were carried out which supported this proposal.

**Figure 54:**
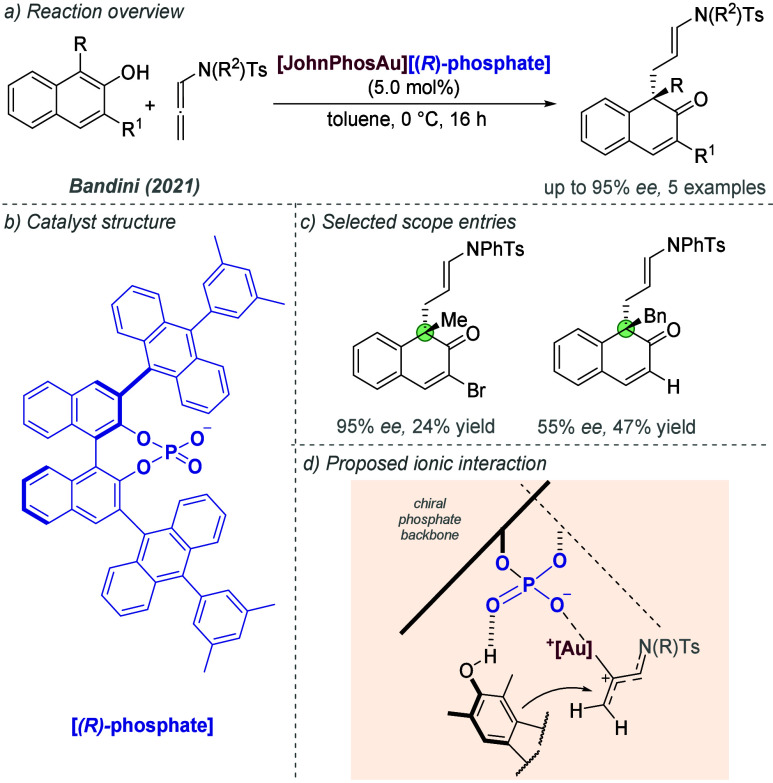
Enantioselective gold(I)-catalyzed dearomatization of 2-naphthols with allenamides using a chiral anion approach.

In 2021, Bandini and co-workers explored the enantioselective gold(I)-catalyzed dearomatization of 2-naphthols at the C-1 position with allenamide electrophiles (Figure 54a).^130^ This work used a chiral anion approach, and optimization began with achiral gold chloride complexes bearing various ligands, combined with chiral phosphates. A chiral phosphate bearing anthracenyl substituents at the 3,3′ positions achieved a maximum *ee* of 83% in combination with a JohnPhos-ligated gold(I) cation (not shown). The authors then synthesized larger chiral phosphates featuring aryl substitution on the anthracenyl groups, extending the chiral pocket (Figure 54b). This allowed enantioselectivity to be increased to 90% *ee* for the optimization substrate and several other 2-naphthols were evaluated in the reaction, giving between 55 and 95% *ee* (Figure 54c). Interestingly, if the gold(I) catalyst was removed and the optimal phosphate used as a Bronsted acid catalyst, moderate yield of the product was still obtained and with 81% *ee*, raising questions regarding the role of gold catalysis in the reaction. The authors attempted to extend the scope with the use of differently *N*-substituted allenamide partners, but these resulted in low selectivities for the two examples disclosed. The authors suggest an enantiodetermining step involving attack of the naphthol onto the cationic allyl gold complex by an outer-sphere mechanism, with hydrogen bonding of the naphthol to the chiral phosphate enabling a highly organized transition state (Figure 54d).

**Figure 55:**
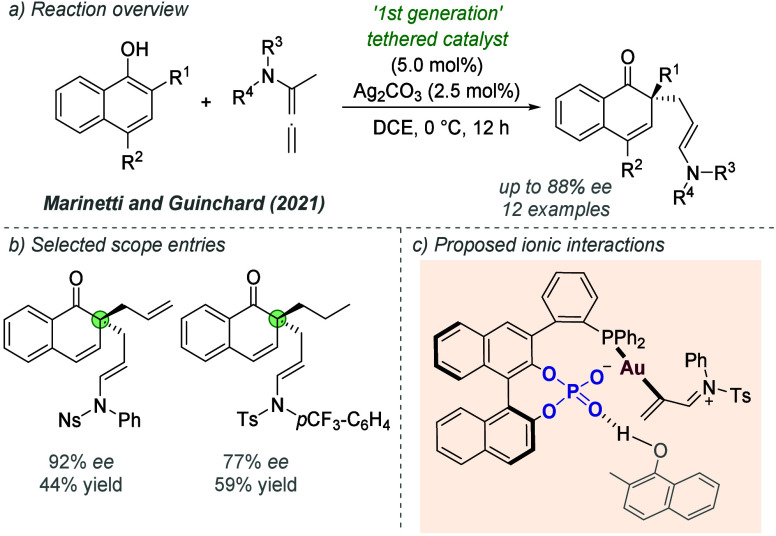
Gold(I)-complex bearing a tethered chiral anion applied to the enantioselective dearomatization of 1-naphthols using allenamides.

Also in 2021, Marinetti, Guinchard and co-workers published a gold(I)-catalyzed dearomatization of 1-naphthols, applying their tethered-counterion approach (Figure 55a).^131^ The authors used the same gold complex as in their previous work, together with Ag_2_CO_3_. This gave high *ee* for dearomatization at the 2-position of a 2-substituted-1-naphthol, when reacted with an *N*-tosyl, *N*-aryl allenamide partner. Substitution on the 1-naphthol included varying degrees of steric bulk at the 2-position, but excessive bulk such as a propyl group dropped the *ee* to 77% (Figure 55b). The authors then extended this to 2-naphthols, as in the above Bandini report, which provided the dearomatized products in good yield but more moderate *ee*. Various control experiments indicated that all aspects of the optimal tethered ligand structure were required for high *ee*. DFT calculations supported the hypothesis outlined for the tethered counterion approach: the 1-naphthol is activated via hydrogen bonding with the phosphate group, which is itself ion-paired with the gold-allenamide adduct (Figure 55c). The calculated transition states agreed with the absolute configuration of the experimentally obtained enantiomer.

**Figure 56:**
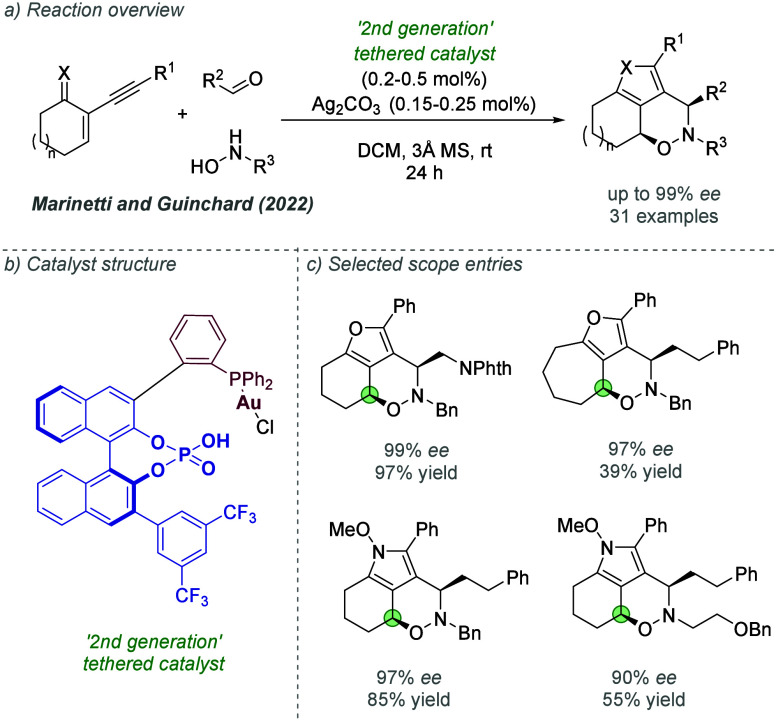
Gold(I)-complex bearing a tethered anion applied to enantioselective multicomponent annulations to give furan and pyrrole oxazines.

In 2022, the same authors reported a gold(I)-catalyzed multicomponent annulation of aldehydes, hydroxylamines and cyclic yne-enones using their tethered counterion ligands (Figure 56a).^132^ Optimization was performed on an yne-enone and a preprepared nitrone, and while their original ligand from previous studies gave excellent results, these could be improved even further by adding a 3,5-bis(trifluoromethyl)phenyl substituent at the remaining 3-position of the BINOL core (Figure 56b). For the scope exploration, nitrones were prepared *in situ* from the corresponding aldehydes and hydroxylamines. Several of these aldehydes and hydroxylamines were investigated, and the yne-enone could also be varied without significant impact on *ee* (Figure 56c, upper). DFT calculations suggested that the origin of selectivity resembled that in previous related cyclizations, only here a nitrone was used as the nucleophile. Ion pairing between the tethered phosphate and the cationic intermediate was again proposed to be key. The authors also extended the methodology to oximes to form *N*-alkoxypyrroles (Figure 56c, lower).

In 2022, Liu and co-workers reported an enantioselective *para*-selective C–H functionalization of alkyl benzenes with diazo compounds, in which a chiral phosphoric acid is used alongside a phosphite-ligated gold(I) catalyst.^133^ Studies suggested that two molecules of TRIP are involved in the enantiodetermining transition state and one of the possibilities is that gold is not involved in the enantiodetermining step, so further studies will be needed to define the role of ion pairing here.

**Figure 57:**
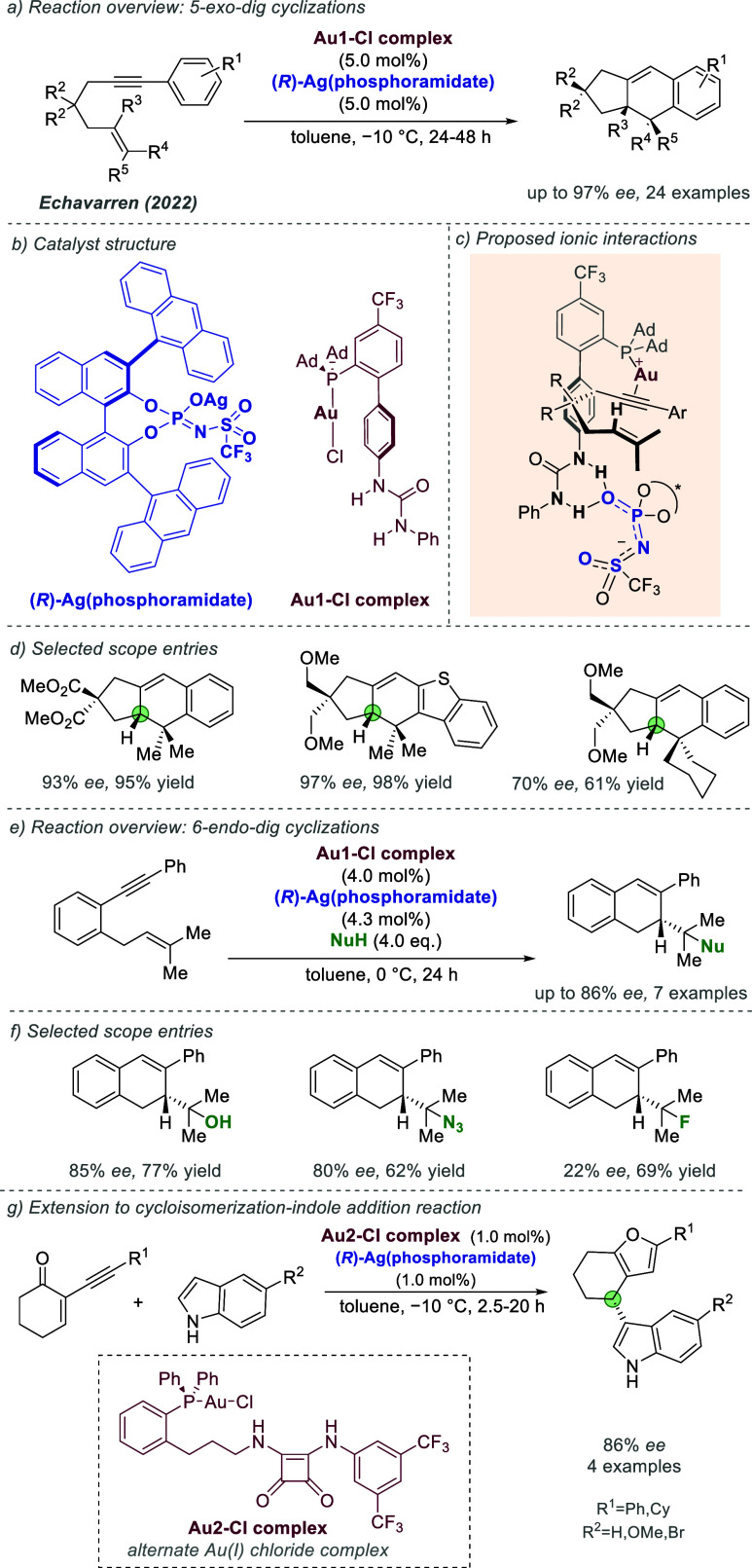
Enantioselective gold(I) cycloisomerization of 1,6-enynes using a bifunctional phosphine ligand, in which the chiral phosphoramidate counteranion is proposed to hydrogen bond to the ligand.

A further innovation in the combination of chiral anion approaches with gold(I) catalysis was accomplished by Echavarren and co-workers in 2022. The authors report a ligand design which permits the associated counteranion to hydrogen bond to a purposely installed functional group on an achiral phosphine (Figure 57b).^134^ They designed Buchwald-type dialkylbiarylphosphines which contained either a urea or thiourea on the lower aryl ring. It was intended that this could bind to the chiral anion, allowing ready formation of the cationic complex while the chiral anion is precisely positioned, with a high level of organization resulting in high enantioselectivity. In prior work which informed this study, the same group had explored ligands for gold(I) which possessed urea- and squaramide groups,^135^ which, through anion binding, permitted abstraction of a chloride ion from the gold center without the need for silver additives. Noting that enantioselective gold catalysis had so far been predominantly applied to allene substrates and not generally translated to reactions of alkynes the authors chose to evaluate their ligands in the cycloisomerization of 1,6-enynes (Figure 57a). A selection of bifunctional phosphine ligands were synthesized and evaluated in combination with various chiral BINOL-derived phosphates and phosphoramidates. No yield was obtained using chiral phosphates, and it was the less coordinating phosphoramidates that gave reactivity as well as encouraging enantioselectivity. Ligand optimization identified a *P*-adamantyl phosphine bearing a CF_3_ substituent and a urea as optimal which, after optimization of solvent and reaction temperature, gave excellent yields and enantioselectivities (Figure 57b). Enyne substrates with various linker substitution and alkyne components were tolerated (Figure 57d). The strategy was also applied to 6-*endo*-dig cyclizations, which are followed by trapping with a nucleophile such as an alcohol, azide or fluoride to give the corresponding cyclized products in good yields and mostly high enantioselectivities (Figure 57e,f). Finally, the catalyst was also shown to give high *ee* in cycloisomerization/indole addition to enynones, in which the indole addition is enantiodetermining (Figure 57g). In this case, a squaramide-bearing phosphine-gold complex was found to be optimal. Extensive mechanistic studies were conducted which revealed no nonlinear effect, while kinetics and NMR-titrations provided evidence for the proposed hydrogen bonding interactions. Control experiments emphasized the importance of the urea for abstraction of the chiral anion from the gold center. The proposed mechanism begins with the phosphoramidate coordinating to gold. Dissociation is promoted by hydrogen bonding with the urea followed by complexation of the alkyne. This sets the stage for the enantiodetermining cyclization in the chiral environment provided by the hydrogen bonded cationic complex (Figure 57c). Based on DFT calculations, the authors proposed that the phosphoryl oxygen of the anion hydrogen bonds with both urea NHs. Following this, cyclization occurs by attack of the *Si* face of the alkene to the activated alkyne, to give a cyclopropyl gold(I) carbene.

**Figure 58:**
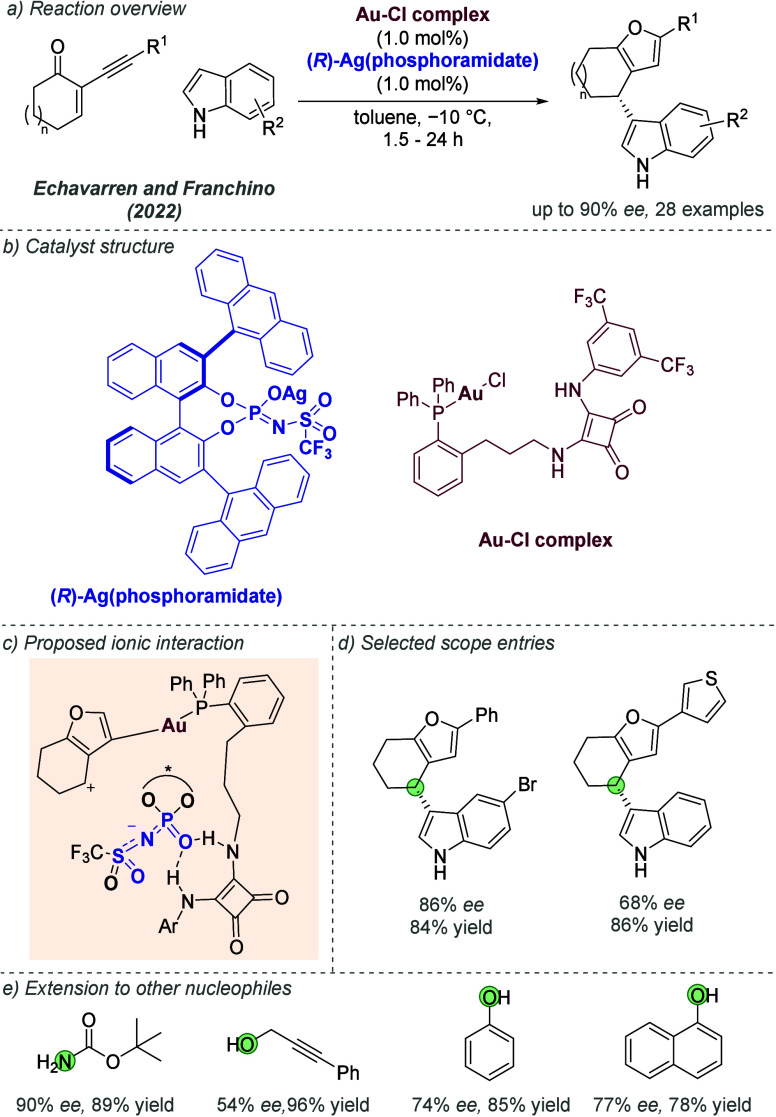
Enantioselective gold(I) tandem cycloisomerization and nucleophile addition of 2-alkynyl enynones using hydrogen bond-directed counteranion catalysis.

In 2022, a follow-up study was reported by Echavarren, Franchino and co-workers, in which their hydrogen-bonded counterion strategy was explored in greater detail for cycloisomerization/addition to enynones (Figure 58a).^136^ High throughput experimentation (HTE) on a 10 μmol scale was used to screen combinations of gold(I) chloride complexes featuring hydrogen bond donors, as developed in their previous study, together with various chiral silver salts (and a copper salt) of phosphoramidates. The HTE study showed that the reactions could be catalyzed by the presence of the silver/copper phosphoramidate salt alone, which is precedented, albeit with diminished enantioselectivity. Subsequent optimization identified the optimal silver phosphoramidate and gold complex (Figure 58b). Variously substituted indoles and enones could be used (selected examples in Figure 58d). The authors also investigated alternative nucleophiles, with a range of *N*-centered and *O*-centered nucleophiles proving competent but with varying *ee* values (Figure 58e).

**Figure 59:**
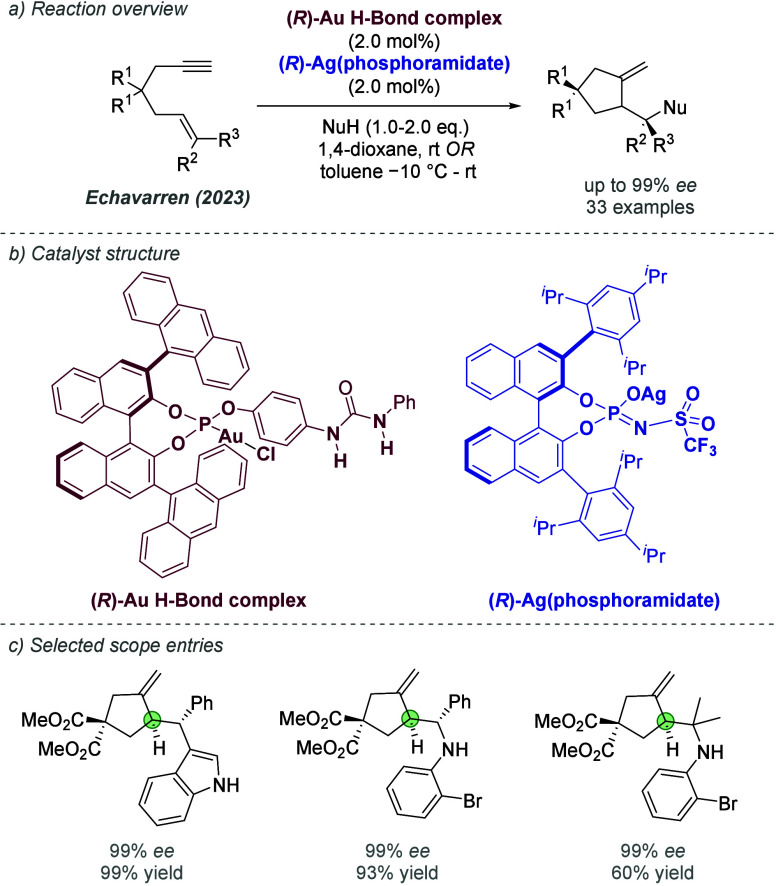
Enantioselective addition of N,O and C-based nucleophiles to 1,6-enynes via hydrogen bonded matched ion pair gold(I) catalysis.

In 2023, Echavarren and co-workers presented further studies using their ligands; in this report, phosphite ligands bearing ureas were explored rather than phosphines (Figure 59b).^137^ This allowed chirality, in the form of BINOL derivatives, to be incorporated into the ligand as well as the counteranion, potentially leading to matched/mismatched effects. The authors explored this in the context of gold(I)-catalyzed addition of nucleophiles to 1,6-enynes (Figure 59a). The optimal phosphine identified in the earlier work on cycloisomerization of 1,6-enynes (see Figure 57) showed poor enantioselectivity and reactivity in this case.^134^ The authors synthesized a library of BINOL-derived phosphites bearing a urea at various positions of the third (non-BINOL) aromatic substituent, of which the *para*-isomer was optimal (Figure 59b). This was combined with a TRIP-derived silver phosphoramidate. A variety of nucleophiles were competent in the reaction, including indoles, electron-rich aromatics, anilines and alcohols (Figure 59c). DFT-predicted noncovalent interaction plots revealed the expected chiral anion-urea interaction, as well as various weaker attractive noncovalent interactions, which all contributed to the high enantioselectivity of the process.

In 2023, Marinetti, Guinchard and co-workers further explored their tethered counterion strategy in a gold(I)-catalyzed tandem cycloisomerization-addition reaction of 2-alkynyl enynones and naphthols.^138^ The reaction can occur at either oxygen or carbon to give the *O*- and *C*-linked products–the ratios obtained varied drastically with conditions, time and specific catalyst used. They identified conditions which could effect either *O*- or *C*- addition, depending on the precise conditions, and attempted to rationalize the results using DFT investigations.

**Figure 60:**
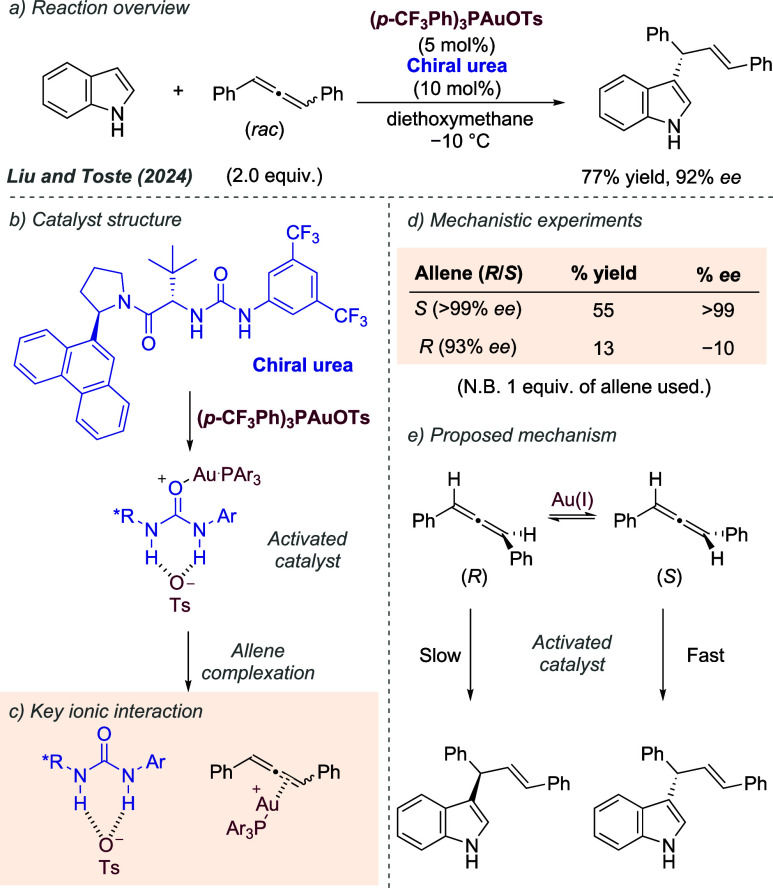
Enantioselective addition of indole to diphenylallene using gold(I) catalysis with anion binding by chiral urea.

In 2024, Toste, Liu and co-workers disclosed investigations of a strategy for asymmetric gold(I) catalysis, in which a chiral hydrogen bond donor catalyst is used to bind the achiral anion of a gold complex (Figure 60a).^139^ This was inspired by Jacobsen and co-workers’ used of anion binding in asymmetric organocatalysis and was reported shortly after Jacobsen’s successful transposition of the strategy to ion-paired Ru catalysis (see later, Figure 86). In this work, the authors reported a single highly enantioselective example of an indole nucleophile reacting with a racemic diphenylallene electrophile to afford the C_3_-substuted indole in good yield. Their mechanistic proposal involved the activation of the chiral urea by the gold(I) precatalyst to afford the anion-bound activated catalyst (Figure 60b). The gold(I) can dissociate from the carbonyl of the urea and complex the allene to form the key ion paired species: a bound tosylate anion within a chiral environment ion-paired with the cationic allene-gold complex (Figure 60c). Mechanistic experiments were carried out by subjecting enantioenriched allenes to the reaction conditions (Figure 60d). The *S*-configured allene reacted in 55% yield while maintaining the stereochemical integrity of the allene starting material, while the *R*-configured allene afforded much poorer yield with loss of stereochemical information. Based on these observations, a mechanism invoking a dynamic kinetic resolution of the two allene enantiomers was proposed (Figure 60e). The two allene enantiomers exist in equilibrium in the presence of a gold(I) catalyst; the *S*-configured allene is matched with the (chiral) activated catalyst, reacting quickly, while the *R*-configured allene is mismatched, so reacts slowly. DFT calculations were carried out which supported the hypothesis, and also explained why the NH indole is important for high *ee*, through hydrogen bonding with the catalyst at the transition state (*N*-Me indole gave much poorer *ee*).

#### 3.1.2 Palladium

Palladium is an example of a transition metal for which many catalytic cycles are known to involve cationic complexes. It is therefore amenable to design strategies which involve chiral counteranions. It should be noted that here we will cover examples where the authors have suggested that they believe or imply in the language used that the chiral component, most commonly a phosphate, is acting as a counterion to the charged metal. This means that we will not cover examples where the authors explicitly propose that it is bound as an ionic ligand. The caveat is that in many cases it is difficult to determine this definitively. For a full recent survey of chiral phosphoric acids with palladium, the reader is directed to an excellent review from Engle and co-workers.^140^ Important early studies from Alper and Hamel demonstrated that in the palladium catalyzed hydrocarboxylation of styrenes, a phosphoric acid derived simply from enantiopure BINOL (a common resolving agent) could control enantioselectivity very effectively.^141^ The reaction mixture was complex, consisting of a palladium salt, a copper salt, concentrated HCl, and water, and very high enantioselectivities could be obtained. There was no speculation on the role of the acid, but this early work clearly indicated the potential of using chiral Bro̷nsted acids in combination with transition metal catalysis. The reactions in this section are broadly split into two categories, which are covered sequentially. First, we discuss those which feature a cationic π-allyl palladium intermediate. These species can be paired with a chiral anion and form a stereocenter with enantiocontrol when attacked by a nucleophile. In the latter part of this section, we cover examples in which asymmetry is proposed to arise during stereocontrolled insertion of an olefin into a cationic, ion-paired R-Pd(II) species.

**Figure 61:**
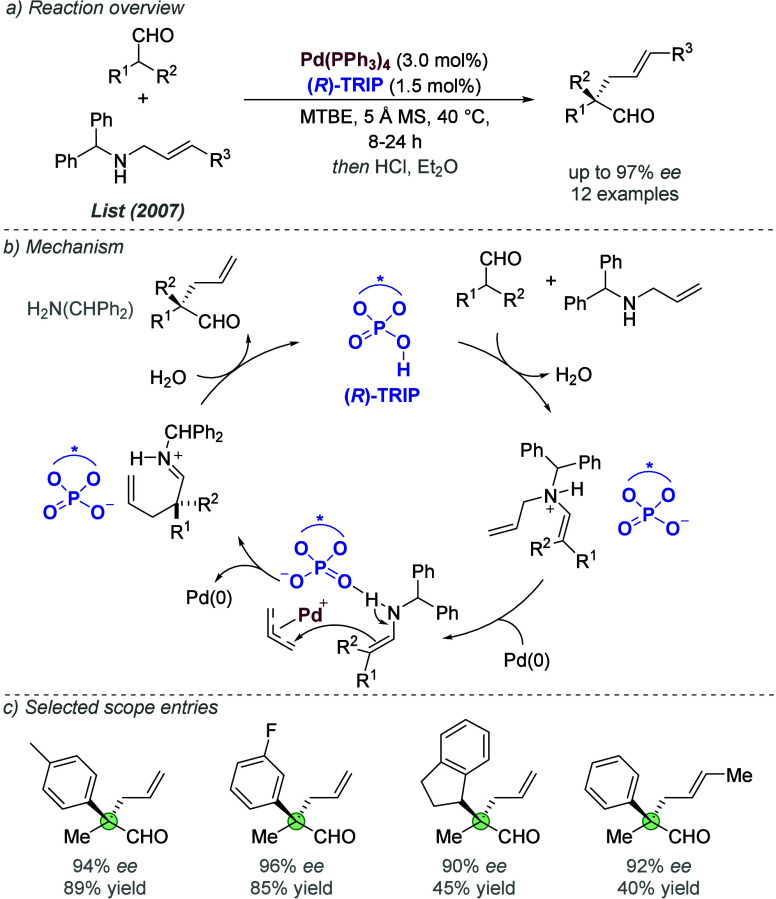
Enantioselective α-allylation of aldehydes proceeding via a cationic π-allyl palladium complex that is paired with a chiral anion.

Important early work incorporating ionic interactions into fundamental palladium catalyzed reactions was disclosed in 2007 by Mukherjee and List, in the context of π-allyl palladium chemistry.^142^ This built on earlier work from the List group using phosphates as chiral anions in organocatalysis, which the authors termed ‘asymmetric counteranion-directed catalysis’.^143^ The authors disclosed an α-allylation of α-branched aldehydes using catalytic Pd(PPh_3_)_4_ and (*R*)-TRIP, achieving *ee* values of up to 97% (Figure 61a). Mechanistically, they proposed that the chiral Bro̷nsted acid begins the cycle by catalyzing the condensation between an allyl amine and the aldehyde, forming a protonated enamine which is now ion-paired with the chiral phosphate (Figure 61b). Next, Pd(0) enters the cycle and, with an ammonium leaving group, forms the π-allyl Pd (II) complex which is likely to be cationic and thus ion-paired with the chiral phosphate. Subsequent addition of the enamine to the π-allyl ligand takes place within the chiral environment established by the chiral phosphate counterion, potentially involving hydrogen bonding with the enamine NH. Reformation of aldehyde and ejection of the amine byproduct closes the cycle. After optimization, including variation of the solvent, temperature and phosphoric acid cocatalyst, excellent yield and enantioselectivity could be obtained, which enabled successful exploration of aldehydes with differing electronic properties, as well as variation of the allyl group (Figure 61c). It was also showed that the methodology could be applied to the synthesis of the natural product (+)-cuparene. The authors acknowledge that the phosphate might be acting as an anionic ligand for palladium. But whatever the precise interaction, this elegant design strategy was clearly influenced by the widely acknowledged presence of cationic intermediates in many such reactions, and was an influential application of the chiral counteranion strategy to asymmetric palladium chemistry. In 2014, Jindal and Sunoj reported a computational study of this reaction, in which they proposed that the phosphate functions as an counteranion for Pd(II) during the enantiodetermining step, rather than an anionic ligand.^144^ They also identified weaker attractive noncovalent interactions between the chiral phosphate and various components of the Pd(II) complex, in addition to the anticipated hydrogen bond with the enamine.

**Figure 62:**
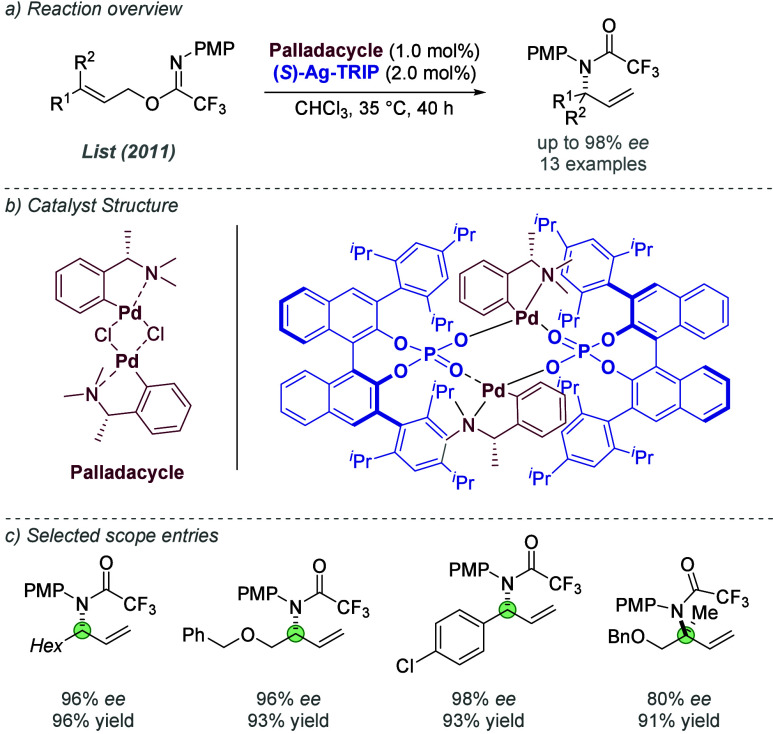
Enantioselective Overmann rearrangement catalyzed by a complex formed from Ag-(*S*)-TRIP and a chloropalladacycle, giving rise to highly enantioenriched allylic amines.

In 2011, List and co-workers combined chiral silver phosphates with chloropalladacycle catalysts in a further development of their chiral counterion strategy.^145^ They used these to effect an asymmetric Overman rearrangement of allylic imidates to afford the corresponding allylic amide products in good yields and excellent enantioselectivities (Figure 62a). Chiral palladacycles bearing complex chiral ligands had been used in the past to render such reactions enantioselective. The authors speculated as to whether the bridging halides could be replaced by chiral phosphates. After evaluating a selection of both chiral and achiral palladacycles, the chiral, enantiopure palladacycle indicated was paired with the silver salt of (*S*)-TRIP, to give the allylic amide product in excellent yield and 98% *ee* (Figure 62b). Control experiments showed that in the absence of the silver phosphate, racemic product was obtained, and that the silver phosphate alone did not catalyze the rearrangement. A variety of allylic imidates were evaluated with differing steric and electronic profiles, and variously substituted aryl rings could also be incorporated on the alkene (Figure 62c). Furthermore, sterically encumbered branched substituents could undergo the rearrangement to give quaternary products with good *ee* (80% *ee*), albeit with higher catalyst loadings. The authors next sought a solid-state structure of their presumed *in situ* formed catalyst. The palladacycle was therefore treated with two equivalents of (*S*)-TRIP, to give a compound that was assigned as having bridging phosphates between the two palladium centers (Figure 62b, right). This was found to be catalytically active, affording identical results to the *in situ* procedure, supporting the notion that it may be the active complex. An X-ray structure was obtained after addition of methylimidazole, showing that the phosphate acts as an anionic ligand to the Pd in the solid state, rather than a dissociated counterion. The authors used this structure to advance a stereochemical model to account for the observed stereoselectivity, with the phosphate now acting as an anionic ligand.

**Figure 63:**
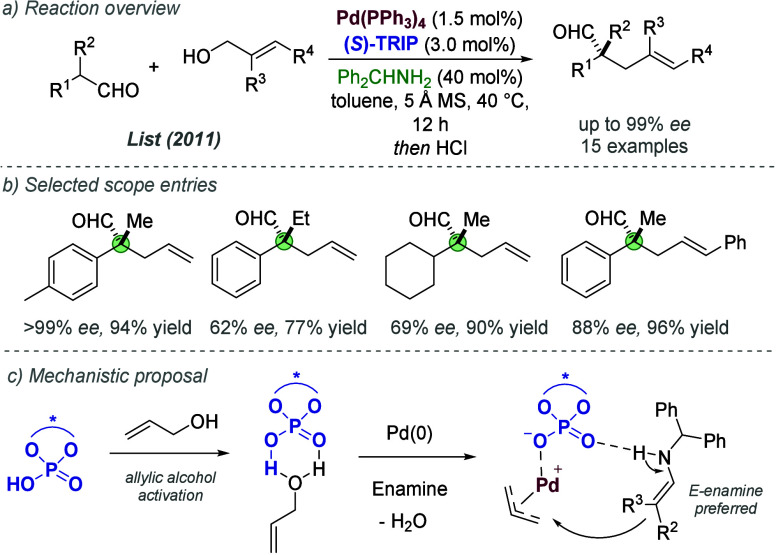
Asymmetric α-allylation of aldehydes with allylic alcohols, proposed to proceed via an ion-paired π-allyl palladium intermediate.

In 2011, Jiang and List reported a further development of their asymmetric α-allylation of aldehydes, in which allylic alcohols could be used to generate the key π-allyl palladium intermediate (Figure 63a).^146^ This built on an earlier report by the same authors from the same year, using achiral Bronsted acids to achieve this objective in a racemic manner.^147^ Their previous asymmetric protocol for aldehyde α-allylation necessitated the presynthesis of benzhydryl allyl amines, which were then reacted with the α-branched aldehyde (Figure 61).^142^ In the present work they use a simple allylic alcohol as the allyl partner, now in combination with a triple catalytic system of 3 mol % (*S*)-TRIP, 1.5 mol % Pd(PPh_3_)_4_ and 40 mol % benzhydrylamine. This gave excellent enantioselectivities for the α-allylated aldehyde products. The scope of the reaction with respect to the allylic alcohols was explored: branched aldehydes with various aromatic substitution were well tolerated (Figure 63b). Aliphatic branched aldehydes could also be allylated, albeit with lower *ee* – a cyclohexyl substituent gave 69% *ee* with increased catalyst loadings and use of a chiral amine cocatalyst. The authors proposed a mechanism that was largely based on earlier work; the key difference is that the chiral phosphoric acid is proposed to activate the allylic alcohol toward oxidative addition from the Pd(0) catalyst, through two hydrogen bonding interactions (Figure 63c, left). Next, an ion-pair is formed between the cationic Pd-π-allyl complex and chiral phosphate anion, while the chiral anion can also hydrogen bond to the *E*-configured enamine (Figure 63c, right). This network of interactions controls the enantiodetermining formation of the C–C bond.

**Figure 64:**
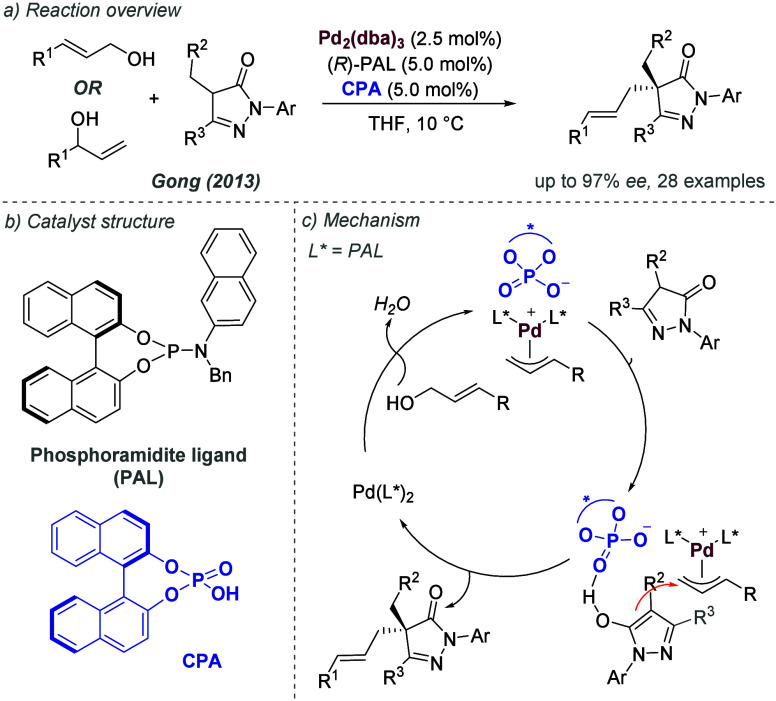
Asymmetric allylic alkylation of pyrazol-5-ones with allylic alcohols.

In 2013, the Gong group reported a related asymmetric allylic alkylation reaction between pyrazol-5-ones and allylic alcohols, catalyzed by a Pd complex bearing a chiral phosphoramidate ligand in combination with a chiral phosphoric acid cocatalyst (Figure 64).^148^ Optimization studies showed that the chirality of the phosphoramidite ligand was playing the major role in determining product *ee*, but there was a clear matched/mismatched effect with the different enantiomers of BINOL phosphoric acid (e.g., 81% vs 90% *ee* for one substrate, not shown) with the (*R*)-configured CPA required to obtain the highest selectivities (Figure 64b). The mechanistic proposal resembles the earlier transformation from List and co-workers: the phosphoric acid assists in generating the key cationic π-allyl palladium complex from the allylic alcohol (Figure 64c). A hydrogen bond can then form between the enolized pyrazol-5-one and the CPA, which is itself ion-paired to the π-allyl palladium intermediate. This network of interactions enables stereocontrol in the subsequent attack of the nucleophile.

**Figure 65:**
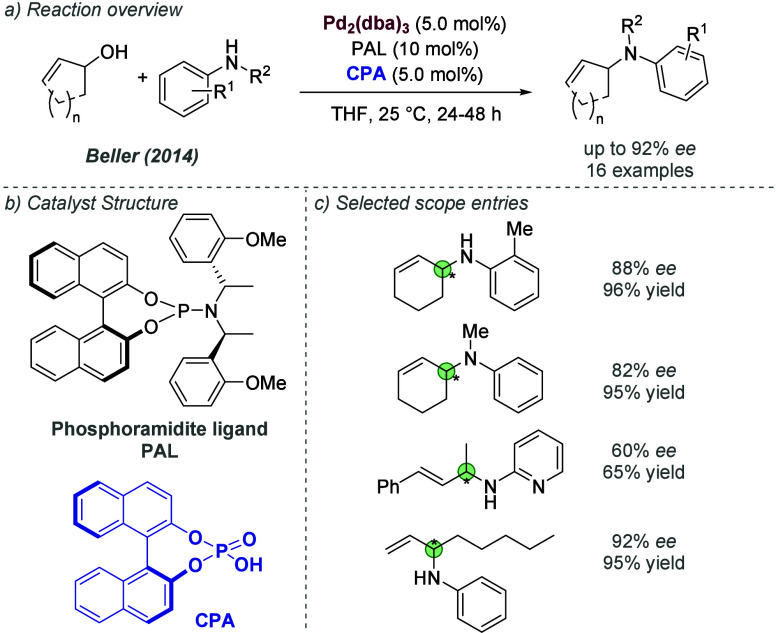
Asymmetric allylic amination of racemic alcohols with aniline derivatives utilizing posphoramidite ligands and chiral phosphoric acids.

Also related to List’s 2011 report using allylic alcohols, in 2014 Beller and co-workers reported an enantioselective amination of racemic allylic alcohols, using a phosphoramidite ligand on palladium together with a chiral phosphoric acid (Figure 65).^149^ Use of the chiral phosphoramidite ligand in the absence of a chiral phosphoric acid cocatalyst resulted in a poorer 40% yield and 44% *ee* (not shown). The addition of 5 mol % of the depicted BINOL-derived chiral phosphoric acid improved both metrics, to 95% yield and 92% *ee*. Meanwhile, the racemic form of this chiral phosphoric acid gave only 20% yield and 60% *ee*, highlighting the important role of the acid. The authors explored the scope of this transformation with respect to the aniline derivatives (Figure 65c). Although a detailed mechanistic proposal is not advanced, the authors suggest that the π-allyl palladium complex generated is stabilized by the anionic phosphate, which presumably plays an important role in the asymmetric induction.

**Figure 66:**
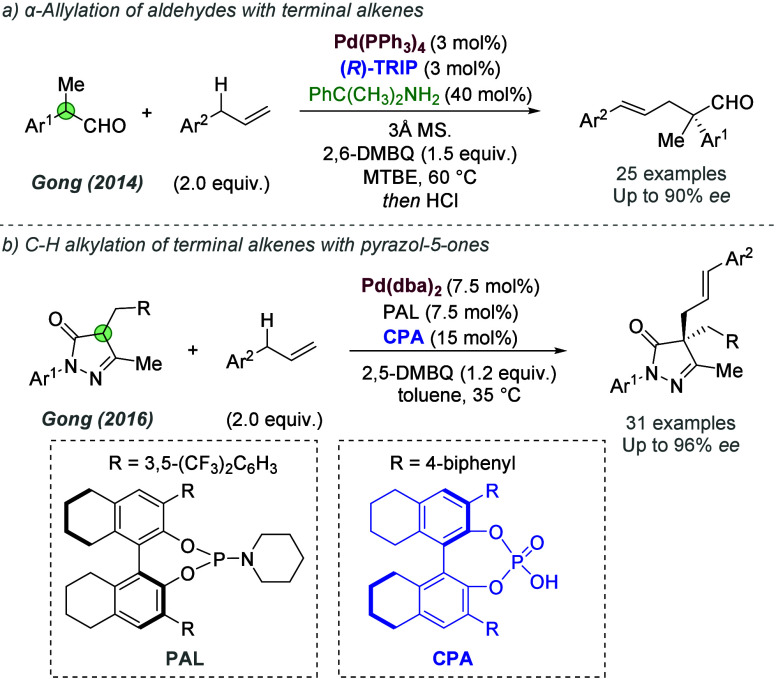
Enantioselective allylic alkylation controlled by chiral phosphate anions, proceeding via C–H activation.

Also in 2014, Gong and co-workers developed a variant of List’s 2011 report of aldehyde α-allylation in which the cationic π-allyl palladium complex is generated by C–H activation, in the presence of 2,6-dimethylbenzoquinone as an oxidant (Figure 66a).^150^ They were able to combine the required reaction conditions for the allylic C–H activation processes with those conducive to high asymmetric induction, when they intercept what is presumably an analogous intermediate. In 2016, the same authors disclosed a related allylic C–H activation of allylarenes, with the resulting π-allylpalladium complex being trapped by a pyrazol-5-one as in their 2013 report (*vide supra*) (Figure 66b).^151^ In this reaction, most of the selectivity appears to derive from the chiral phosphoramidite ligands employed–use of an achiral phosphoric acid still gave high *ee* (84%) but this could be improved to 94% with a bulky chiral phosphoric acid. The same report also described an analogous C–H activation with 1,4-pentadienes (not shown). The optimized conditions for these substrates deployed an achiral anion, though this was still proposed to engage in a hydrogen bonding interaction with the substrate.

**Figure 67:**
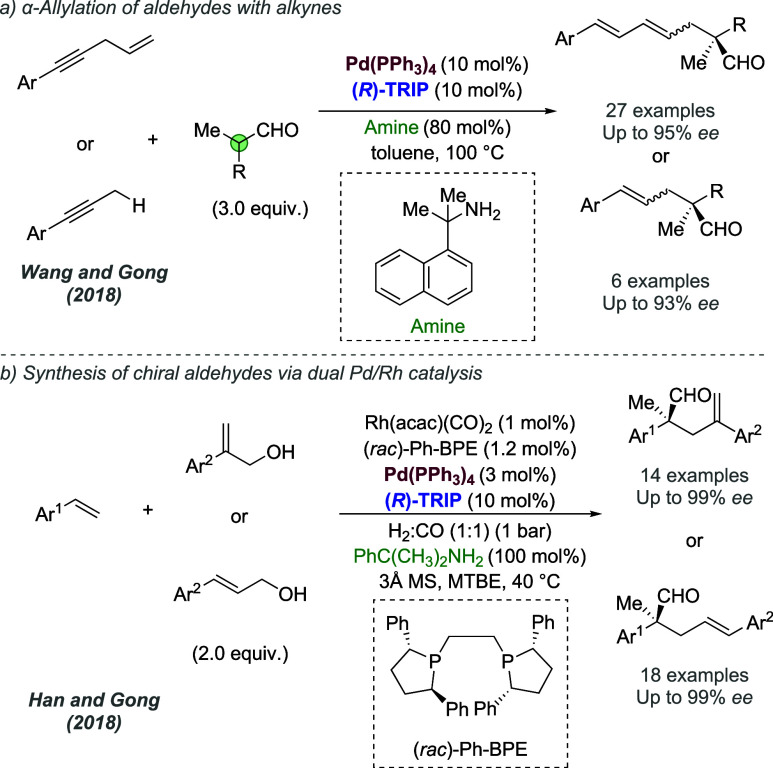
Further developments in enantioselective alkylation of aldehydes, either discrete or formed *in situ*, controlled by chiral phosphate anions.

In 2018, Wang, Gong, and co-workers reported an α-allylation of aldehydes using alkynes as electrophiles, which intercepts an analogous intermediate to List’s 2011 report. The difference here is that the π-allyl palladium complex is generated from addition of a palladium hydride complex to the alkyne, via an allene (Figure 67a).^152^ The palladium hydride that enacts the isomerization is proposed to arise from the oxidative addition of a Pd(0) complex to the chiral phosphoric acid. In 2018, Han, Gong, and co-workers disclosed another clever adaptation of List’s original report, by forming the α-branched aldehyde *in situ* from an alkene via a branch-selective Rh-catalyzed hydroformylation (Figure 67b).^153^ This required a Rh catalytic cycle to work alongside a Pd cycle, and the chiral phosphoric acid was proposed to exert stereocontrol in the Pd cycle according to the same chiral anion strategy reported by List, forming similar products. The multitude of catalytic strategies used to generate similar key intermediates, which are also compatible with the chiral anion-controlled enantioinduction, is a testament to its versatility.

**Figure 68:**
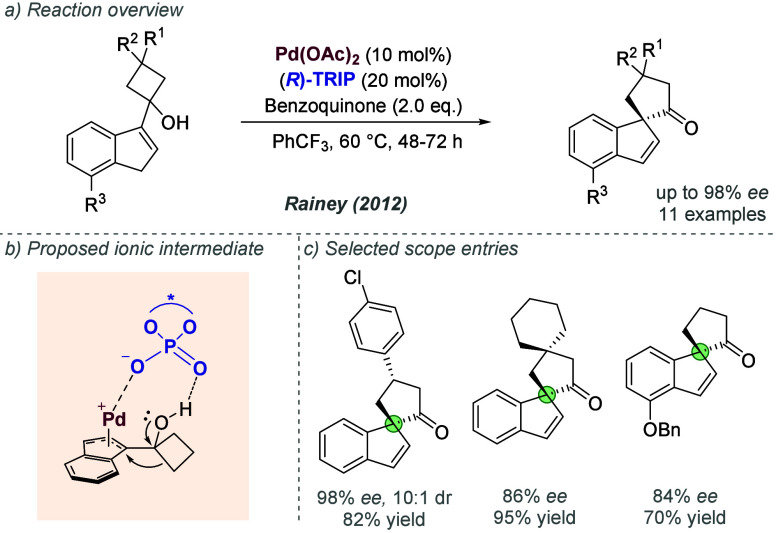
Allylic C–H activation/semipinacol rearrangement catalyzed by (*R*)-TRIP and Pd(OAc)_2_, to access chiral spirocyclic compounds.

In 2012, Chai and Rainey reported allylic C–H activation to give chiral spirocyclic products in high *ee*, through a migratory ring expansion (Figure 68a).^154^ This was achieved through combining a Pd(II) catalyst with a chiral phosphoric acid. Various chiral phosphoric acids were investigated with (*R*)-TRIP giving the best enantioselectivity (up to 77% *ee* on the optimization substrate) with benzoquinone included as a terminal oxidant. Scope evaluation showed that substrates with substituents at the 4-position of the cyclobutyl ring gave higher levels of enantioselectivity (up to 98% *ee*) and good dr (up to 10:1) (Figure 68c). Various alkyl and aryl groups could also be incorporated at different positions. Experiments support a mechanism in which palladium initially binds to the alkene, with this species undergoing C–H activation to form a π-allyl palladium complex. A chiral phosphate is then proposed to replace the acetate on palladium, to form an ion pair with the cationic π-allyl palladium intermediate, although the authors also acknowledge the possibility that the phosphate could function as an anionic ligand (Figure 68b). Hydrogen bonding between the phosphoryl oxygen and the alcohol of the substrate is also likely, providing a high degree of organization for the ensuing rearrangement. Subsequently, Jindal and Sunoj published a computational study of the mechanism of this reaction. They proposed that, rather than direct C–H activation, a Wacker-type mechanism is followed, in which Pd(II) activates the alkene toward a semipinacol type rearrangement.^155^ Interestingly, the phosphates were proposed to remain ligated to palladium as anionic ligands. Although the bulky chiral phosphates used in the experimental study were not investigated here, they were explored in a follow up study from the same authors, which attempted to use DFT calculations to rationalize the stereoinduction.^156^ Similarly, two chiral phosphates were proposed to be ligated throughout the process, rather than counterions. Nevertheless, the ion-pairing design hypothesis which guided the experiments was still enabling and this highlights the challenge in this type of system in distinguishing between counterion and anionic ligand.

**Figure 69:**
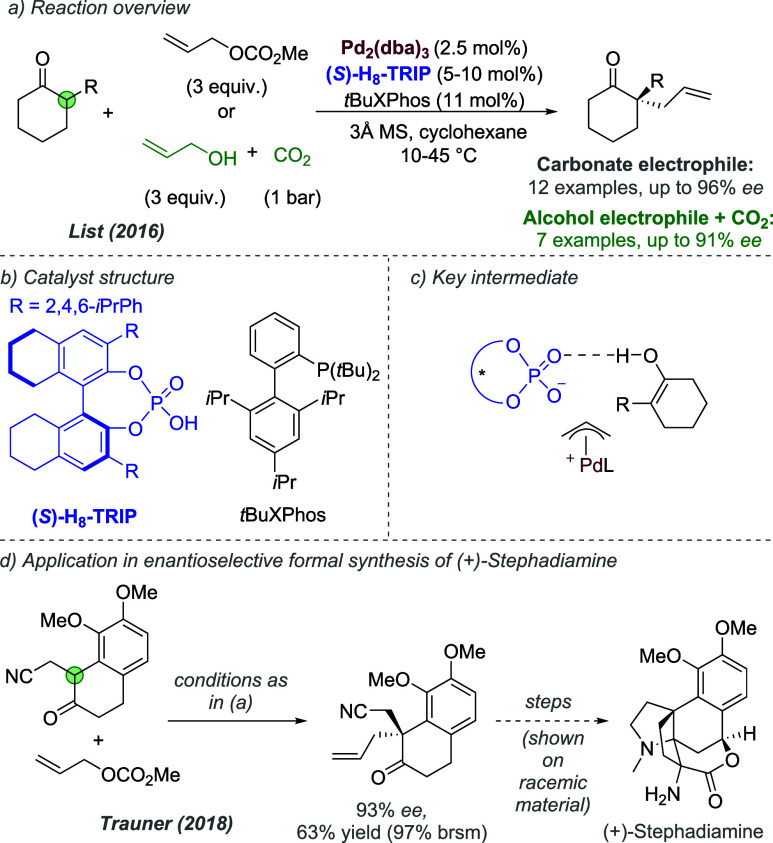
Enantioselective α-allylation of ketones and its application in a formal synthesis of (+)-stephadiamine.

Allyl carbonates are widely used as electrophiles in the Tsuji-Trost reaction. However, they typically require synthesis from the corresponding allylic alcohol. In 2016, List and co-workers bypassed this process by converting the allylic alcohol to a corresponding carbonate *in situ*, with CO_2_ formally acting as a catalyst, in combination with the chiral anion strategy previously developed (Figure 69a).^157^ The authors initially developed the reaction with carbonate electrophiles, providing proof of concept that enantioselective allylation of ketones with allylic electrophiles was indeed possible (Figure 69a, top electrophile). They then showed that an *in situ* formation of a π-allyl precursor from the allylic alcohol under an atmosphere of CO_2_ was also possible, while maintaining high levels of enantioselectivity (Figure 69a, bottom electrophile). Optimal results were obtained with a partially unsaturated variant of TRIP, (*S*)-H_8_-TRIP, in combination with *t*BuXPhos (Figure 69b). The alcohol is activated by CO_2_ to form a reactive carbonic acid ester. The nucleophile is activated by the chiral phosphoric acid in its more stable (substituted) enol form, which, when ion paired with the cationic palladium π-allyl intermediate, provides a chiral environment for an enantiodetermining attack of the electrophile (Figure 69c). In 2018, Trauner and co-workers applied this reaction in an enantioselective formal synthesis of the natural product (+)-stephadiamine (Figure 69d).^158^ An unsymmetrical ketone intermediate was allylated with a carbonate electrophile in 93% *ee*. The authors had previously completed a racemic total synthesis of stephadiamine from a racemate of the allylated intermediate; the application of List and co-workers’ reaction therefore constitutes a formal enantioselective synthesis of this natural product.

**Figure 70:**
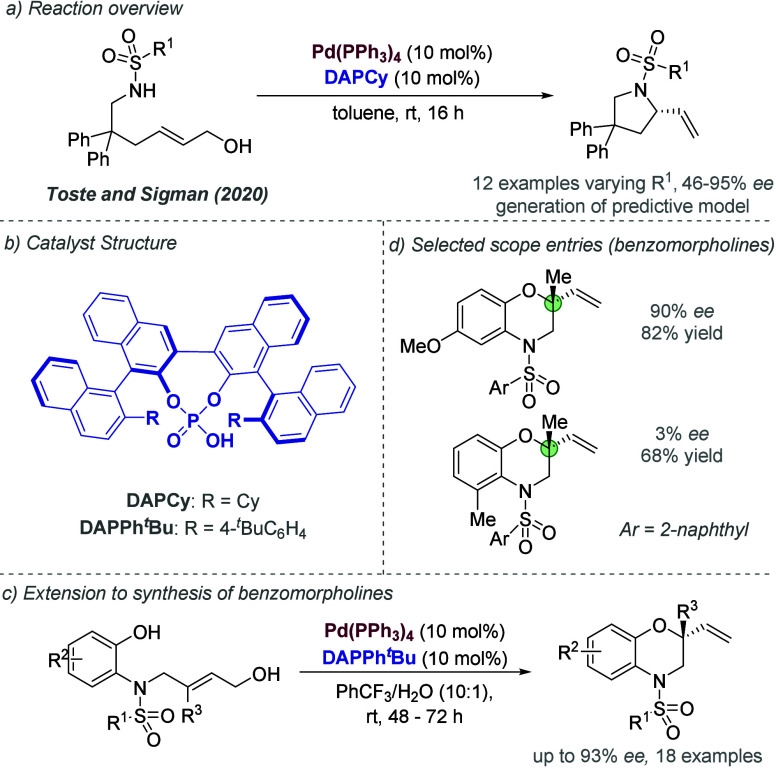
Enantioselective intramolecular allylic amination of allylic alcohols, using a doubly axially chiral phosphate anion.

In 2020, Toste and Sigman reported an intramolecular allylic amination, forming chiral pyrrolidines from allylic alcohols (Figure 70a).^159^ This is related to Beller and co-workers’ 2014 report described above (see Figure 65), but here no chiral ligand is used for palladium: the only chiral element is the phosphoric acid. Both palladium and a CPA were required for reactivity, with 84% *ee* obtained for the model substrate (Figure 70a, R = 1-naphthyl). Trichloroacetimidate substrates were also investigated, though these only required a CPA (with no palladium cocatalyst) to obtain high enantioselectivity (not shown). Under the Pd-free conditions, the analogous trichloroacetimidate formed the opposite enantiomer in 78% *ee*. If palladium was included with the trichloroacetimidate substrates, almost no *ee* was obtained, due to a rapid background reaction. In the palladium-catalyzed process (allylic alcohol substrates), a bespoke doubly axially chiral phosphoric acid (DAP), featuring bulky cyclohexyl groups, was required to obtain the highest levels of enantioselectivity (Figure 70b). Statistical modeling through catalyst and substrate parametrization was used to probe the origins of the differing enantioselectivities of the two catalytic mechanisms. After a series of detailed experiments, including careful deuterium labeling studies, the authors concluded that the enantiodetermining step in the palladium-catalyzed variant involves addition of the nucleophile into the π-allyl palladium complex, with the DAP anion ion-paired with the cationic palladium. Further modeling suggested that a noncovalent interaction between the sulfonamide oxygen and the DAP anion represents the most important interaction for determining the major and minor diastereomeric transition states during reaction of the π-allyl complex. The authors extended the reaction to synthesize chiral 2,2-disubstituted benzomorpholines using a different DAP catalyst featuring bulky *p*-*^t^*Bu phenyl substituents, and a broad scope was demonstrated (Figure 70c, d). In 2022, Tribedi and Sunoj reported a detailed computational evaluation of the rection pathway.^160^ This concluded that in the enantiodetermining step–the nucleophilic addition to the cationic π-allyl palladium complex–the DAP is indeed functioning as a chiral counterion. Recently, Tsai and co-workers reported a variant of this reaction in which the substrate contains a tethered oxygen nucleophile, forming 1,3-dihydroisobenzofurans, in a kinetic resolution of secondary alcohols.^161^

In 2024, White and co-workers reported a palladium-catalyzed coupling of alcohols and alkenes, which proceeded via a cationic π-allyl palladium complex.^162^ The authors found that a substoichiometric phosphoric acid additive was important for reactivity, and they proposed that the corresponding phosphate functions as an anion to the π-allyl palladium intermediate. The phosphate is proposed to hydrogen bond to the incoming alcohol and dramatically increase reactivity for the nucleophilic addition. A large reaction scope was demonstrated, and extensive experiments were carried out to quantify the rate accelerations and provide support for the proposed interaction. The potential selectivity aspect of this process is between the branched and linear products; here, the linear product was heavily favored under most conditions evaluated. The main benefit of this work is rate acceleration and improved reactivity–it therefore falls outside the scope of the present survey. The optimization process demonstrated linear:branched selectivity increases using the ligand/additive combinations that were proposed to result in the optimal positioning of the counterion. For example, selectivity improved from 19:1 to 43:1 when switching from the TFA anion to DEHPA anion (the latter was proposed to have the optimal balance of noncoordinating and hydrogen bonding ability). Similarly, it improved from 20:1 to 50:1 when switching from the *trans*-SOX ligand to the *cis*-SOX (the latter was proposed to better position the anion to guide the nucleophile).

**Figure 71:**
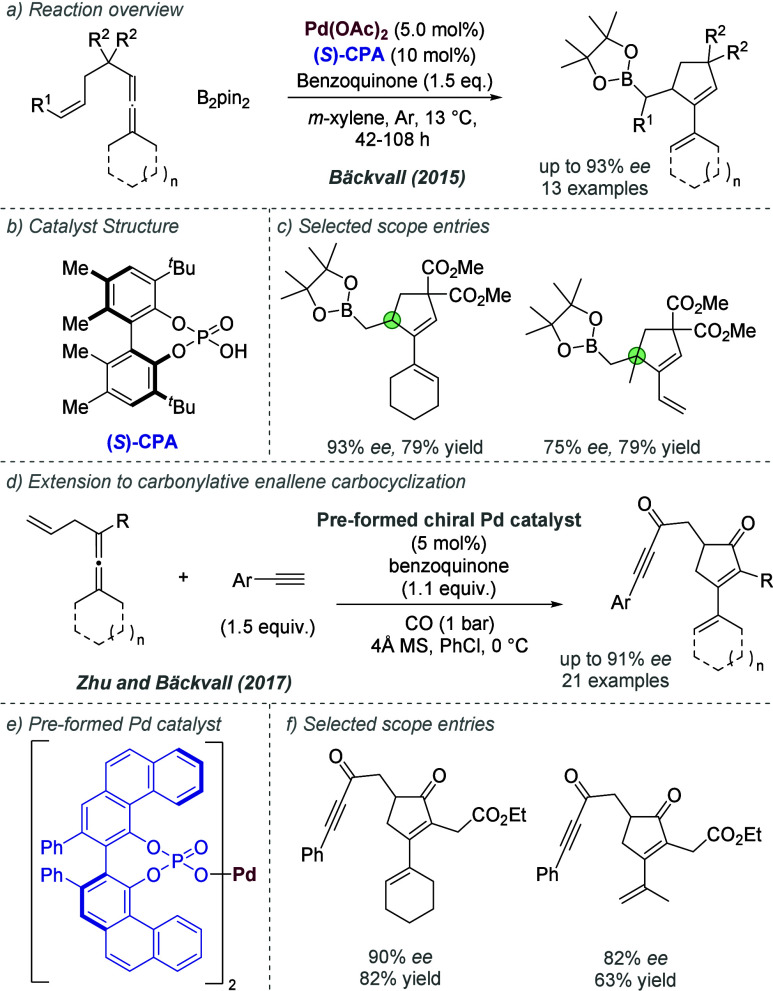
Enantioselective oxidative carbocyclization-borylation reaction catalyzed by Pd(II) in combination with a chiral phosphoric acid, and extension to carbonylative enallene carbocyclization.

The next few examples belong to the second category, in which enantioselectivity is hypothesized to occur via a counteranion-directed insertion of an alkene into a cationic R-Pd(II) species. In 2015, Bäckvall and co-workers explored the enantioselective oxidative carbocyclization-borylation of enallene substrates (Figure 71a).^163^ A combination of Pd(OAc)_2_, chiral phosphoric acid, B_2_pin_2_ and benzoquinone gave rise to the desired borylated carbocycles in high yield and enantiomeric excess (Figure 71a and b). Substitution at the terminus of the allene was investigated, with 5-, 6-, 7- and 8-membered rings being tolerated in good enantioselectivities (88–93% *ee*). For the alkene component, an unsubstituted terminal alkene worked best–disubstitution was found to drastically retard the reaction. No hypothesis regarding the mechanism was explicitly stated, although the authors envisaged a chiral counteranion strategy, replacing acetate with a phosphate, that had been demonstrated by List and others in the preceding years. Zhu, Bäckvall, and co-workers followed this up two years later with a highly enantioselective carbonylative carbocyclization of enallenes, which terminates in the coupling with an alkyne (Figure 71d).^164^ In this case, optimal results were achieved using a preformed palladium catalyst derived from Pd(OAc)_2_ and (*R*)-VAPOL-PA (Figure 71e), while using the same benzoquinone oxidant as the earlier report. The reaction scope included considerable variation of the aryl substituent on the alkyne. Regarding the lower terminus of the allene, various ring sizes as well as an acyclic group were tolerated, although these incurred a reduction in *ee* compared with the 6-membered ring (e.g., Figure 71f left vs right). The authors again state that they envisage a chiral anion strategy, although the precise nature of the interaction between the phosphate and palladium was not elucidated.

**Figure 72:**
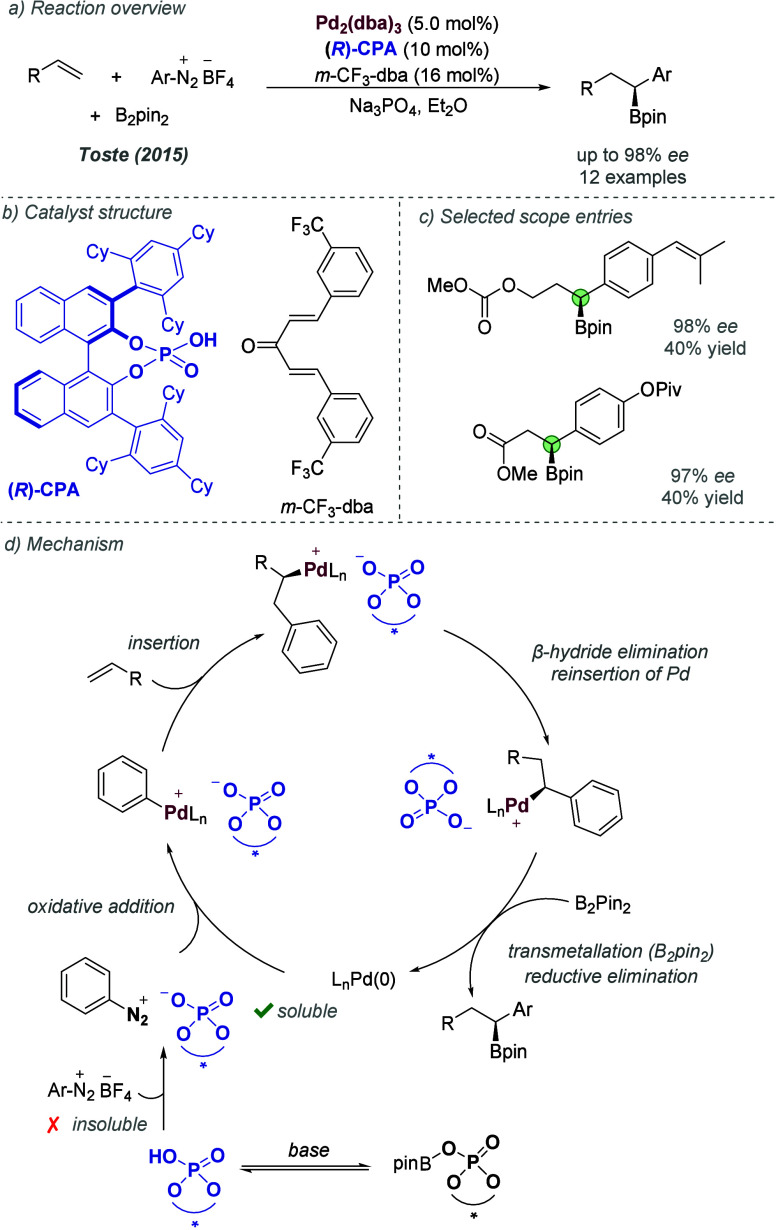
Enantioselective three-component coupling of alkenes, aryldiazonium salts and bis(pinacolato)diboron to access chiral benzylic boronic esters, leveraging ion-pairing between a chiral phosphate and cationic Pd intermediates.

In 2015, Toste and co-workers reported a palladium-catalyzed three-component coupling of aryl diazonium salts, B_2_pin_2_ and α-olefins, which gave rise to chiral benzylic borylated esters (Figure 72a).^165^ Initially a racemic protocol was developed, but the authors’ main goal was to develop an enantioselective version; this was considered challenging, because previous related studies had shown that the addition of ligands shuts down the desired three component reaction.^166^ They envisaged that asymmetry might be induced using a chiral anion strategy, since the key palladium(II) intermediates in the cycle are likely cationic. The chiral phosphoric acid depicted, featuring 2,4,6-(Cy)_3_C_6_H_2_ at the 3,3′ positions, was found to give excellent enantioselectivity in the three-component coupling of allyl methyl carbonate, phenyl diazonium tetrafluoroborate and B_2_pin_2_ (Figure 72b). Tuning of the inorganic base and addition of a substituted dibenzylideneacetone (dba) additive allowed a yield of 40% on the optimization substrate (not shown), while retaining high levels of enantioinduction (90% *ee*). The aryldiazonium salt could be modified to feature various electron-donating groups, and while enantioselectivies were high, yields were typically moderate. As well as protected allylic alcohols, α,β-unsaturated esters were also viable substrates (Figure 72c). The authors propose a dual catalytic cycle: the diazonium salt is insoluble in the nonpolar solvent used (Figure 72d, lower left), but the lipophilic chiral phosphate can undergo anion exchange to form a soluble salt which can then undergo oxidative addition to Pd(0). This forms a putative cationic aryl-Pd(II) complex that is ion-paired with the chiral phosphate. The olefin can undergo insertion into the palladium, followed by β-hydride elimination and a reinsertion to give rise to another cationic Pd complex, still paired to the chiral phosphate. Subsequent transmetalation of B_2_pin_2_, followed by reductive amination, gives the final enantioenriched product.

In 2016 Gong and co-workers described a three-component coupling of dienes, aryldiazonium salts and aldehydes. The reaction was catalyzed by palladium, and the authors applied the chiral anion phase transfer approach recently reported by Toste to induce asymmetry.^167^ In this report, the mechanistic proposal depicts the phosphate as being ligated to the palladium.

**Figure 73:**
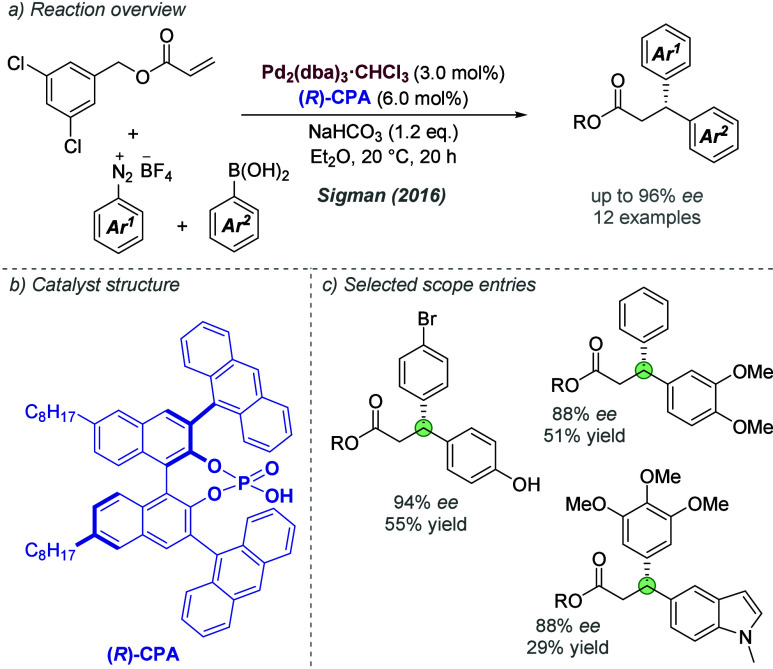
Enantioselective three-component coupling of benzyl acrylates, aryldiazonium salts and boronic acids, in which ion-pairing of a chiral phosphate with cationic Pd intermediates is invoked.

Also in 2016, Sigman and co-workers, in collaboration with Toste, built on the 2015 report from Toste and co-workers to develop a palladium-catalyzed three-component 1,1-diarylation of acrylates using a chiral anion phase transfer catalysis strategy to induce enantioselectivity (Figure 73a).^168^ In this work, aryl diazonium salts and aryl boronic acids were used as the two independent aryl sources, and were reacted with benzyl acrylate. Transmetalation of the cationic alkyl-Pd(II) intermediate, which may be stabilized as a π-benzyl species, must now occur with an aryl boronic acid rather than the B_2_pin_2_ used in the Toste study. This intermediate is envisaged to remain ion-paired with the chiral phosphate throughout to permit asymmetric induction, in analogy with the previous mechanistic proposal (see earlier, Figure 72). A series of chiral phosphoric acids possessing different electronic and steric profiles were synthesized and evaluated, revealing that steric bulk in the 3,3′ positions resulted in increased enantioselectivity, along with *n*-octane substitution at 6,6’ positions, giving rise to the optimal catalyst depicted (Figure 73b). Sigman and co-workers correlated molecular descriptors with the enantioselectivity of the reaction using their multidimensional parametrization technique, developing a predictive model for catalyst structure.^169^ For the benzyl acrylate component, the authors examined a diverse array of electronic and steric properties to assess how this would impact selectivity. Multidimensional parametrization identified trends which suggested attractive interactions, including π-stacking between the benzyl acrylate and the anthracenyl group on the phosphate, were contributing significantly to the high levels of enantioselectivity observed in the reaction. The scope with respect to the aryl boronic acid and diazonium salt was investigated in combination with 3,5-dichlorobenzyl acrylate (Figure 73c). A wide range of aryldiazonium salts could be used with varying electronic properties, while the aryl boronic acid component required an electron-rich arene.

In 2017, the same authors reported a detailed mechanistic investigation of the enantioselective 1,1-diarylation of benzyl acrylates.^170^ This study revealed the important role of the phosphate anion in the mechanism, which was proposed to play multiple roles. The phosphate was shown to be capable of acting as a base, which can promote undesired β-hydride elimination and lead to Heck products. Increasing the steric bulk on the phosphoric acid can hinder this pathway, leading to higher yields of the desired product. Regarding stereoinduction, in depth multidimensional analysis and transition state computations revealed that the phosphoric acid 3,3′ substituents (anthracenyl groups being optimal) engage in attractive π-stacking interactions with the substrate. Multivariate correlations revealed that the substrate hydrogens can engage in attractive electrostatic interactions with the phosphate group during the enantiodetermining step; these were further probed by DFT calculations. In this work, the chiral phosphate is depicted as bound to the Pd(II) metal center as a ligand, unlike the counterion hypothesis that guided the original work.

**Figure 74:**
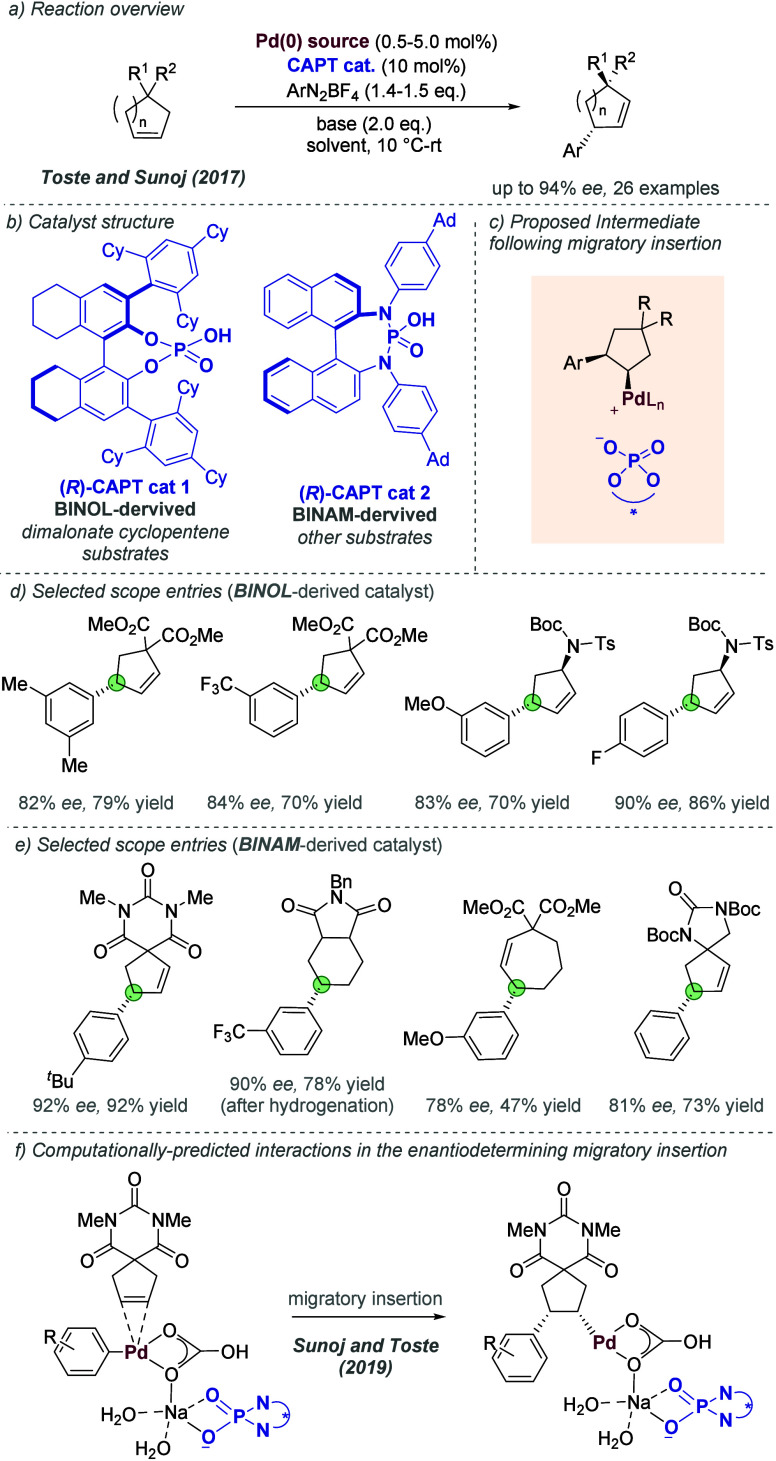
Asymmetric Heck-Matsuda reaction using chiral phosphate anions through chiral anion phase-transfer of aryl diazonium salts.

Also in 2017, Toste, Sunoj and co-workers, in collaboration with Sigman, reported an enantioselective Heck-Matsuda reaction using their chiral anion phase-transfer approach with aryl diazonium salts (Figure 74a).^171^ As before, it was envisaged that phase transfer of the diazonium salt into solution using a lipophilic chiral phosphate would, after oxidative addition to Pd(0), result in a cationic aryl-Pd complex ion-paired with the chiral phosphate. This ion pair could then enable a stereocontrolled migratory insertion, forming the complex depicted in Figure 74c. Interestingly, it was also shown that the chiral anion was able to suppress undesired reaction pathways such as alkene isomerization, which was consistent with related findings from the mechanistic studies on the 1,1-diarylation reaction, summarized above.^170^ Evaluation of chiral phosphoric acids revealed a partially unsaturated backbone to be optimal (Figure 74b, left), allowing high *ee* and yield for the Heck-Matsuda product. The scope of the aryl diazonium source was investigated; the authors also found that the quaternary center could be replaced with a single *N*-tosylBoc amine substituent, forming a single diastereomer (Figure 74d). Spirocyclic substrates were next investigated, but under the same conditions alkene isomerization was observed. This could be addressed by using a phosphoric acid based on BINAM (Figure 74b, right) which gave no isomerization and a product *ee* of up to 87%. Several other substrates worked effectively with the BINAM-derived catalyst (Figure 74e). DFT calculations probed the divergent reactivity between the two chiral acids for the spirocyclic substrates. These suggested that the barrier for reductive elimination was 2.2 kcal/mol lower for the BINAM-phosphate than for the BINOL-phosphate. This also identified a facile alkene isomerization mechanism promoted by the latter, involving a cationic Pd(H) complex.

Sunoj, Toste and co-workers subsequently carried out an in-depth computational investigation into the mechanism of the Heck-Matsuda arylation reaction on spirocyclic substrates, when catalyzed by a BINAM-derived chiral phosphoric acid and Pd(0).^172^ Formation of the diazonium-phosphate ion-pair is needed for the reaction to proceed. After oxidative addition to Pd(0), the sodium cation associated with the deprotonated phosphate is proposed to bridge between the phosphate and a bicarbonate ligand on palladium, which is introduced into the reaction in the form of a sodium carbonate base (Figure 74f). The enantiodetermining step is proposed to be the migratory insertion of the aryl-Pd complex into the bound cycloalkene. During this step, the chiral phosphate is described as a counterion which is bound to sodium bicarbonate, and aryl migration to the *si*-face of the cycloalkene is revealed to be most favorable. This prediction was consistent with the experimentally determined stereochemistry in the product. The calculations predicted several other weak noncovalent interactions between the components at the enantiodetermining transition state, which accounts for the energy difference between *re* and *si*. The authors also modeled the transition state for cycloheptene, a representative nonspirocyclic substrate, which predicted that installation of an alternative *N*-aryl group, specifically 3,5-diisopropoxyphenyl, on the BINAM-derived chiral phosphate would lead to an improvement in enantioselectivity for this class of substrate. This prediction was then validated experimentally.

In 2017, Wu and co-workers reported an enantioselective enyne cycloisomerization for a single substrate under Pd catalysis. A chiral phosphate additive was proposed to be a counterion for a cationic Pd–H complex.^173^ A range of chiral phosphoric acids were evaluated, but enantioselectivity was moderate at best (7–66% *ee*).

**Figure 75:**
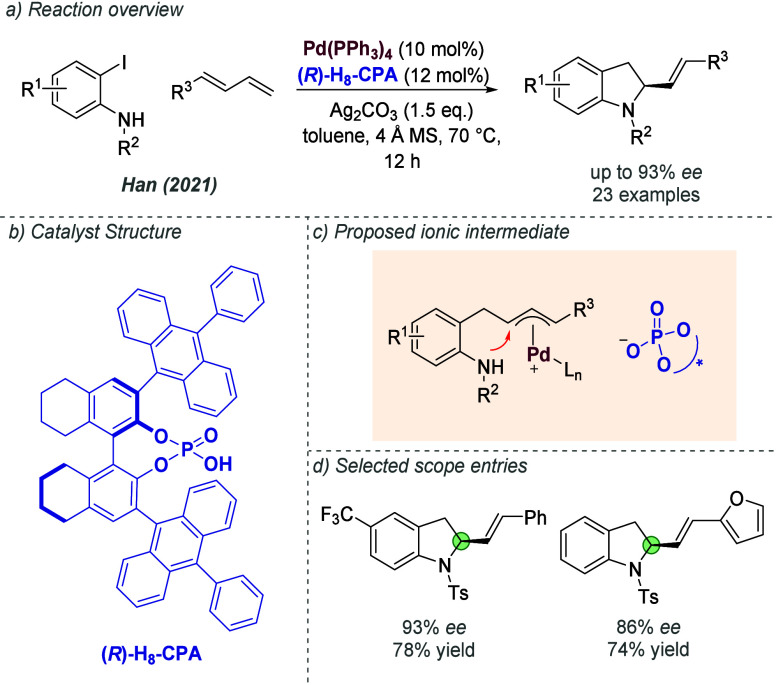
Tandem Heck/Tsuji-Trost reaction of *ortho*-amino aryl iodides and 1,3-dienes using a chiral anion strategy for palladium.

In 2021, Han and co-workers applied the chiral anion strategy for Pd catalysis to the tandem Heck/Tsuji-Trost reaction of *ortho*-amino aryl iodides and 1,3-dienes (Figure 75a).^174^ An H_8_–BINOL based phosphoric acid proved optimal, producing the silver salt of the chiral phosphate *in situ*, enabled by a silver carbonate base (Figure 75b). It was anticipated that this species could then undergo salt metathesis with the aryl-Pd(II)-I intermediate formed after oxidative addition, affording an ion-paired cationic palladium intermediate capable of undergoing migratory insertion into the 1,3-diene (Figure 75c). The chiral anion would then control C–N bond formation, potentially through hydrogen bonding. The authors explored a variety of substituted aryl iodides to give the enriched indole products (Figure 75d). Several 1,3-dienes were also tolerated, giving excellent enantioselectivities. The authors note that this chiral anion strategy proved more general than their previously developed protocol using more conventional chiral phosphoramidite ligands for palladium.^175^

**Figure 76:**
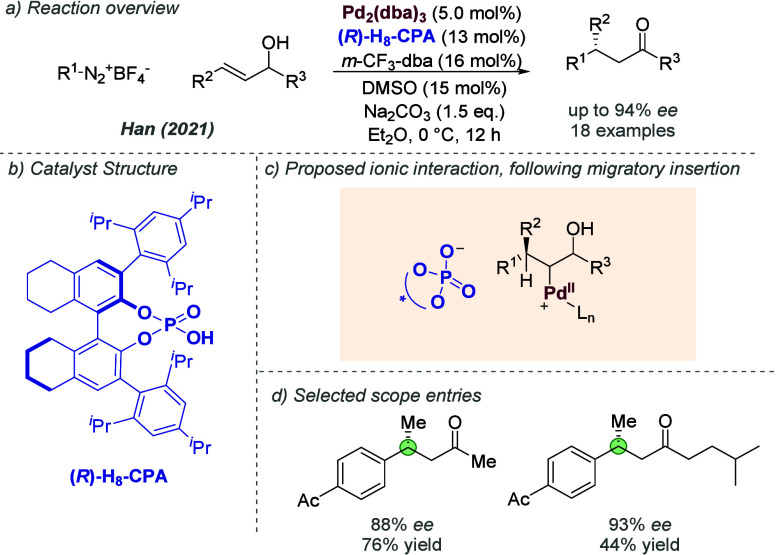
Enantioselective Heck-Matsuda reaction of alkenyl alcohols utilizing a chiral-anion strategy.

The same group also reported an asymmetric Heck-Matsuda reaction of acyclic alkenyl alcohols using the chiral anion strategy for palladium (Figure 76a).^176^ Using a partially hydrogenated chiral phosphoric acid (Figure 76b), a variety of aryl diazonium salts and allylic alcohols were explored, achieving moderate to good yields and excellent enantioselectivities (Figure 76d). As in previous work, migratory insertion is proposed to be enantiodetermining, controlled by the associated chiral phosphate ion of a cationic aryl-Pd(II) complex (Figure 76c).

#### 3.1.3 Iridium

**Figure 77:**
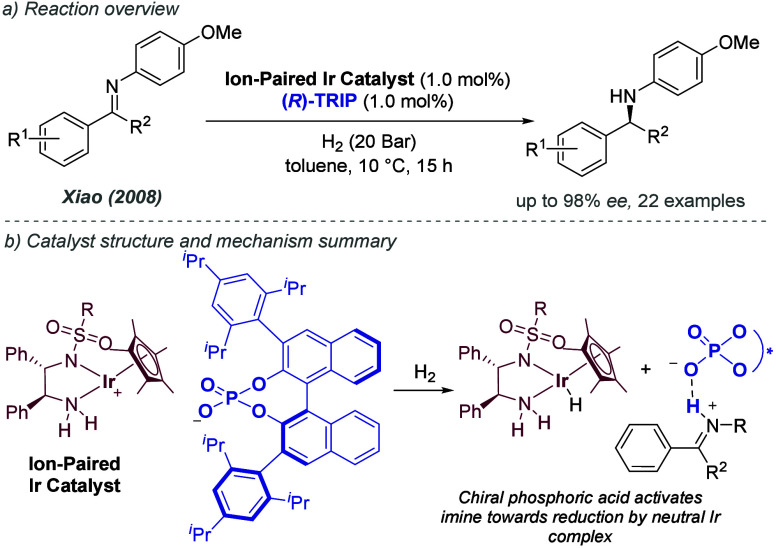
Summary of asymmetric transfer hydrogenation with chiral Ir(III) complex and chiral phosphate anion based on (*R*)-TRIP.

Catalytic cycles featuring cationic iridium complexes also present opportunities for chiral counterion catalysis; the structure of the chiral anion is a readily optimizable variable. This was exploited at an early stage by Xiao and co-workers in a 2008 report involving an Ir(III)-catalyzed asymmetric transfer hydrogenation of acyclic imines (Figure 77a).^177^ Using a Cp*Ir(III) complex with a chiral diamine ligand alone gave no conversion, but addition of the Ir complex in protonated form with TRIP as a chiral phosphate anion gave excellent conversion and 97% *ee*. Mechanistically, the authors propose that upon addition of dihydrogen to the Ir complex, the chiral phosphoric acid is liberated and then protonates the imine (Figure 77b). This in turn activates it toward hydride reduction from the Ir–H complex in a defined chiral environment. A similar pathway was proposed in the follow up study on reductive amination, in which the imine was formed *in situ*.^178^ Subsequent computational studies from Sunoj and co-workers, concerning related chemistry originally developed by Zhao and co-workers (extending this application of hydrogen borrowing),^179^ supported this outline.^180^ Because the transition metal complex is likely not to be involved directly in the ionic interaction during the selectivity-determining reduction step, these processes, and other related reports,^181^ fall outside the defined scope of this Review.^182^

**Figure 78:**
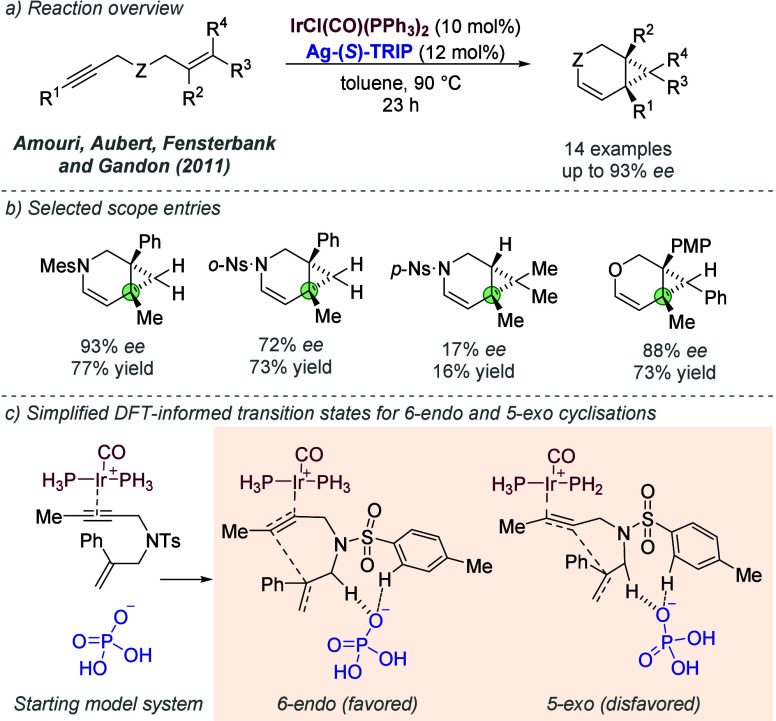
Vaska’s complex bearing a chiral phosphate counterion enables the asymmetric cyclization of 1,6-enyes.

In 2011, Amouri, Aubert, Fensterbank, Gandon and co-workers combined [IrCl(CO)(PPh_3_)_2_] (Vaska’s complex) with the silver salt of TRIP to promote an enantioselective carbocyclization of 1,6-enynes (Figure 78a).^183^ At that time the chiral anion strategy for metals had mostly focused on heterocyclizations, rather than carbocyclizations. Initial optimization gave 81% *ee* and 80% yield for the model substrate (not shown), and subsequent investigation of this methodology on other substrates revealed that both amine- and oxygen-linked 1,6-enynes were tolerated under the reaction conditions (Figure 78b). Interestingly, the heteroatom linker was required to obtain high enantioselectivity. DFT studies on a simplified model system revealed that a 6-*endo* cyclization pathway occurs preferentially to a 5-*exo* pathway (Figure 78c). In both transition states, the phosphate counteranion (albeit with simplified phosphate H_2_PO_4_^–^) is proposed to engage in loose ion pairing with the cationic Ir center, as well as hydrogen bonding with a methylene C–H adjacent to the heteroatom and an *ortho*-positioned hydrogen on the tosyl group. The latter interactions are proposed to provide additional organization at the transition state, presumably playing an important role in enantioinduction when a chiral phosphate is used.

Chen and Hartwig in 2013 reported that use of a bulky phosphate counterion for a cationic Ir complex was able to impact diastereoselectivity in the asymmetric allylation of a prochiral nucleophile (enantioselectivity was not impacted and remained high for various anions).^184^ Moving from tetrafluoroborate to a BINOL-derived phosphate increased dr in the process under investigation from 7:1 to 8:1 to 20:1, in an apparent counterion effect. Preliminary mechanistic studies suggested that the phosphate and methyl carbonate anions (the latter from the leaving group) work together to enhance the diastereoselectivity, but further studies would be required to precisely determine the role of the phosphate.

#### 3.1.4 Manganese

**Figure 79:**
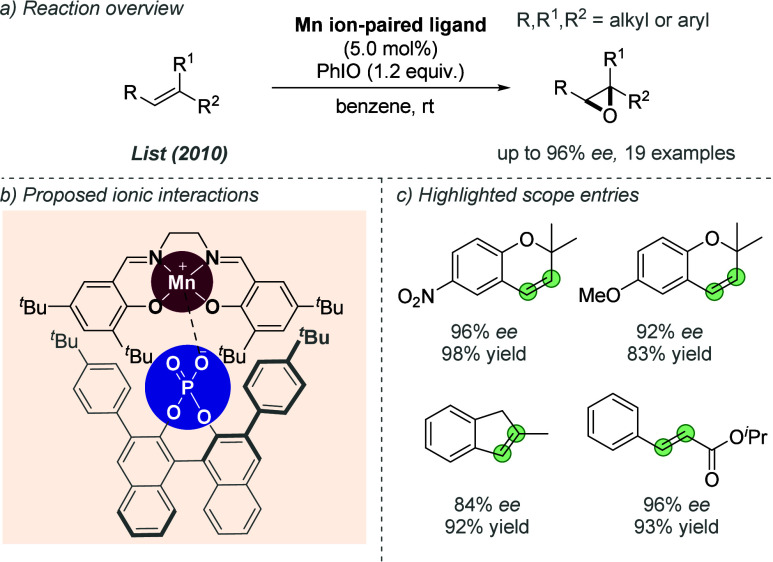
Asymmetric epoxidation of activated alkenes using a cationic manganese-salen complex paired with a chiral BINOL-based phosphate.

In 2010, in an impressive demonstration of how the chiral anion strategy can be applied to familiar catalyst motifs, Liao and List reported that an achiral, manganese salen complex could be ion-paired with a chiral phosphate to enable enantioselective alkene epoxidation (Figure 79a).^185^ The authors took advantage of the fact that Mn(III)-salen complexes can be cationic with a noncoordinating ligand, hypothesizing that if a chiral anion were used, its stereochemical information could alter the conformation of the salen ligand, to fix it in one of two possible enantiomorphic configurations. This would emulate the classic strategy of doing so through a chiral diamine backbone, but without incorporating stereochemical information covalently onto the salen itself. The discrete ion-paired Mn complexes were formed easily from the salen-MnCl complex and a chiral phosphoric acid, in the presence of NaOH. After separately optimizing each catalyst component (Figure 79b), the authors explored the asymmetric epoxidation of a range of cyclic and acyclic substituted styrenes, with very high enantioselectivities for many substrates (Figure 79c).

In 2015, Merten, List and co-workers conducted a detailed study in which they probed the interaction of the cationic metal salen complex and the enantiopure anion using vibrational circular dichroism (VCD) spectroscopy.^186^ They used this technique, which is sensitive not only to chirality but also to changes in conformation and aggregation, to gain insight into the transmission of stereochemical information from the phosphate to the salen. Among other insights, they were able to identify a C=C stretching vibration in the VCD spectra of the ion-paired catalysts that correlated well with the observed enantioselectivity in the product. Chiral anions that gave poorer *ee* appeared less effective in transmitting stereochemical information through the ion-pairing interaction to influence the salen conformation. Overall, they concluded that this was a unique and insightful method for shedding light on the operation of chiral anions, removing some of the mystery associated with these systems.

**Figure 80:**
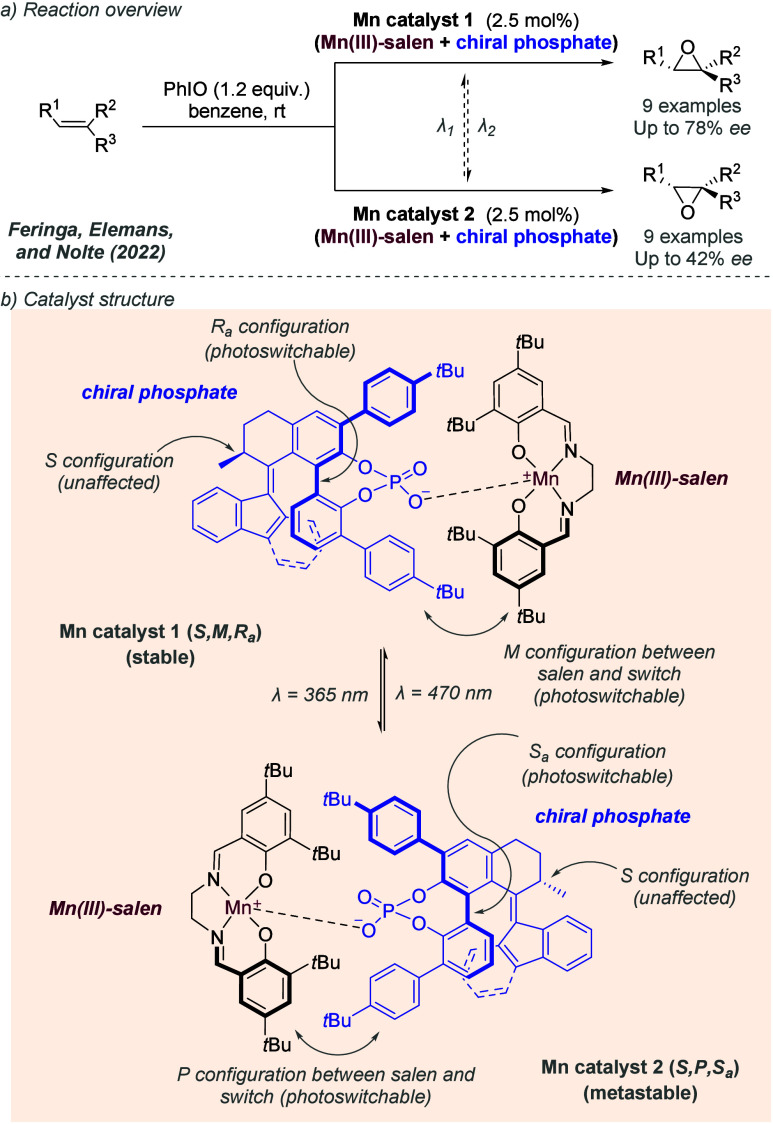
Enantiodivergent alkene epoxidation controlled by a photoswitchable catalyst containing a Mn(III)-salen complex ion paired with a chiral phosphate anion.

In 2022, Feringa, Elemans, Nolte and co-workers developed a version of List’s system which features a photoswitchable chiral phosphate and applied this to enantioselective alkene epoxidation (Figure 80a).^187^ Selectivity for either enantiomer of the epoxide product could be achieved depending on which photoswitchable form of the catalyst was used. The catalyst comprised a Mn(III)-salen complex, which was ion-paired to a chiral phosphate anion (Figure 80b). The ion-paired catalyst contains several chiral elements. These include a point chiral *S*-configured stereocenter on the anion, unaffected during the photoswitching process, but which nevertheless controls the direction of rotation of the catalyst during irradiation; a photoswitchable helically chiral element between the switch and aromatic rings of the salen ligand (*M* or *P*), and a photoswitchable axially chiral biaryl (*R*_*a*_ or *S*_*a*_). Irradiating the more stable form of the catalyst with 365 nm light promoted the conversion to its less stable pseudoenantiomeric form, with the reverse process achievable through irradiation with 470 nm light. Despite being less stable, Mn catalyst 2 underwent no reversion to its more stable form when stored under ambient conditions, including exposure to visible light.

#### 3.1.5 Rhodium

Rhodium is another metal for which the common invocation of cationic complexes offers the opportunity to consider control through ionic interactions with a suitable anion. As in palladium catalysis, the conundrum of anionic ligand or counterion is ever-present and, as stated previously, we shall provide a survey of examples where the authors imply or demonstrate that ionic interactions are at play, since this is a valuable design strategy.

**Figure 81:**
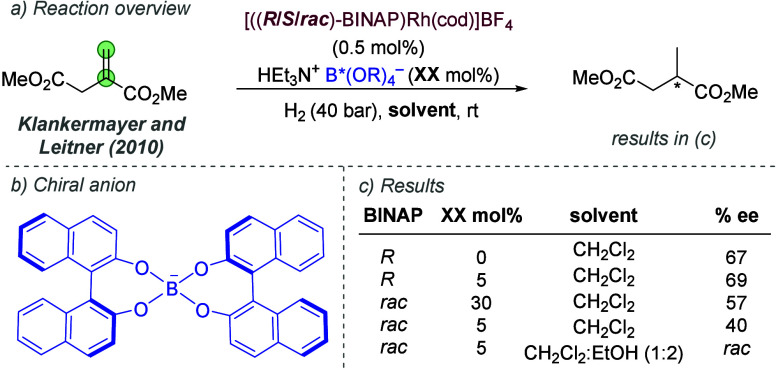
Use of a racemic Rh-BINAP catalyst alongside a chiral borate anion in the enantioselective hydrogenation of dimethyl itaconate.

Leitner, Klankermayer and co-workers in 2010 reported the enantioselective hydrogenation of dimethyl itaconate with a cationic Rh-BINAP catalyst paired with a chiral borate anion derived from BINOL (Figure 81a and b).^188^ Use of an enantiopure BINAP-Rh complex (0.5 mol %) without a chiral anion delivered 67% *ee* in CH_2_Cl_2_, which could be slightly enhanced by addition of 5 mol % of the chiral anion (Figure 81c, entries 1 and 2). Interestingly, if racemic BINAP was used a 57% *ee* could be obtained in the hydrogenation, although 30 mol % of chiral borate was needed to achieve this (Figure 81c, entry 3). When EtOH was added as a cosolvent all enantioselectivity was lost (Figure 81c, entry 4 vs entry 5). Preliminary mechanistic studies suggested that the chiral borate was selectively deactivating the (*R*)-BINAP complex when racemic catalyst was used.

In 2013, Brown and co-workers carried out a very detailed structural (X-ray crystallography) and NMR spectroscopic analysis of ion-paired Rh complexes of the type used by Leitner, Klankermayer and co-workers in their study.^189^ The authors made both diastereomers of these complexes by using either (*R*) or (*S*)-BINAP together with the (*S*,*S*)-borate anion. They were able to deduce that there was an energetic difference between them, and also determined the preferred contacts of individual ion pairs. Interestingly, the ancillary diene ligand on Rh was found to be important; if norbornadiene was used in place of COD then no energy difference was observed. This careful study provides a rare glimpse into the fine detail of interaction between two complex, lipophilic ions in solution and the solid state.

**Figure 82:**
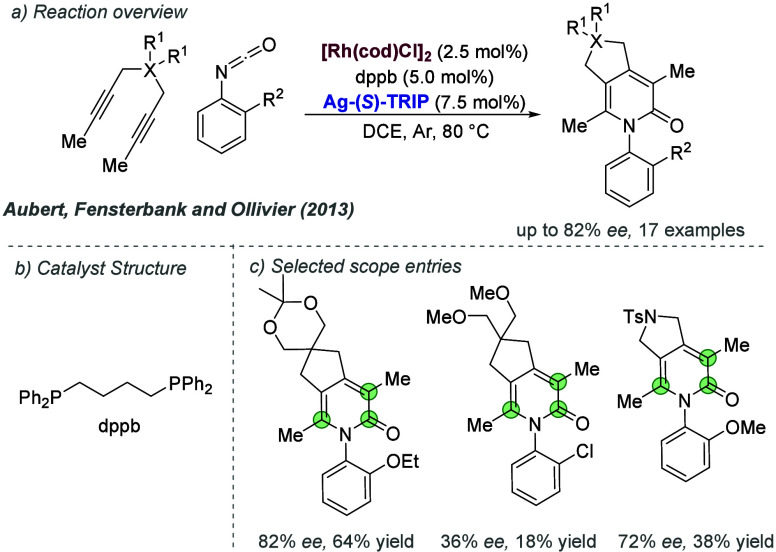
Atroposelective [2+2+2] cycloaddition between diynes and isocyanates catalyzed by a cationic Rh(I)-complex with a chiral counteranion.

Aubert, Fensterbank, Ollivier and co-workers in 2013 investigated atroposelective [2+2+2] cycloadditions between diynes and isocyanates catalyzed by a Rh(I) complex paired with a chiral phosphate, furnishing axially chiral pyridones with up to 82% *ee* (Figure 82a).^190^ (*S*)-TRIP was found to be the optimal chiral phosphate and was combined with [Rh(cod)Cl]_2_ and dppb as an achiral phosphine ligand. Optimization showed that the chiral counterion strategy could give similar levels of *ee* compared with the use of BINAP as the chiral ligand alongside an achiral anion. Substituents on the diyne could include gem-esters, gem-methyl ethers and acetals (Figure 82c), although electron withdrawing groups on the isocyanate were detrimental.

In 2015, the same authors reported a further exploration of their atroposelective [2+2+2] cycloaddition by combining a chiral ligand on the Rh complex with a chiral anion, which outperforms either of these elements when used alone.^191^ After careful optimization, particularly of reaction temperature and prestirring effects, an extensive screen of chiral phosphine ligands allowed the authors to arrive at (*R*)-DM-BINAP, which, when paired with Ag-(*S*)-TRIP, resulted in 88% *ee* on the optimization substrate. In 2016, the same authors completed detailed mechanistic studies involving NMR, mass spectrometry and computation, which explored the counterion effect of Ag-(*S*)-TRIP in the authors’ original report of Rh-catalyzed [2+2+2] cycloaddition.^192^ HMQC experiments could detect no long-range coupling between a potentially ligated phosphate and rhodium. Additionally, neither NOESY nor ROESY could detect through space interactions between the phosphate and other ligands on Rh. DOSY NMR gave different diffusion coefficients for the cation and anion, leading to the conclusion that the interaction between the chiral phosphate and Rh(I) complex is best described as a loose ion pair. Interestingly, the authors found that the discretely prepared complex gives lower *ee* than the *in situ* prepared complex in which the protocol used an excess of Ag-(*S*)-TRIP. This led the authors to discover the formation, under the latter conditions, of a bimetallic Rh–Ag complex that incorporates two TRIP phosphates in a bound manner. It seems that the presence of this complex, which exists in equilibrium with the formerly mentioned species, provides a boost to *ee*, although the origin of this boost is uncertain. This report demonstrates the difficulty in strictly distinguishing ion pairing from ligation of the anion to the metal center.

**Figure 83:**
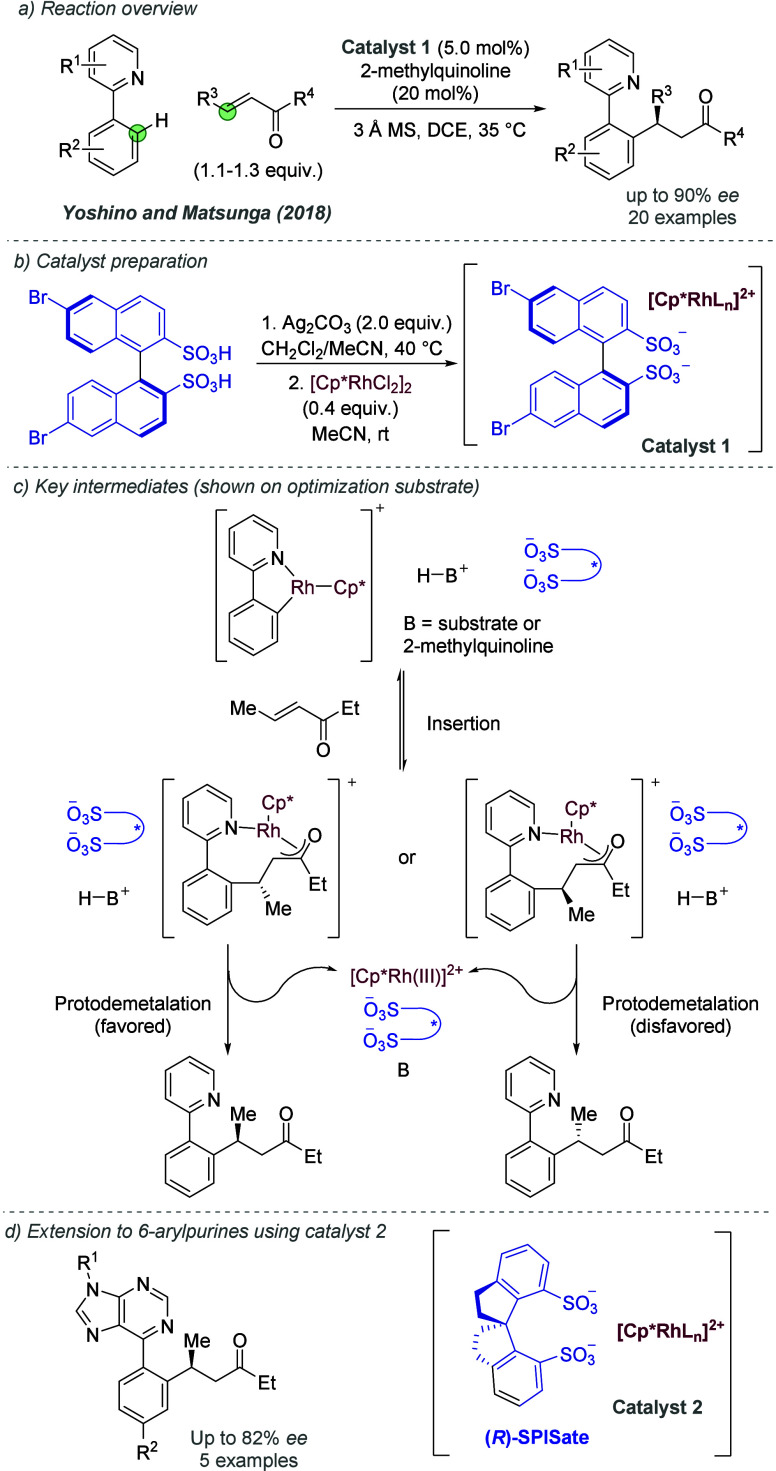
Enantioselective Rh(III)-catalyzed conjugate addition to α,β-unsaturated ketones via C–H activation utilizing chiral disulfonates as proposed anions for Rh.

Yoshino, Matsunaga and co-workers in 2018 reported pentamethylcyclopentadienyl cationic rhodium(III) complexes ion-paired to chiral disulfonic acid anions, for enantioselective C–H bond functionalization (Figure 83a).^193^ The authors envisaged that chiral dianions could serve as alternatives to chiral Cp* ligands that had been prevalent in previous systems, hypothesizing that a bissulfonate dianion would allow partial or complete dissociation from the metal to free up vacant coordination sites and permit C–H activation. The ion-paired catalysts were prepared by the reaction of silver salts of axially chiral disulfonic acids with [Cp*RhCl_2_]_2_ (Figure 83b). These were evaluated in the asymmetric conjugate addition, via C–H activation, of 2-phenylpyridines to α,β-unsaturated ketones; after optimization, high *ee* values could be obtained (Figure 83a). The authors hypothesize that there are two possible mechanisms by which enantioselectivity could arise. The first is an enantiodetermining insertion of the α,β-unsaturated ketone into the Rh–C bond after C–H activation. In the second possibility (depicted in Figure 83c), alkene insertion is reversible and it is protodemetalation of the insertion product that is enantiodetermining. This protonation is carried out by a chiral proton source comprising the protonated base paired with the chiral disulfonate (this proton originated from the earlier C–H activation step). The authors propose that in the first mechanism, enantioselectivity would purely be controlled by ion pairing of the cationic Rh intermediate with the chiral anion. But they discounted this possibility, as there was hardly any effect on enantioselectivity from changing the solvent: more polar solvents should inhibit the formation of a contact ion pair. In the second scenario, it seems plausible that the chiral anion could still engage in an ionic interaction with the intermediate Rh complex while delivering the proton; it is therefore feasible that ion-pairing could still be involved. At the end of the study, a different structure of chiral dianion was used to extend the substrate scope to 6-arylpurines (Figure 83d).

**Figure 84:**
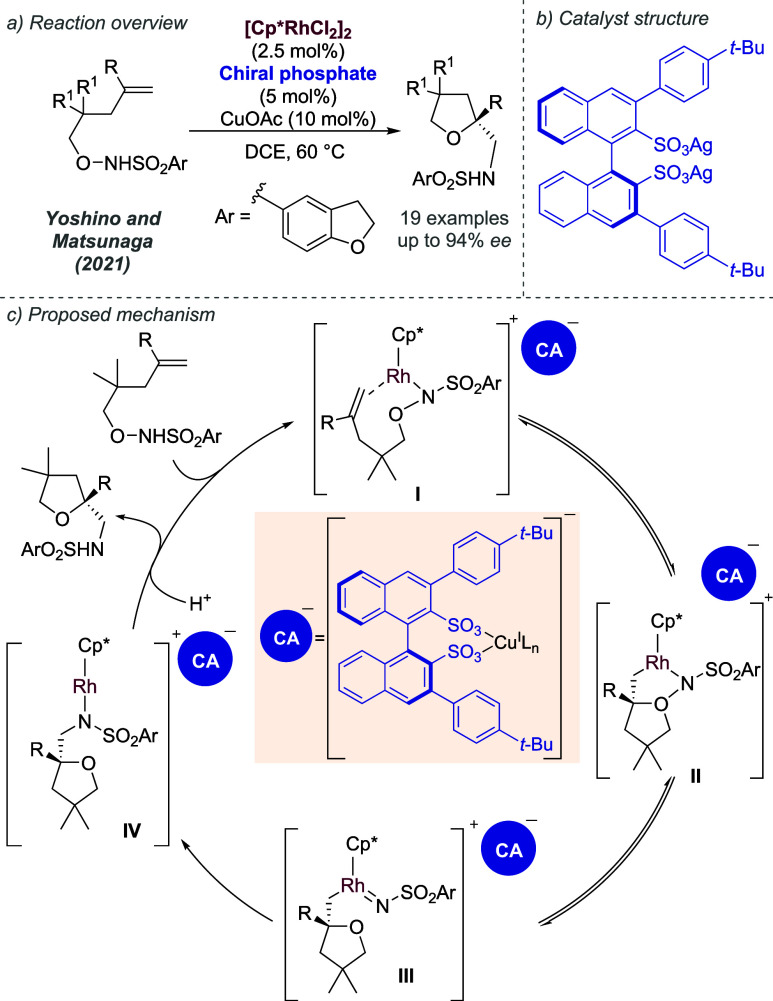
Enantioselective rhodium-catalyzed oxyamination of unsaturated alkoxyamines in which a chiral disulfonate is proposed to act as a chiral anion.

Using similar catalysts, Yoshino, Matsunaga and co-workers subsequently reported an intramolecular oxyamination of unsaturated alkoxyamines to give chiral tetrahydrofurans in high enantioselectivities (Figure 84a,b).^194^ The proposed mechanism begins with the deprotonation and coordination of the alkene to the Cp*Rh complex to give intermediate **I**, which participates in a [3 + 2] cycloaddition to give the cyclized complex **II** (Figure 84c). N–O bond cleavage furnishes the Rh(V) nitrene **III** that undergoes a reductive elimination to give Rh(III) complex **IV**. A final protonation releases the chiral tetrahydrofuran product and regenerates the Cp*Rh catalyst. The Cu(I) cocatalyst was found to be necessary for high enantioselectivity and the authors speculate that the chiral monoanionic Cu-disulfonate complex engages in an ionic interaction with the cationic Cp*Rh complex, and exerts enantiocontrol during the key rate-determining and enantiodetermining reductive elimination step.

#### 3.1.6 Ruthenium

**Figure 85:**
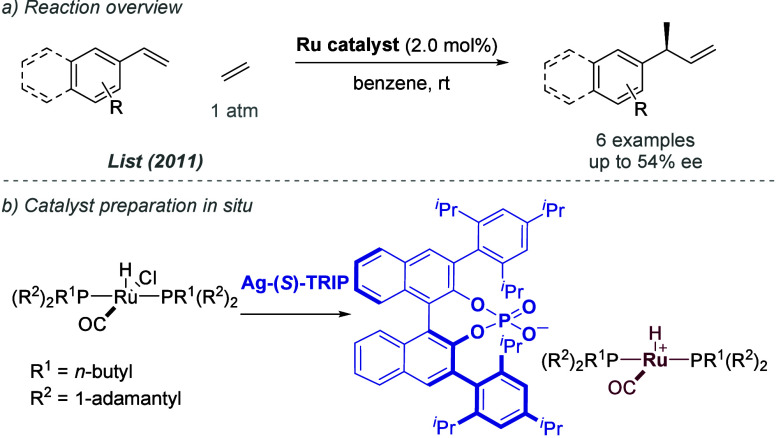
Asymmetric hydrovinylation of styrenes catalyzed by a cationic Ru complex with a chiral phosphate counteranion.

In 2011, Jiang and List described an asymmetric Ru-catalyzed hydrovinylation of styrenes directed by a chiral phosphate (Figure 85).^195^ The authors initially attempted this with cationic nickel complexes, as they are well precedented to promote this reactivity, but no enantioselectivity was obtained. They switched metal to ruthenium–previous counterion effects had been observed in nonasymmetric processes, so the authors were optimistic that a chiral anion could impact selectivity. A combination of Ru(CO)Cl(PCy_3_)_2_H and Ag-(*S*)-TRIP was found to promote the enantioselective hydrovinylation of styrene with ethene under mild conditions, affording the product in 46% *ee* with no evidence of isomerization. This could be slightly increased to 54% by modifying the Ru catalyst using a more bespoke phospine (Figure 85b). The scope of the reaction included five further examples with different substituents on the styrene, affording moderate enantioselectivities (34–44% *ee*).

The Krische group were early in seeing the potential of using chiral BINOL-derived phosphoric acids to augment selectivity in transition metal catalyzed processes. Very soon after the seminal reports of Akiyama and Terada, which used these in organocatalytic processes,^196^ Komanduri and Krische discovered that a chiral (*R*)-TRIP catalyst could promote the enantioselective reductive coupling of 1,3-enynes and heterocyclic aldehydes under Rh(I) catalysis, with Rh bearing achiral ligands.^197^ The authors suggested that the role of the acid is to protonate the heterocyclic substrate, rather than engage in ion pairing with Rh. This was based on analogous experiments with nonbasic substrates, which were found not to give any enantioselectivity. Nevertheless, the combination of the Bro̷nsted acid with a metal complex bearing a noncoordinating counterion (in this case triflate) showed foresight. In 2012, Krische and co-workers revisited this strategy in the enantioselective C–H crotylation of alcohols via hydrohydroxyalkylation of butadienes.^198^ In this work a combination of 5 mol % of a chiral Rh complex, ligated by dppf, and 10 mol % of a chiral phosphoric acid gave product in excellent dr and *ee*. In the proposed mechanism for the reaction, the chiral phosphate controls both regioselectivity, by partitioning *E* and *Z* isomers of the intermediate σ-crotyl complex, as well as enantioselectivity arising from addition to the aldehyde. Throughout the proposed mechanism the chiral phosphate is depicted as a coordinating ligand bound to the ruthenium, with no ionic interactions proposed, and so it technically falls outside the scope of this Review. This assessment of the phosphate acting as a ligand for Rh was supported by subsequent detailed DFT calculations probing the origins of selectivity in the reaction from Grayson, Krische and Houk.^199^ Interestingly, the same authors also discovered that the opposite diastereomer and enantiomer could be obtained selectively by moving away from BINOL-derived phosphates and using TADDOL derived phosphates combined with SEGPhos, a chiral phosphine ligand.^200^

**Figure 86:**
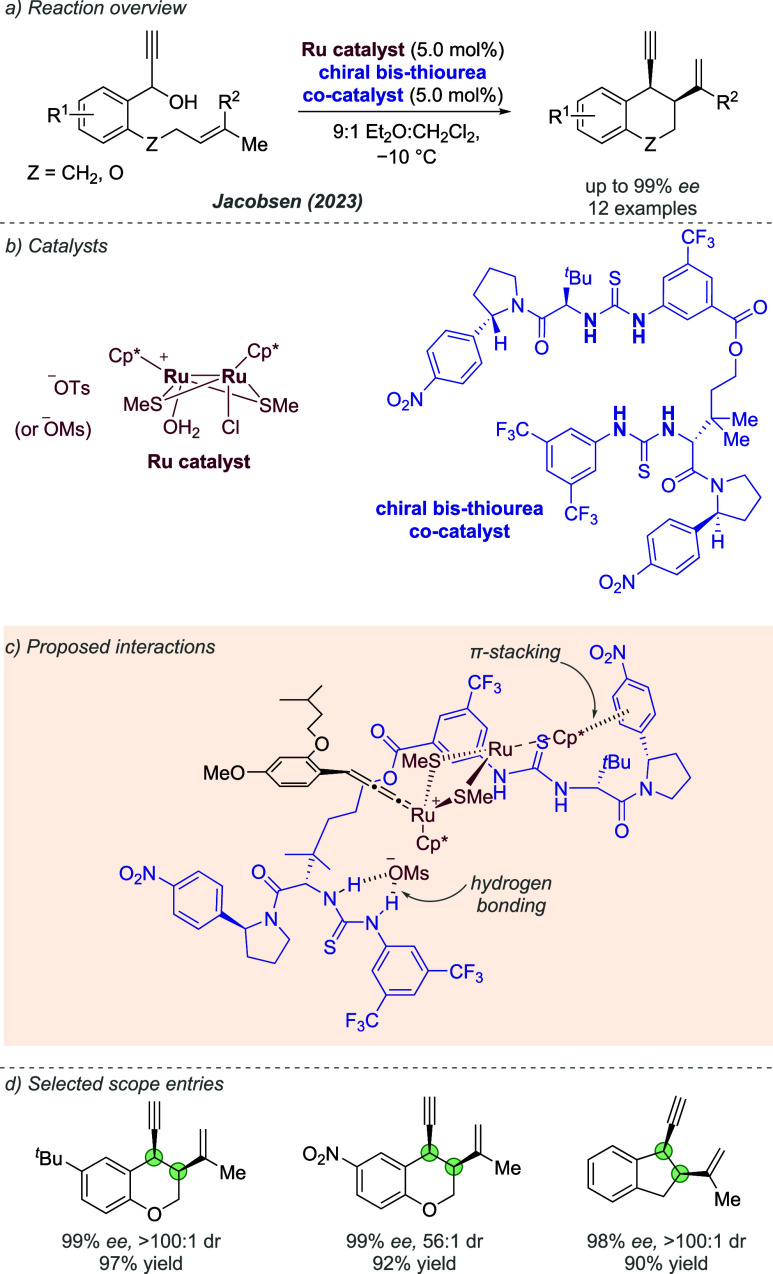
Enantioselective Ru-catalyzed propargylation via the binding of an achiral anion of a Ru complex to a chiral hydrogen bond donor catalyst.

In 2023, Jacobsen and co-workers reported an asymmetric Ru-catalyzed propargylic substitution, in which asymmetry is induced by way of a chiral hydrogen bond donor catalyst binding the anion of an achiral Ru complex (Figure 86a).^201^ These reactions proceed via cationic Ru-allenylidene intermediates which can then react with nucleophiles. Unlike previous examples discussed in this chapter, which use a chiral anion to engage in ionic interactions with a cationic metal center, in this work the anion is itself achiral but is rendered effectively chiral through strong binding with an added chiral hydrogen bond donor catalyst. Optimization studies showed that an ionic diruthenium tosylate complex could give encouraging product *ee* when combined with a mono thiourea catalyst; this could be increased markedly by switching to a bis-thiourea which contains four separate stereocenters on the backbone (Figure 86b, right). Optimization using the latter resulted in excellent yield, dr and *ee* in the cyclized product, and control experiments demonstrated that both catalytic components were required to obtain appreciable conversion. A substrate scope was demonstrated, with various substituents tolerated on the aromatic ring (Figure 86d). Other classes of alkenyl propargylic alcohols also worked well, enabling formation of tetralin and indane examples in a highly diastereoselective and enantioselective manner. Various counterions of the Ru complex gave moderate to high *ee* in the reaction (OTs or OMs were optimal) but, in contrast, weakly coordinating BAr^F^_4_ resulted in racemic product. Detailed NMR studies compared and contrasted OTs and BAr^F^_4_ salts of a presumed intermediate in the reaction that lacked the alkene (and could therefore be studied because it would not undergo further reaction). No changes in the NMR spectra were observed in the BAr^F^_4_ salt or catalyst upon mixing, in contrast to significant changes with the OTs salt, leading to the conclusion that if no anion binding occurs, no selectivity can be achieved. ROESY NMR studies confirmed the binding of a mesylate anion within the catalyst, and a systematic solvent evaluation showed the importance of nonpolar solvents for effective enantioinduction, suggesting tight ion-pairing is required (Figure 86c). Additionally, kinetic studies were conducted which provided insight on the importance of substitution on the 2-arylpyrrolidine portion of the catalyst. The authors were able to advance a ROESY-informed DFT model, in which low energy conformations of the transition state showed attractive noncovalent interactions between the electron deficient arene at the terminus of the hydrogen bond donor catalyst, and Cp* ligands on the Ru.

#### 3.1.7 Cobalt

**Figure 87:**
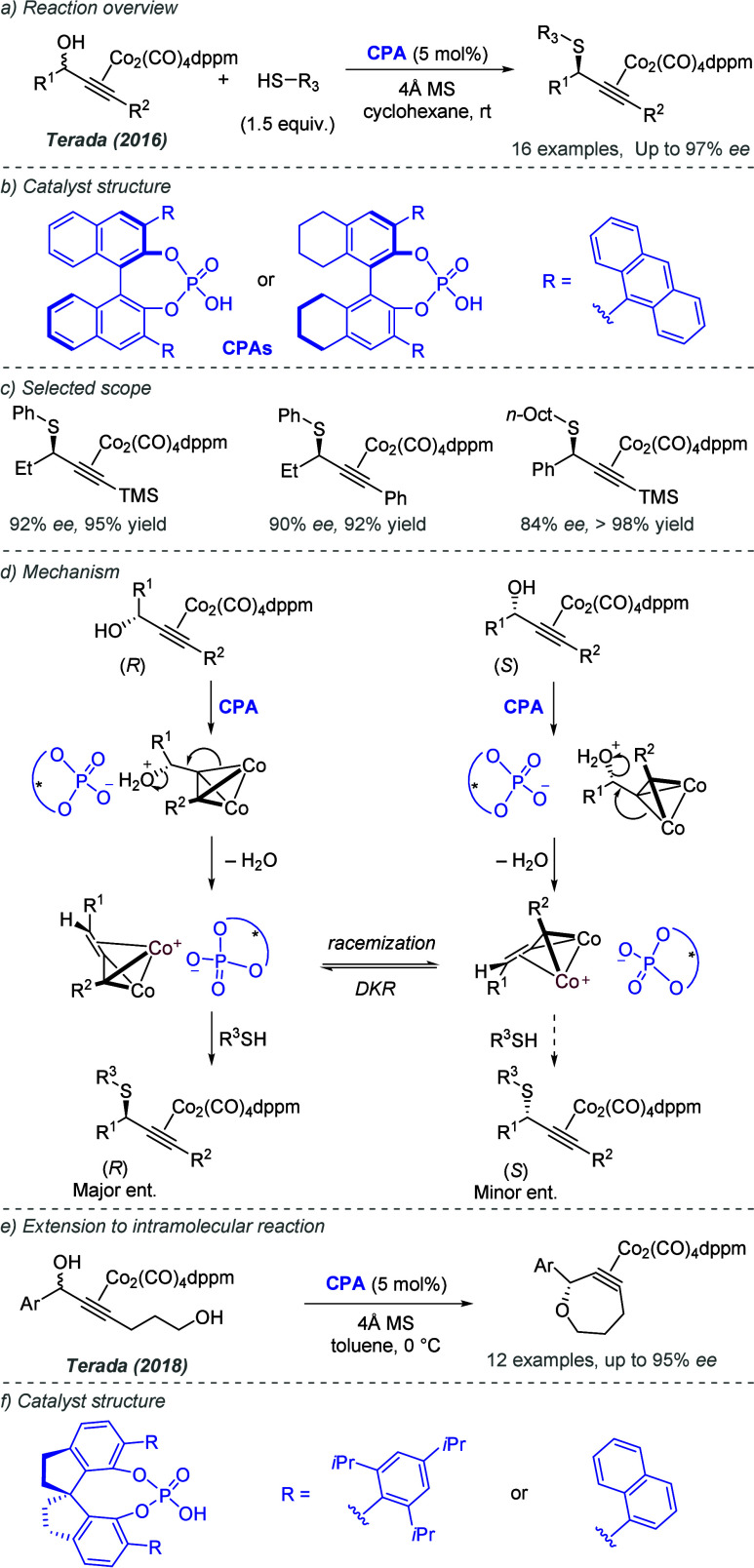
Enantioconvergent Nicholas reaction using a chiral phosphoric acid catalyst via dynamic kinetic resolution.

In 2016, Terada and co-workers reported an enantioconvergent Nicholas reaction, whereby racemic propargylic alcohols, bound to a dicobalt complex, were converted to the corresponding enantioenriched propargylic thiols (Figure 87a).^202^ The reaction was catalyzed by a chiral phosphoric acid derived either from BINOL or H_8_–BINOL (Figure 87b). Optimization of the reaction conditions was undertaken on an alkyl substituted secondary alcohol, TMS-substituted alkyne, and a thiophenol nucleophile, delivering the product in excellent yield and enantioselectivity (Figure 87c, left). While investigating the scope of the reaction, the authors uncovered several significant variations. A phenyl substituent was well tolerated on the alkyne (Figure 87c, middle), as were primary and secondary alkyl substituents (not shown). A benzylic alcohol was also a good substrate, while an aliphatic thiol could be used as the nucleophile with only a small reduction in enantioselectivity compared to thiophenol (for a representative example, see Figure 87c, right). The authors carried out mechanistic experiments, with their proposed mechanism shown in Figure 87d. Protonation of the alcohol by the chiral phosphoric acid followed by dissociation of water generates two enantiomeric cationic cobalt species, which can reversibly racemize. While paired with the chiral anion of the CPA, one of the diastereomeric ion-paired species reacts with the thiol nucleophile faster than the other, in a dynamic kinetic resolution. In 2018, Terada and co-workers extended this work to an intramolecular process (Figure 87e).^203^ The alkyne was substituted with an alkyl chain attached to a nucleophilic alcohol, which could intercept the electrophilic intermediate to form an enantioenriched cyclic ether. For this reaction, optimal results were obtained with chiral phosphoric acids derived from SPINOL (Figure 87f). Unlike the authors’ earlier work, alkyl-substituted secondary alcohols afforded poor enantioselectivity, requiring an aryl or heteroaryl substituent in this position (c.f. Figure 87c, left and center).

#### 3.1.8 Copper

**Figure 88:**
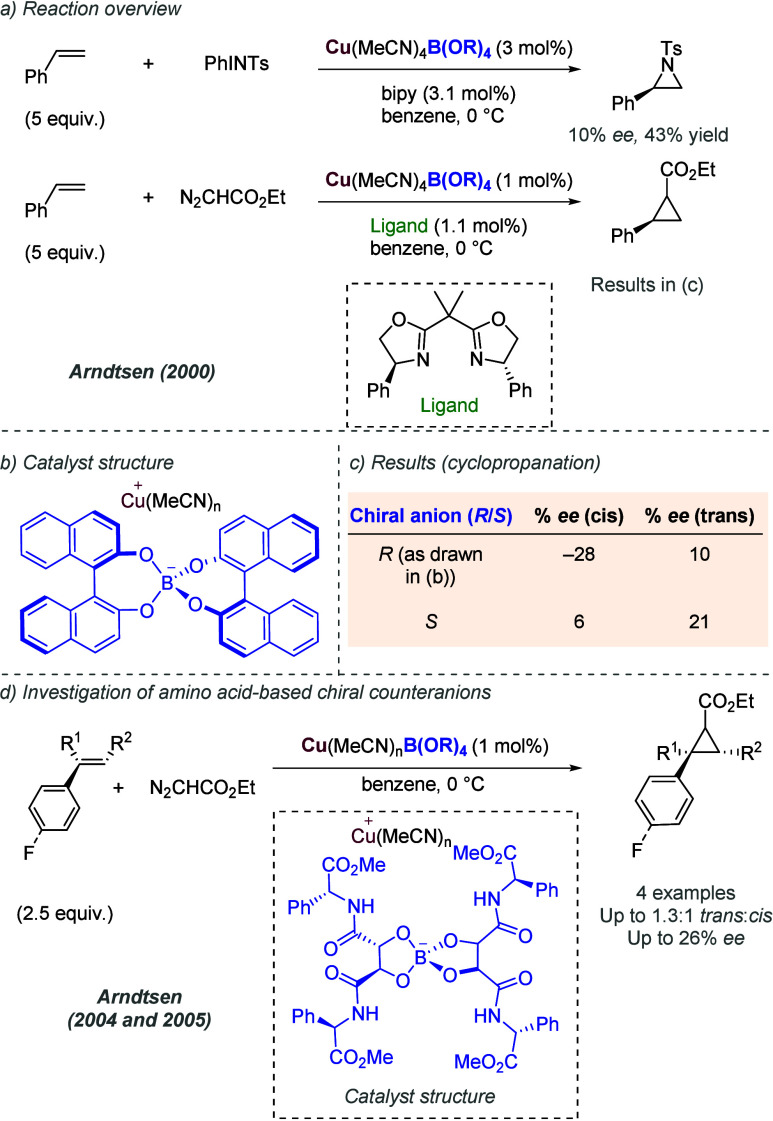
Early examples of enantioselective aziridation and cyclopropanation using a cationic copper catalyst ion paired with a chiral borate anion.

Arndtsen and co-workers contributed early examples of pairing cationic metals with counteranions to control enantioselectivity.^204^ While *ee* in these early examples was low, they provided important proofs-of-concept that laid the foundations for later advances. Their first report described the synthesis of a chiral, BINOL-derived borate anion (Figure 88b) and its application in copper-catalyzed aziridination and cyclopropanation of styrene (Figure 88a). Initially investigating aziridination, nonzero *ee* (7%) could be obtained when using this chiral borate in the absence of a chiral ligand for copper, which could be increased to 10% *ee* upon the addition of achiral 2,2′-bipyridine (Figure 88a, top). This was taken as evidence that the chiral anion was influencing enantioselectivity through ion pairing, rather than ligation to the metal. For cyclopropanation, it was found that when a chiral ligand was used in conjunction with the chiral anion, a significant matched/mismatched effect was observed (Figure 88a, bottom and Figure 88c). Later, Llewellyn and Arndtsen also investigated α-amino acid–based designs for chiral borate anions (Figure 88d).^205^ Twenty chiral ion-paired catalysts, which combined chiral elements from tartaric acid as well as amino acids, were prepared. For the substrate scope, a catalyst derived from (*R*,*R*)-tartaric acid and an unnatural amino acid with an (*R*)-Ph substituted side chain was used, affording four cyclopropanes in up to 26% *ee*.

A more recent study from the same group demonstrates that care must be taken to ensure that chiral anions do not undergo structural changes under the reaction conditions, which may give misleading outcomes. A chiral BINOL-based difluoroborate was synthesized and ion-paired with a copper(I) species for evaluation in an alkene cyclopropanation reaction with diazocarbonyls.^206^ Without additional ligand added, low enantiomeric excess (8% *ee*) was observed, but addition of various achiral phosphine ligands resulted in promising enantioselectivities being obtained (up to 70% *ee*). Upon further analysis of the reaction, it was clear that reproducibility was poor and, through ^11^B and ^19^F NMR analysis, they realized that the originally added chiral anion had undergone modification during the reaction. The authors proposed that this releases the substituted BINOL, which can then complex oxidized copper to form an effective chiral catalyst, albeit one not based on ion pairing.

**Figure 89:**
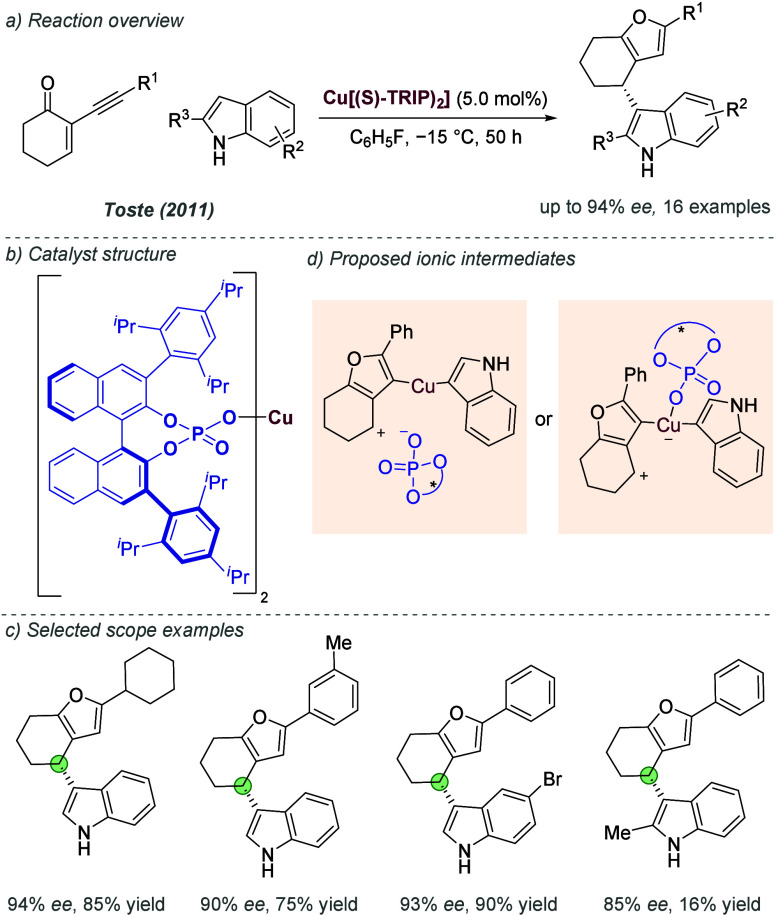
Enantioselective Cu(II)-catalyzed cycloisomerization-addition of indoles to enynones, in which a chiral phosphate potentially ion pairs with a key cationic intermediate.

In 2011, Toste and co-workers reported a Cu(II)-catalyzed cycloisomerization-addition reaction of enynones with indoles to give highly substituted furan products (Figure 89a).^207^ The optimal catalyst for the process was a Cu(II) complex in which the copper is ligated by two BINOL-derived phosphates (Figure 89b), which gave 91% *ee* for the optimization substate (not shown). Various steric and electronic modifications were tolerated on the enynone substrate, including alkyl substituents on the terminal alkyne position and indole substitution (Figure 89c). Following a series of careful mechanistic experiments the authors proposed that the active catalyst consists of a copper monophosphate bound to indole at the 3-position (not shown). Copper can then interact with the enynone via alkyne binding, prompting addition of the carbonyl to form a cuprated furan, with an adjacent carbocation (Figure 89d, left). The chiral phosphate that was on the copper can potentially ion pair with this carbocation, to control the addition of a separate incoming indole nucleophile (i.e., not the indole from the active catalyst). It could alternatively act as an anionic ligand for copper to form an anionic cuprate species, directing the subsequent nucleophilic attack (Figure 89d, right). Protodemetalation then closes the catalytic cycle. In this work the phosphate is proposed to be acting as an anionic ligand for copper, rather than a counterion, although ionic interactions do still feature in one of the possible mechanistic scenarios (Figure 89d, left).

**Figure 90:**
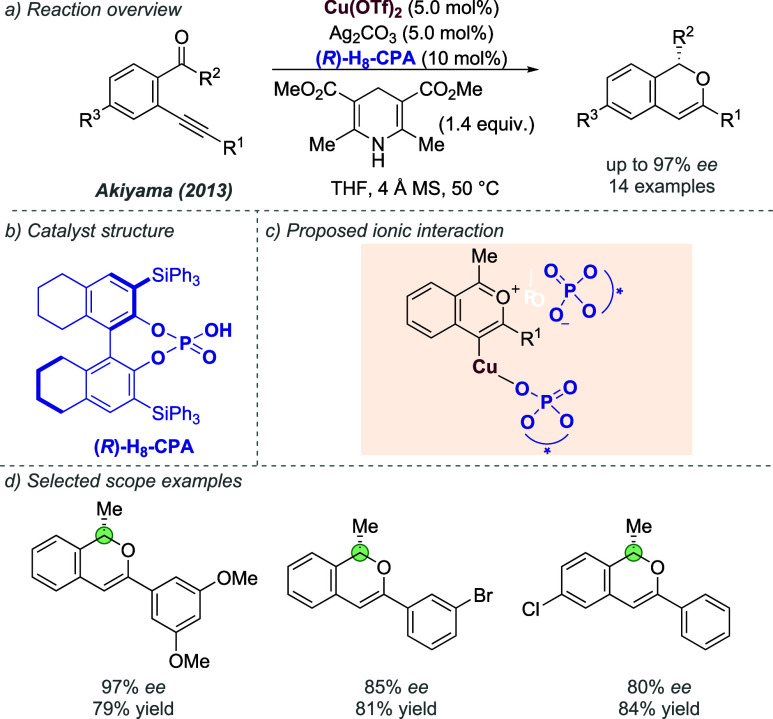
Enantioselective Cu(II)-catalyzed cyclization-transfer hydrogenation of *o*-alkynylacetophenones, proposed to proceed via an ion-paired intermediate.

In a mechanistically related transformation, in 2013 Akiyama and co-workers reported a Cu(II) catalyzed enantioselective cyclization and transfer hydrogenation to access chiral isochromenes from *o*-alkynylacetophenones, through a cyclization/reduction sequence (Figure 90a).^208^ Optimization of the chiral phosphate structure showed that SiPh_3_ at the 3,3′-positions of the BINOL gave best enantioselectivity (Figure 90b). The scope of the reaction was explored, including variation of the alkyne substituent and the aryl ring (Figure 90d). A copper diphosphate salt, formed *in situ*, is presumed to be the active catalyst. The authors propose a mechanism in which intramolecular cyclization takes place once the copper diphosphate complex binds to the alkyne. This allows carbonyl addition to form a cuprated oxonium ion that is ion-paired with a chiral anion (Figure 90c). This anion can direct the transfer hydrogenation by a Hantzsch ester in the final enantiodetermining step.

**Figure 91:**
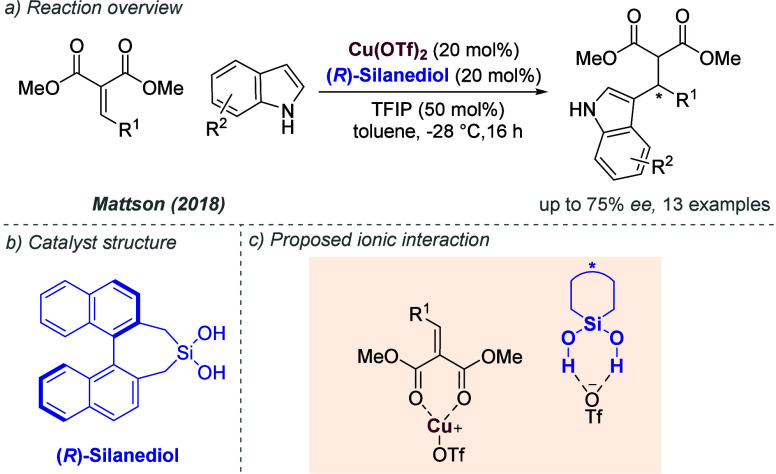
Enantioselective Cu(II)-catalyzed addition of indoles to alkylidene malonates using a silanediol catalyst as a potential anion binder.

Mattson and co-workers in 2018 reported an enantioselective conjugate addition of indoles to alkylidene malonates using a Cu(II) salt as a Lewis acid, in combination with a chiral silanediol catalyst (Figure 91a).^209^ The authors began by combining the BINOL-derived silanediol shown with a variety of transition metal salts (Figure 91b). Cu(OTf)_2_ worked best, giving up to 75% *ee* on the optimization substate. A 1,1,1-trifluoroisopropanol (TFIP) additive was also found to improve reactivity. The authors evaluated a range of variously substituted indoles and malonates with *ee* values ranging between 30% and 86%. Several other common hydrogen bond donor catalysts were investigated and the silanediol was found to be uniquely effective in delivering both reactivity and enantioselectivity. The authors tentatively hypothesize that the silanediol could activate the Cu(OTf)_2_ by binding one of the anionic triflates to enhance Lewis acidity (Figure 91c). The ensuing addition of the indole can take place within the chiral environment conferred by the chiral silanediol, now complexed to the triflate anion, which can form an ion pair with the copper complex.

#### 3.1.9 Silver

There are several examples of silver catalysis which are thought to proceed by mechanisms similar to those covered in the gold and copper catalysis sections. In these examples too, there is the possibility of inducing asymmetry using chiral anions.

**Figure 92:**
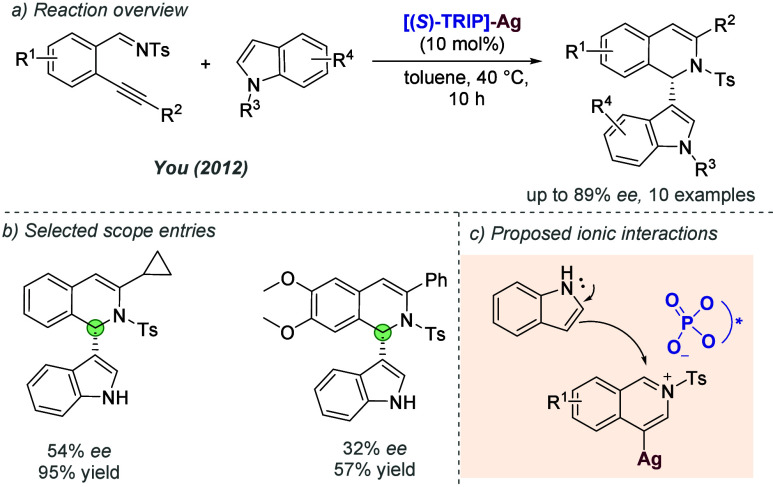
Silver(I) phosphate catalyzed cycloisomerization/addition to access chiral 1,2-dihydroisoquinolines.

In 2012, You and co-workers reported a silver catalyzed cyclization/addition of *o*-alkynylacetophenone imines with indoles (Figure 92a).^210^ The silver salt of TRIP was identified as the optimal catalyst; this was applied to a range of substrates, with moderate to high enantioselectivities obtained (Figure 92b). As in related transformations, an ion pair was proposed to form between the chiral phosphate and the cationic iminium intermediate formed after cyclization, which still contains the silver metal (Figure 92c). This interaction was proposed to control the subsequent indole addition.

**Figure 93:**
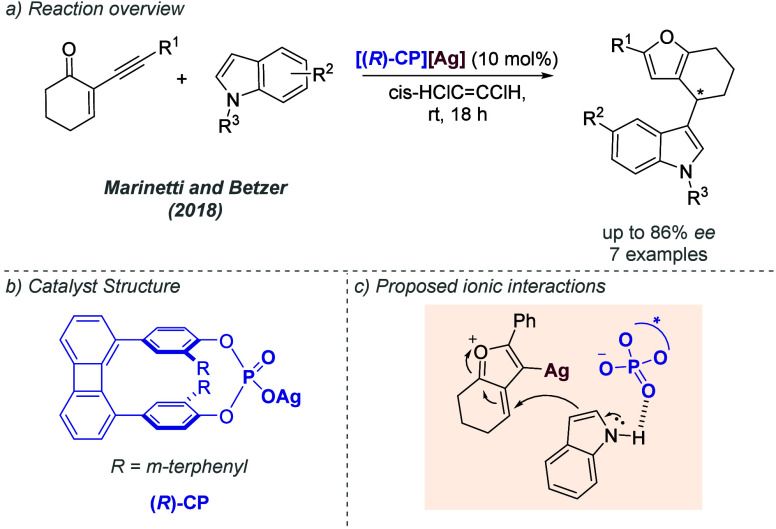
Enantioselective silver phosphate-catalyzed tandem cycloisomerization/addition of enynones and indoles.

In 2018, Marinetti, Betzer and co-workers reported a cycloisomerization/addition of enynones and indoles using chiral paracylophane-based silver phosphates (Figure 93a).^211^ The optimal catalyst was based on 1,8-biphenylenediyl tethered silver phosphate (Figure 93b), giving 86% *ee* for the cycloisomerization/addition of the model enynone substrate and indole after optimization. During scope exploration, the authors discovered that *N*-methylindole gave a lower 39% *ee* and 27% yield, suggesting that the indole N–H is acting as a hydrogen bond donor during the enantiodetermining indole addition to the cationic intermediate (Figure 93c).

#### 3.1.10 Iron

There are relatively few examples with iron in which ionic interactions are invoked. The work of Pappo and co-workers, in which chiral phosphates are used as anionic lignds for iron, was discussed at the beginning of Section 3.1. Because detailed studies suggested that the phosphate remains fully bound during the key steps of the catalytic cycle, these will not be covered further here.

**Figure 94:**
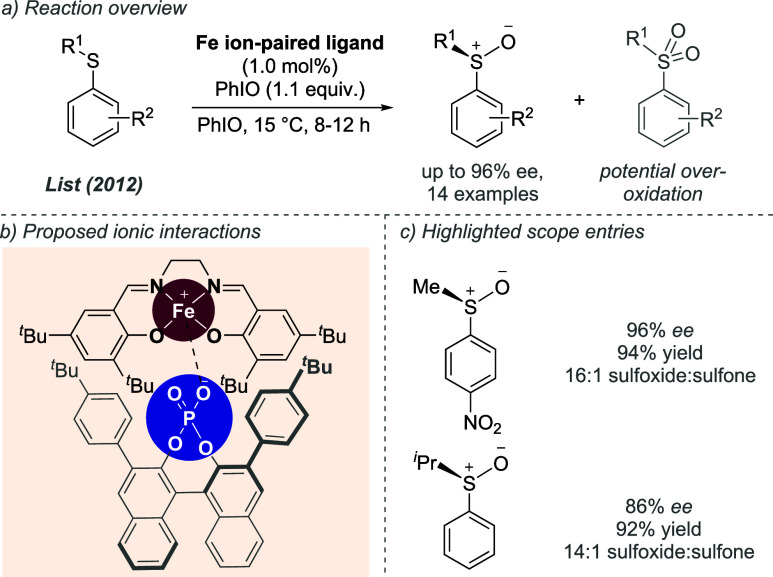
Enantioselective sulfoxidation using an achiral Fe*-*salen complex paired with a chiral phosphate counterion.

Having previously applied the chiral counterion strategy to Mn complexes bearing achiral salen ligands for epoxidation, Liao and List reported an exploration of this strategy on Fe-salen complexes, to enable enantioselective sulfoxidation (Figure 94a).^212^ Achiral cationic metal salen complexes were prepared with Mn, Fe, Cr, and Co. This was followed by ion exchange with the chiral BINOL-derived phosphate that had been optimal in the authors’ previous work. The Mn complex gave 50% *ee* in the oxidation of phenylmethylsulfide, as well as significant overoxidation. In contrast, the Fe complex gave 60% *ee* and less overoxidation; further optimization of catalyst loading and temperature increased this to 70% *ee* (the Cr and Co complexes gave inferior outcomes). A variety of electron-donating and electron-withdrawing groups were tolerated on the aryl ring, giving enantioselectivities of up to 96% *ee*. Variation of the alkyl group on sulfur was also investigated using a *para*-nitrophenyl substituted arene. The authors proposed a model for the substrate trajectory in approaching the iron oxo intermediate, which is related to their previous work with the Mn salen.^185^

### 3.2 Anionic Metal Complexes with a Countercation

In comparison with the many examples discussed in the preceding section, where cationic metals were combined with chiral anions, there are rather fewer examples of the charge-inverted approach whereby anionic metal complexes are combined with chiral cations. It seems logical that this should be the case: it is less common to encounter anionic metal complexes at key points in catalytic cycles where selectivity may be influenced. Nevertheless, there is a handful of innovative applications of this approach, which will be covered in the following section. Within the broader context of chiral cations being used in conjunction with palladium catalysis, there are several notable examples where asymmetric phase-transfer catalysis, generating a chiral, ion-paired nucleophile, is combined with palladium-catalyzed allylic alkylation.^213^ These will not be discussed in detail here: we specified in the scope of this Review that “the ionic interaction should involve the transition metal complex itself in some way.”

#### 3.2.1 Manganese

**Figure 95:**
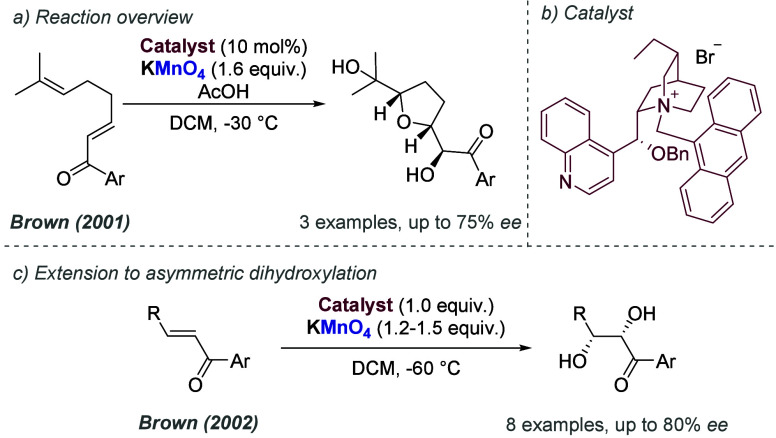
Early enantioselective oxidations with permanganate using a dihydrocinchonidine-based chiral cation as a phase transfer catalyst.

Potassium permanganate is an established strong oxidant, capable of oxidizing various functional groups. Although this is routinely carried out in aqueous solutions or in mixtures with water-miscible organic solvents (a challenging medium for controlling selectivity through ion pairing due to the high polarity of water), MnO_4_^–^ is anionic and so offers the potential for reactivity tuning via the associated cation. Use of phase-transfer agents together with KMnO_4_ has extended its applicability by enabling such oxidations to be carried out in nonpolar solvents.^214^ This feature opens the possibility of rendering permanganate-mediated oxidations enantioselective when chiral phase-transfer catalysts are used. An important early example of enantioselective permanganate-mediated oxidation was reported by Brown and Keily in 2001 (Figure 95a).^215^ Using a dihydrocinchonidine-based chiral cation as a phase-transfer catalyst, the authors performed an oxidative cyclization of 1,5-dienes to access 2,5-bis(hydroxymethyl)-substituted tetrahydrofurans with three new stereocenters created in a single step, albeit with moderate enantioselectivities (Figure 95a and b**)**. Brown and co-workers subsequently developed a permanganate-promoted asymmetric dihydroxylation of enones, with stoichiometric amounts of the same catalyst as the phase-transfer agent (Figure 95c).^216^ Unfortunately, the authors observed decomposition of the chiral cinchonidinium salt under the dihydroxylation conditions, which complicated their goal of rendering the reaction catalytic with respect to the chiral cation salt.

**Figure 96:**
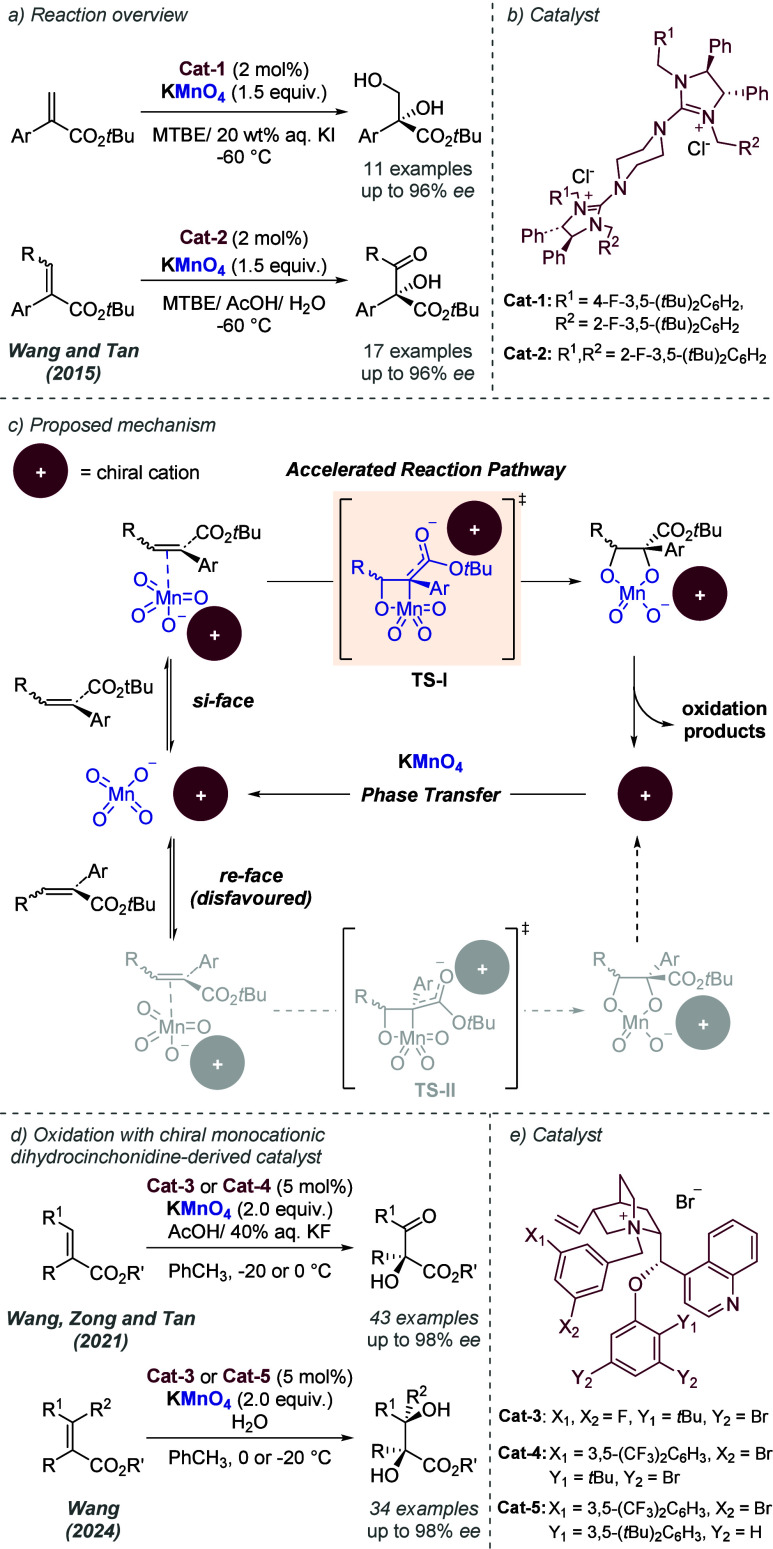
Enantioselective oxidation of α-aryl acrylates with KMnO_4_ using a chiral dicationic bisguanidinium catalyst, and oxidation of α-alkyl enoates using a chiral monocationic dihydrocinchonidine-derived catalyst.

In 2015, Wang, Tan and co-workers built on this early work with an enantioselective oxidation of α-aryl acrylates with KMnO_4_, using a chiral dicationic bisguanidinium salt^217^ as a phase-transfer catalyst.^218^ Under the optimized reaction conditions, various α-aryl acrylates were dihydroxylated with high enantioselectivities (Figure 96a, upper). Furthermore, in the presence of an acetic acid additive, mixtures of *E* and *Z*-trisubstituted α,β-unsaturated esters were oxidized to the same enantiomer of the corresponding oxo-hydroxylated products with high enantioselectivities (Figure 96a, lower). In the proposed catalytic cycle the authors envisage that the chiral catalyst forms an ion pair with the permanganate anion to accelerate *si*-face oxidation of the alkene via the lower-energy transition state **TS-I** (Figure 96c). Stabilization of the transition state was attributed to a shorter interionic distance between the enolate anion and the chiral cation in **TS-I**, resulting in a proposed rate enhancement. More recently, Wang, Zong, Tan and co-workers demonstrated that monocationic cinchonine-derived chiral cations could also function effectively, using this finding to expand the scope of their asymmetric oxohydroxylation reaction (Figure 96d, upper and Figure 96e).^219^ Wang, Zong, and co-workers also applied this to the synthesis of bicalutamide derivatives for possible medicinal chemistry applications.^220^ Wang and co-workers later explored the permanganate-promoted dihydroxylation reaction using similar cations (Figure 96d, lower).^221^

#### 3.2.2 Tungsten

**Figure 97:**
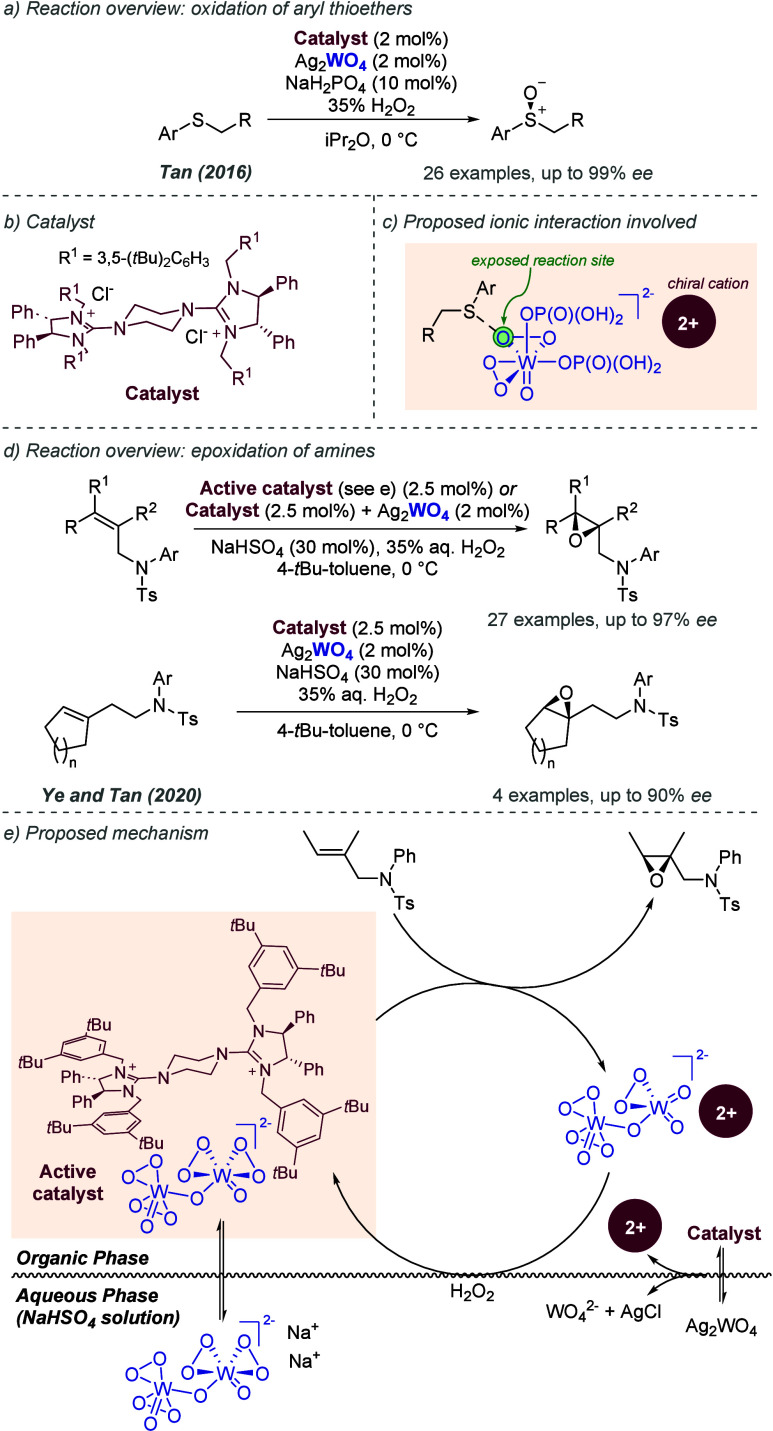
Enantioselective oxidation of aryl thioethers and tungstate-catalyzed epoxidation of amines with chiral bisguanidinium catalysts.

In 2016, Tan and co-workers reported an enantioselective tungstate-catalyzed oxidation of aryl thioethers to chiral sulfoxides with good to excellent enantioselectivities (Figure 97a).^222^ Through Raman spectroscopy and computational studies, the authors proposed that the active disphosphatobisperoxotungstate anion is embedded within the chiral cavity of the bisguanidinium dication (Figure 97b,c). As a result, this orientation exposes only one of the peroxo-oxygen atoms, and restricts the direction of nucleophilic attack by the aryl thioether. The reaction is proposed to occur through a phase transfer mechanism. A range of benzimidazole-derived sulfides were oxidized with high enantioselectivity, and it was shown that the benzimidazole could be changed to other heteroarenes and simple arenes while still maintaining high *ee*. Following this report, Ye, Tan and co-workers applied this catalyst system to the enantioselective tungstate-catalyzed epoxidation of allylic and homoallylic amines under phase-transfer conditions (Figure 97d).^223^ The authors were able to prepare and characterize the active ion-paired catalyst by X-ray crystallography, infrared (IR) and Raman spectroscopies; in particular, the X-ray structure of this species revealed that the achiral tetraperoxyditungstate anion resides in the central cavity of the chiral bisguanidinium dication, as previously proposed. Furthermore, the authors noted the importance of NaHSO_4_ in the reaction, which they speculate is integral for maintaining the structure of the active catalyst, as well as enabling catalytic turnover with hydrogen peroxide. Mechanistically, the authors proposed that the tungstate anion is oxidized and undergoes dimerization in the presence of NaHSO_4_ and oxidant to give the tetraperoxyditungstate anion (Figure 97e). Ion pairing with the chiral bisguanidinium dication furnishes the active ion-paired catalyst, which carries out the enantioselective epoxidation of the alkene substrate.

**Figure 98:**
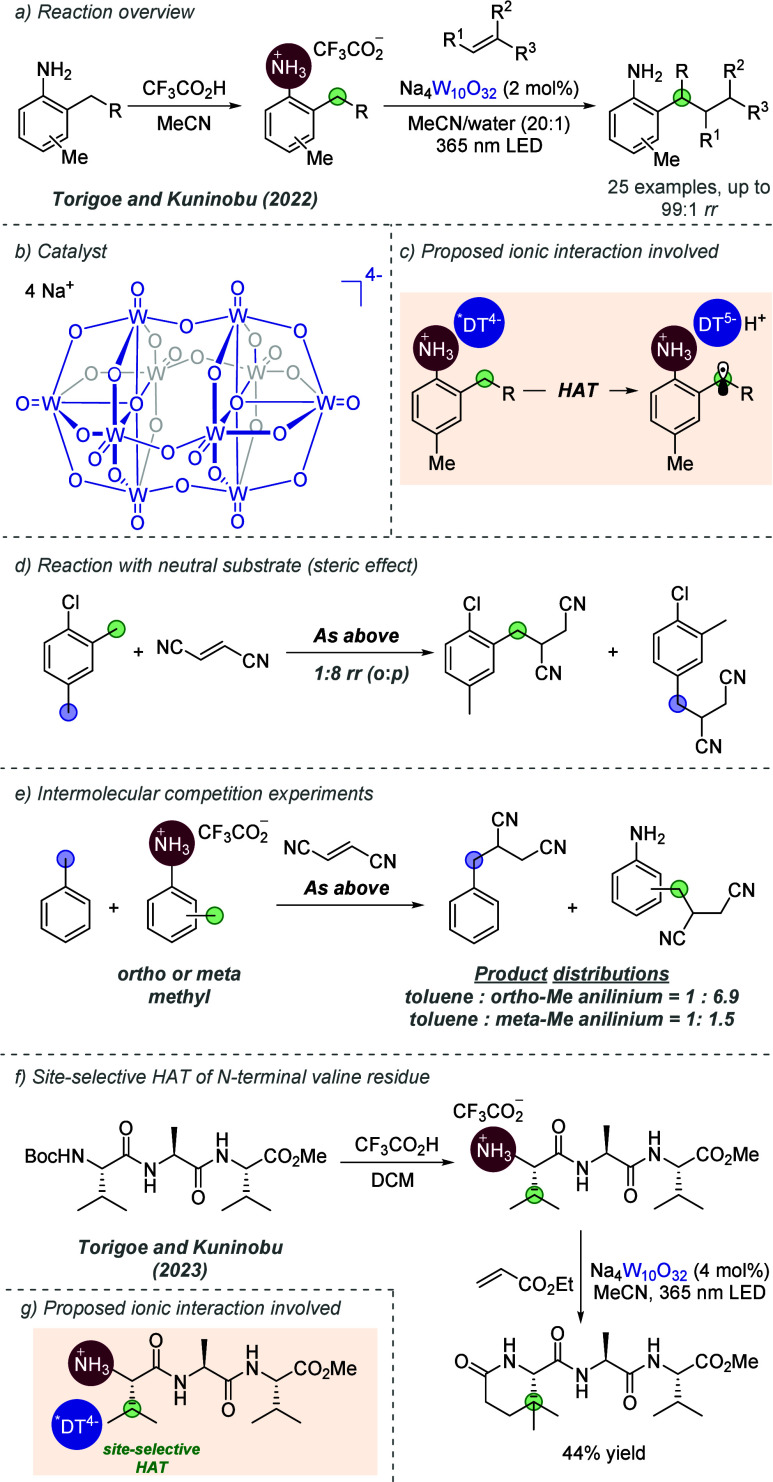
Site-selective HAT directed by electrostatic interactions between a cationic anilinium and anionic decatungstate photocatalyst.

Photocatalytic radical approaches toward C–H functionalization have gained momentum in recent years, with the use of hydrogen atom transfer (HAT) as one of the key steps in exerting site-selective control over the C–H bond cleavage event. Specifically, the photoexcited decatungstate [W_10_O_32_]^4–^ anion represents a versatile HAT catalyst, which is able to selectively abstract the most sterically accessible and electron-rich C(*sp*^*3*^)-H bond.^224^ Moreover, its anionic charged nature is of particular interest to this section of the Review, due to the opportunities of harnessing ion pairing interactions in controlling site-selective radical C–H functionalization. In 2022, Zeng, Torigoe and Kuninobu reported a site-selective C(*sp*^*3*^)-H alkylation of 2-methyl anilinium salts utilizing photoexcited sodium decatungstate as the hydrogen atom transfer (HAT) catalyst (Figure 98a).^225^ The authors envisioned that by leveraging the electrostatic interactions between the positively charged ammonium group on the substrate and the negatively charged, UV-excited decatungstate, HAT could be directed toward the proximal methyl group as opposed to a distal one (Figure 98c). Indeed, good to excellent site-selectivities for functionalization of the methyl at the *ortho* position were achieved. As a comparison, the authors carried out the alkylation of neutral 1-chloro-2,4-dimethylbenzene and observed selective functionalization of the methyl group at the *para* position (Figure 98d). Furthermore, intermolecular competition experiments with toluene showed significant alkylation of the *ortho*-methyl anilinium substrate, despite its expected lower reactivity due to its more electron-deficient nature (Figure 98e). Using a similar strategy, Song, Torigoe and Kuninobu recently demonstrated that electrostatic interactions between a positively charged ammonium group at the *N*-terminus of a Val-Ala-Val tripeptide and the anionic decatungstate photocatalyst can be harnessed to achieve selective HAT at the *N*-terminal Val residue (Figure 98f,g).^226^ Several examples were demonstrated where this occurs with very high site-selectivity in peptides containing two Val residues. This work, which demonstrated control over site-selectivity, built also on prior observations from Britton and co-workers relating to rate acceleration of HAT in leucine derivatives, in which a similar electrostatic interaction was proposed.^227^

#### 3.2.3 Molybdenum

**Figure 99:**
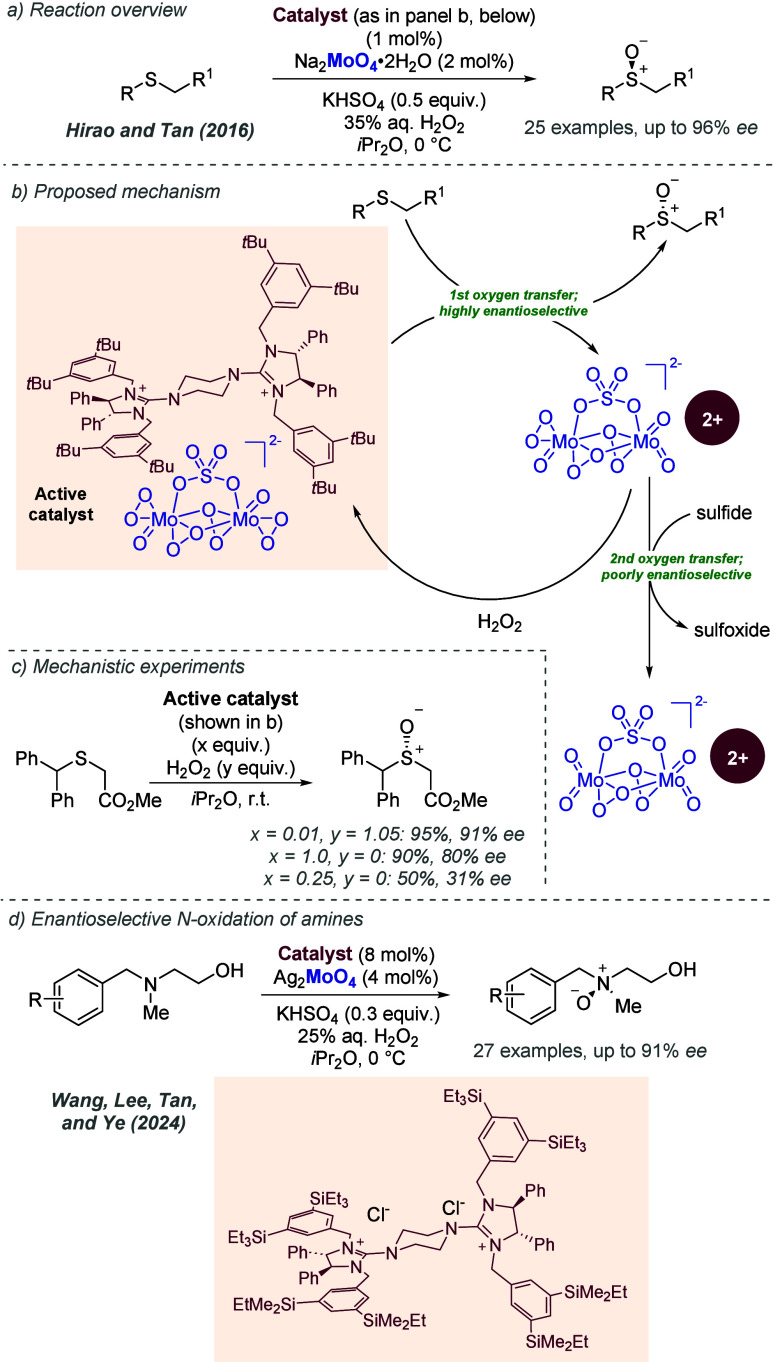
Enantioselective oxodiperoxomolybdosulfate-catalyzed oxidation of thioethers to chiral sulfoxides and amines to *N*-oxides, directed by a bisguanidinium dication.

In 2016, Hirao, Tan and co-workers reported an enantioselective oxidation of thioethers to chiral sulfoxides, using an ion-paired bisguanidinium oxodiperoxomolybdosulfate catalyst (Figure 99a).^228^ Structural elucidation of the active catalyst (Figure 99b) through various spectroscopic techniques revealed that the achiral anionic dinuclear oxodiperoxomolybdosulfate is embedded within the chiral framework of the bisguanidinium dication, providing a chiral environment in which oxygen transfer can occur. To provide support for this depicted complex being the active catalyst, the authors tested it with stoichiometric H_2_O_2_ in the oxidation of methyl 2-(benzhydrylsulfanyl)acetate and obtained comparable reaction metrics to when the active catalyst is generated *in situ* (Figure 99c). The authors also demonstrated that this species, in the absence of H_2_O_2_, oxidizes methyl 2-(benzhydrylsulfanyl)acetate to its corresponding sulfoxide in 90% yield and 80% *ee.* However, when the loading of this species was lowered (0.25 equiv), the sulfoxide was obtained in 50% yield with a substantial drop in enantioselectivity to 31% *ee*. This suggests that two out of the four peroxo moieties in the active catalyst are capable of effecting oxygen transfer, as two catalytic turnovers were observed in this experiment. Taking these results together, the authors proposed that this species catalyzes the enantioselective oxidation of the substrate to the chiral sulfoxide, and this first oxygen transfer is highly enantioselective (Figure 99b).^217^ In the absence of H_2_O_2_, the reduced molybdenum species is capable of effecting a second, poorly enantioselective oxidation of the substrate. However, under the optimized reaction conditions, H_2_O_2_ ensures catalyst turnover and maintains the dimeric structure of the catalyst, which is crucial for effective enantiofacial discrimination. Zong and co-workers subsequently extended this methodology toward the enantioselective oxidation of cyclic sulfides, using a more sterically confined bisguanidinium dication.^229^

In 2024, Wang, Lee, Tan and Ye applied their ion-paired molybdosulfate catalyst system to the asymmetric *N*-oxidation of amines.^230^ This work featured a similar catalyst to that reported previously, but it was found that use of a silver molybdate salt afforded better results than the sodium salt. Further optimization of the chiral cation structure allowed very high *ee* to be obtained for oxidation of a prochiral tertiary amine bearing methyl, benzyl and a substituent bearing a β-hydroxyl group (Figure 99e). A wide variety of substitution of the benzene ring was tolerated, although variation of the other two amine substituents was detrimental to enantioselectivity for acyclic amines. Notably, the protocol could be expanded to cyclic tertiary amines. An X-ray crystal structure was obtained showing the oxidized molybdate salt embedded in the chiral cavity provided by the bisguanidinium cation.

#### 3.2.4 Silver

**Figure 100:**
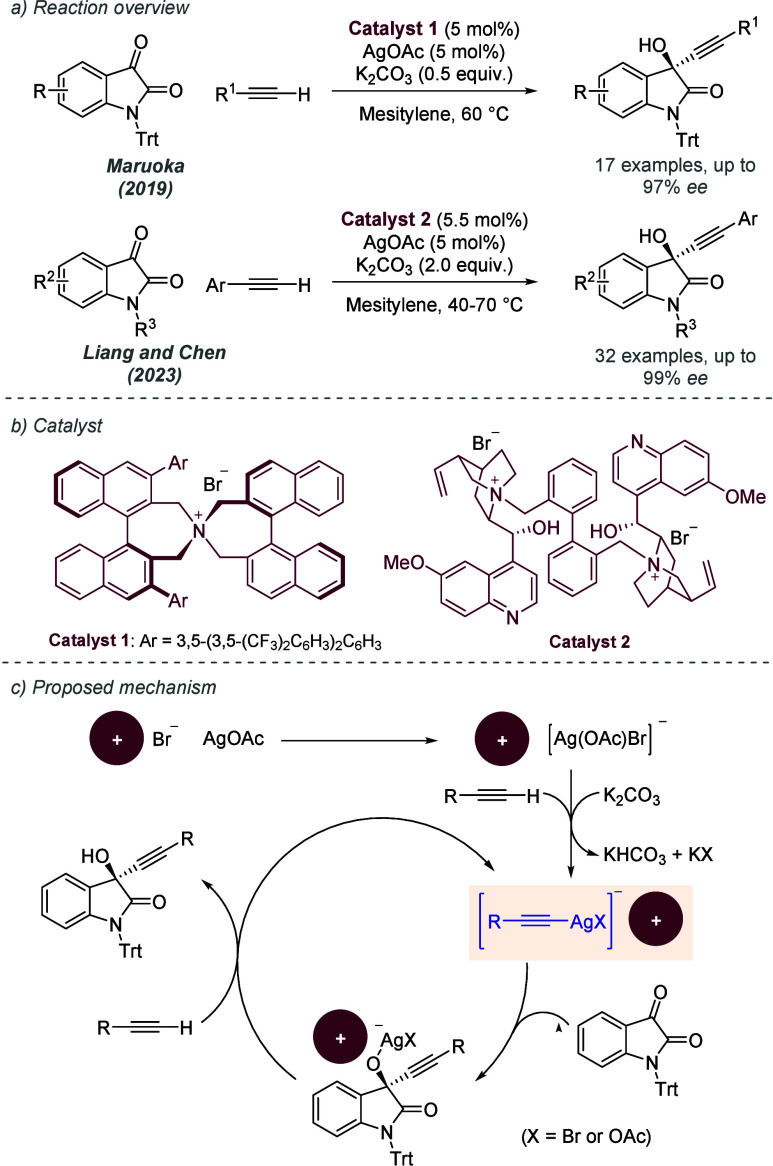
Enantioselective silver-catalyzed alkynylation of isatin derivatives directed by a chiral cation.

In 2019, Maruoka and co-workers reported an enantioselective, silver-catalyzed alkynylation of isatin derivatives using chiral cationic phase transfer catalysts (Figure 100a, upper).^231^ The authors proposed that the chiral cation and AgOAc would react to give an achiral anionic silver complex which is paired with the chiral cation (Figure 100c, upper). Deprotonation of the terminal alkyne results in the formation of an anionic silver alkynylide, ion-paired with the chiral cation (Figure 100c). This then adds to the isatin in a highly enantioselective manner which, upon protonation by another molecule of alkyne, furnishes the product and closes the catalytic cycle for silver. Following this report, Liang, Chen and co-workers reported a similar enantioselective, silver-catalyzed alkynylation of isatins, using a bis-quaternized dication derived from quinine (Figure 100a, lower).^232^

**Figure 101:**
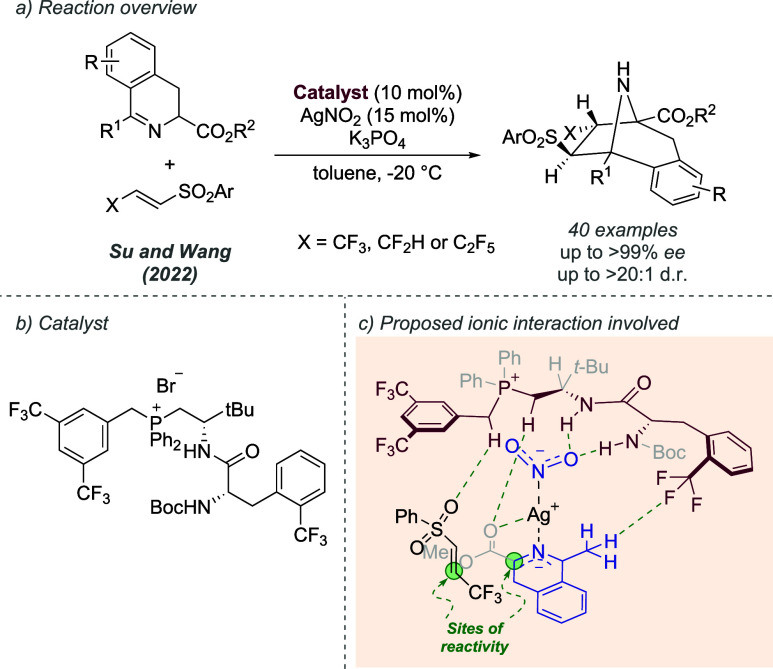
Enantioselective synthesis of *N*-bridged [3.2.1] tropane-like systems enabled by chiral phosphonium-silver catalysis.

In 2022, Su, Wang and co-workers demonstrated that *N*-bridged [3.2.1] cyclic systems could be accessed from cyclic azomethine ylides and β-fluoroalkylvinylsulfones under silver and chiral phosphonium catalysis (Figure 101a).^233^ Central to the success of this methodology was the use of a chiral, bifunctional, dipeptide phosphonium salt, such that chiral tropane-like products, bearing four contiguous stereocenters, were obtained in good to excellent enantioselectivities and excellent diastereoselectivities. Based on results obtained from experimental and computational mechanistic investigations, the authors proposed that following deprotonation, the cyclic azomethine ylide is activated upon coordination to the Lewis acidic Ag(I) cation (Figure 101c). Simultaneously, the chiral phosphonium salt engages in hydrogen bonding with the nitrite anion, as well as forming various noncovalent interactions with both substrates. This network of noncovalent interactions preorganizes the system, resulting in the cyclization occurring with high stereoselectivity.

## 4 Summary and Outlook

This Review aims to provide a detailed survey of how ionic interactions have been used in the design of transition metal catalysts to exert selectivity control and how these strategies have been applied. The emphasis has been placed on system design, and ambiguities regarding whether or not counterions coordinate to metals have been acknowledged.

Section 2 explored examples where this interaction occurs in the outer ligand sphere. In many cases these ligand designs are rooted in existing well-explored metal–ligand complexes, which are already known to be catalytically active in the reaction type in question. Innovation has arisen from the careful introduction of ionic functionality into the ligand periphery, such that an attractive interaction can be obtained either with the substrate, to guide subsequent reaction, or with a chiral counterion, to render the ligand chiral. These strategies have the advantage that existing state of the art ligands that have been developed to give the highest reactivity in a given process can be modified in the outer sphere to only minimally impact that carefully curated reactivity, while enabling influence on selectivity in the ensuing reaction. In some of these cases, it is likely that covalently appending chiral information directly onto the ligand scaffold would negatively impact reactivity. Associating a chiral anion or cation in the outer ligand sphere offers an unconventional way to render the complex chiral; while these strategies are still relatively few, this Review has shown that they can be highly effective. A point of caution is that as a ligand scaffold becomes more elaborate, the barrier for practicing chemists to use it increases: the beneficial effect must justify the catalyst’s complexity.

The outer sphere approach parallels enzyme catalysis, albeit in a simpler manner–it is not just selectivity that can be influenced but also reaction rates. Although the latter aspect is not specifically considered in this Review, its benefits are not to be underestimated when challenging catalytic processes need encouragement. Although enantioselective processes form the bulk of the Review, the “directing” effect has also been powerfully applied in addressing challenges of site-selectivity, particularly in areas such as C–H bond functionalization where many challenges remain. Chemists seeking to address these challenges in future should remember that several very common motifs can bear charge when protonated (e.g., an amine) or deprotonated (e.g., a carboxylic acid). However, the lack of a charged functionality does not rule out using ionic interactions–in a more complex system, a charged ligand may bear a counterion which can interact with the substrate through attractive non-covalent interactions or repulsive steric interactions, and examples of these are also featured. Other examples discussed should dispel some concerns over the poor directionality of ion pairing interactions: they demonstrate that this is not always important, and that the strong Coulombic force associated with an ionic interaction in a nonpolar solvent can powerfully influence the outcome of a reaction.

In Section 3, the focus was on examples in which the metal center is thought to bear a formal charge and the counterion plays a role in determining selectivity. The most common scenario is one in which the metal is cationic and engages in ionic interactions with a counteranion, although there are also important examples in which the metal center is anionic. The former situation can be difficult to define, since differentiating between a charge-separated ion pair and a bound anionic ligand can be very difficult. Nevertheless, the design principle of invoking ion pairing here is a valuable one and has led to numerous advances that were not feasible with a more conventional ligand strategy. From a design point of view, this approach is brilliant in that its invocation just requires a reasonable possibility that a metal complex will bear a positive charge at the crucial point in the cycle. This makes it amenable to widespread application, even if in some cases it may actually be acting as a ligand. One aspect which is immediately apparent from this survey is that the popularization of 3,3′-substituted BINOL-derived phosphoric acids has had a truly outsized impact on the development of this approach. Over the two decades since they were first introduced as chiral Bro̷nsted acid organocatalysts, they have firmly established themselves as a versatile and privileged scaffold in asymmetric transition metal catalysis. They form the lion’s share of chiral counterion approaches featuring cationic metals; some of this success will undoubtedly be related to their bifunctional nature which allows them to engage in ionic interactions and hydrogen bonding interactions concurrently. The dominance of this one motif makes one wonder if there are other privileged chiral anions just around the corner which could have a similar impact–an exciting prospect to consider. The recent example by Jacobsen which used a chiral anion binding catalyst to effectively render an achiral anion chiral through complexation is an interesting development, and has the potential to mobilize a switch from organocatalytic to transition metal-catalyzed applications of these catalyst types.

In terms of outlook, it becomes clear from assembling this Review that strategies that use ionic interactions have convincingly provided solutions to problems that were not achievable using conventional metal–ligand approaches. Charged intermediates are a relatively common occurrence, so it is likely that, with the precedent set by the numerous examples shown in this Review, chemists will feel increasingly confident incorporating ionic interactions into the design of catalytic systems alongside hydrogen bonding and other, less well established attractive noncovalent interactions.
